# AI and AI-powered digital twins for smart, green, and zero-energy buildings: A systematic review of leading-edge solutions for advancing environmental sustainability goals

**DOI:** 10.1016/j.ese.2025.100628

**Published:** 2025-10-15

**Authors:** Simon Elias Bibri, Jeffrey Huang

**Affiliations:** Swiss Federal Institute of Technology in Lausanne (EPFL), Institute of Computer and Communication Sciences (IINFCOM), School of Architecture, Civil and Environmental Engineering (ENAC), Media and Design Laboratory (LDM), Lausanne, Route Canontale, 1015, Switzerland

**Keywords:** Artificial intelligence, Digital twins, Smart buildings, Green buildings, Zero-energy buildings, Environmental sustainability, Built environment, Sustainable smart cities

## Abstract

Buildings are among the largest contributors to global energy consumption and carbon emissions, making their transformation essential for advancing environmental sustainability goals. Innovative technologies such as artificial intelligence (AI) and digital twins (DTs) offer powerful tools for optimizing performance in smart, green, and zero-energy buildings. However, existing research remains fragmented—AI and AI-driven DT applications are often confined to isolated functions or specific building types—resulting in a limited, non-cohesive understanding of their collective potential in the built environment. This fragmentation, in turn, has hindered the development of integrated strategies that link building-level efficiencies with the broader environmental objectives of smart cities. To address these interrelated gaps, this study conducts a comprehensive systematic review of leading-edge AI and AI-powered DT solutions applied across smart, green, and zero-energy buildings. It aims to provide a holistic understanding of how these solutions enhance environmental performance through the analysis of key building-related indicators. By synthesizing, comparing, and evaluating recent research, it examines how AI and AI-powered DT technologies facilitate integrated, system-level strategies that promote environmentally sustainable smart practices across the built environment. The study reveals that AI enhances smart buildings by enabling dynamic energy optimization, occupant-centered environmental control, improved thermal comfort, renewable energy integration, and predictive system management. In green buildings, AI contributes to greater resource efficiency, minimizes construction and operational waste, promotes the use of sustainable materials, strengthens cost estimation and risk assessment processes, and supports adaptive design strategies. For zero-energy buildings, AI facilitates multi-objective optimization, advances explainable and transparent AI-driven control systems, supports performance benchmarking against net and nearly zero-energy standards, and enables renewable energy integration tailored to diverse climatic and regulatory contexts. Furthermore, AI-powered DTs enable real-time environmental monitoring, predictive analytics, anomaly detection, and adaptive operational strategies, thereby enhancing building performance, energy optimization, and resilience. At broader spatial scales, these technologies foster interconnected urban ecosystems, advancing environmental sustainability, sustainable development, and smart city initiatives. Building on these insights, this study introduces a novel integrated framework that positions AI and AI-driven DTs as systemic enablers of environmentally sustainable smart built and urban environments, emphasizing their cross-scale convergence in promoting carbon neutrality, circular economy principles, climate resilience, and regenerative urban strategies. The findings offer actionable pathways for advancing research agendas, inform practical strategies for building and urban system design, and provide evidence-based recommendations for policymakers committed to fostering more intelligent, sustainable, and resilient urban futures. This work establishes AI and AI-driven DTs as transformative catalysts for realizing the next generation of resource-efficient, carbon-neutral, and ecologically integrated urban ecosystems.

## Highlights

• Documents leading-edge AI and AI-driven digital twin solutions for smart, green, and zero-energy buildings.
• Bridges fragmented research into a holistic framework for environmentally sustainable smart built and urban environments.
• Reveals how AI and AI-powered DTs enhance overall environmental performance across smart, green, and zero-energy buildings.
• Shows how AI-powered DTs enable real-time monitoring, predictive analytics, anomaly detection, and adaptive operational strategies.
• Positions AI and AI-driven DTs as catalysts for resource-efficient, carbon-neutral, and ecologically resilient urban ecosystems.

## Graphical abstract

**Figure:**
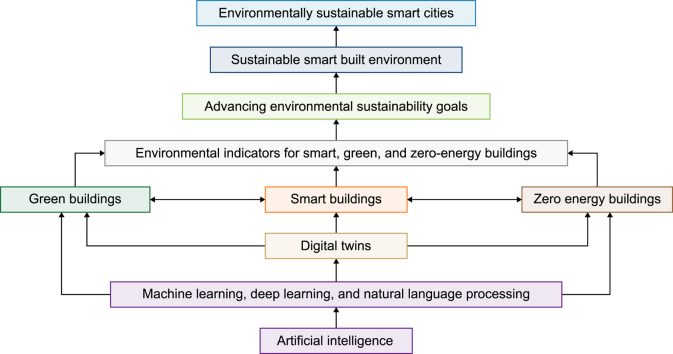

## 1 Introduction

Rapid urbanization, escalating ecological degradation, intensifying climate risks, and increasing resource constraints have made the development of sustainable smart cities a global imperative. Within this framework, as urban populations continue to expand, the carbon footprint of the built environment has grown significantly—buildings alone account for nearly 40% of global energy-related carbon dioxide (CO_2_) emissions. This positions the building sector as a critical driver for advancing the environmental goals of sustainable urban development. In this context, environmental sustainability denotes the capacity to maintain ecological systems and resource cycles in balance by ensuring that human activities remain within the regenerative and absorptive limits of the natural environment. The building industry is among the largest consumers of natural resources and one of the major contributors to ecological pressures. Buildings are responsible for considerable energy use, environmental impacts, material consumption, and waste generation throughout their life cycle, from construction and operation to eventual demolition.

In response, recent technological innovations—particularly Artificial Intelligence (AI), Artificial Intelligence of Things (AIoT), Urban Digital Twins (UDTs), and their convergence—have created new opportunities to tackle pressing environmental challenges in sustainable urban and built environments [[1], [2], [3], [4], [5], [6]]. These advanced technologies enable dynamic, data-driven decision-making capable of optimizing energy consumption and reducing carbon footprints, as well as enhancing the performance, resilience, and adaptability of the built environment. Reflecting this technological shift, sustainable smart cities are increasingly prioritizing environmental strategies across key domains, including renewable energy, resource management, transportation management, pollution control, waste management, ecosystem protection, biodiversity conservation, and climate resilience [[3], [4], [7], [8], [9], [10], [11], [12], [13], [14], [15]], thereby aligning more closely with the environmental objectives of the Sustainable Development Goals (SDGs) [[16], [17], [18]]. This transition is marked by a growing emphasis on smart energy grids, adaptive energy management, renewable energy integration, decarbonization, and pollution mitigation [19,[20], [21], [22],^23^,^24^].

By integrating AI with the Internet of Things (IoT), DTs, Building Information Modeling (BIM), and Cyber-Physical Systems (CPS) across diverse building typologies and domains [2,[5], [25], [26], [27], [28], [29], [30], [31], [32], [33], [34], [35], [36]], the Architecture, Engineering, and Construction (AEC) industry is undergoing rapid transformation. This shift is driving more intelligent, adaptive, and sustainable practices across the sector [[37], [38], [39], [40], [41]]. Within this dynamic landscape and transformative shift, key building typologies—namely smart buildings, green buildings, and Zero-Energy Buildings (ZEBs)—have emerged as critical arenas where AI, AIoT, DTs, and CPS converge. These technologies drive impactful environmental outcomes, support climate mitigation efforts, and advance sustainable development objectives.

AI, particularly Machine Learning (ML) and Deep Learning (DL), is transforming smart buildings by enhancing automation, energy efficiency, occupant comfort, and safety, among others. Equipped with advanced systems and relying heavily on AI, IoT, and real-time data, smart buildings involve monitoring and control operations such as heating, ventilation, air conditioning, lighting, and security to improve efficiency, comfort, and performance. AI-driven systems leverage data generated via IoT devices, DTs, and predictive analytics to optimize energy consumption, improve photovoltaic self-consumption, and enable sustainable carbon peak management (e.g., Ref. [[42], [43], [44], [45]]). Smart vision and DL contribute to automation in construction and intelligent building-transportation integration, facilitating real-time monitoring and efficiency improvements [46,47]. AI-powered occupant profiling enhances thermal comfort and energy savings, while predictive models support fire safety, health applications, and personalized decision-making in smart environments [[48], [49], [50], [51]]. Furthermore, AI-driven control frameworks, such as borehole thermal energy storage and wastewater heat recovery, optimize energy distribution, and ML-based predictive models aid in cost-efficient smart building operations [52,53]. These advancements underscore AI's critical role in transforming smart buildings into dynamic, self-optimizing systems that leverage predictive analytics, adaptive control strategies, and autonomous decision-making. These systems advance environmental objectives by optimizing resource use, minimizing operational costs, anticipating maintenance needs, and continuously adapting to occupant behaviors and environmental conditions.

Regarding AI integration into green buildings, it reflects a parallel focus on sustainable design, green innovation, and resource and lifecycle optimization. These structures are designed to minimize environmental impact through energy efficiency, sustainable materials, water conservation, and waste reduction. Increasingly, AI is being applied to both the design and performance enhancement of green buildings, with an emphasis on improving environmental and operational effectiveness and enabling multi-level integration [9,[54], [55], [56]] and energy system optimization, including biogas energy supply modeling and multi-objective energy optimization approaches [[57], [58], [59], [60]]. AI also contributes to sustainability evaluation and performance analysis, including energy consumption assessment and cost reasonableness prediction in green building projects [61,62]. In addition, AI is increasingly applied in cost estimation, risk assessment, and overall project evaluation in green buildings, supporting more informed decision-making and optimized resource allocation [[63], [64], [65], [66]]. ML techniques are further applied to evaluate thermal conductivity improvements using nano-insulations [67] and to enhance thermal comfort using random forest (RF) and non-dominated sorting genetic algorithm II (NDSGA) [58]. Finally, hybrid AI models that combine neural networks and decision support tools are employed to assess the waste management and energy-saving potential in green buildings [68].

While green buildings primarily emphasize passive design strategies, material sustainability, and overall environmental impact reduction, ZEBs, net-zero energy buildings (NZEBs), nearly-zero energy buildings (nZEBs), and positive energy buildings (PEBs) explicitly focus on achieving operational energy neutrality or surplus. In this area, AI plays a critical role through predictive optimization, smart energy management, and renewable energy integration. ZEBs, nZEBs, NZEBs, and PEBs are defined as follows, each uniquely contributing to environmental goals [69]:

• ZEBs achieve net-zero annual operational energy by balancing on-site renewable generation with consumption, without relying on external sources.
• nZEBs have very low energy demand, mostly supplied by high-efficiency systems, with only a small part coming from external sources.
• NZEBs achieve a net-zero annual energy balance through on-site renewable generation and minimal external energy imports.
• PEBs produce more energy than they consume annually, generating surplus energy that can be exported to the grid.

Research focuses on load forecasting and energy management, using ML and neural network models to predict building energy demand, separate heating, ventilation, and air conditioning (HVAC) loads, and support energy optimization in smart and NZEB homes [[70], [71], [72]]. AI is also applied to the design and performance optimization of energy systems, including hybrid optimization methods, surrogate modeling, and battery-based autonomy enhancement to improve comfort, efficiency, and building performance [33,[73], [74], [75]]. Moreover, research also explores control strategies using AI, including application programming interface (API)-integrated smart grid controls, predictive neural network-based energy control, and adaptive systems for dynamic building operations [[76], [77], [78]]. Moreover, several studies provide more comprehensive insights into AI applications for ZEBs and PEBs, system integration, and research frontiers [[79], [80], [81]]. Further, AI is leveraged in DT applications and smart city frameworks to support ZEB assessment and integration into sustainable urban systems [69].

The convergence of AI and DT technologies further enhances the potential for simulation-based optimization, continuous performance monitoring, and the integration of smart cities. This synergy enhances building performance, sustainability, and operational efficiency. This is evidenced by recent research on AI-enabled DT for energy consumption prediction, focusing on improving energy modeling, forecasting loads, and optimizing building energy use in real time [28,29,34]. Other studies utilize AI and DTs for thermal comfort monitoring, providing frameworks for maintaining occupant well-being while optimizing HVAC systems and energy balance [26]. The synergy of AI and DTs is also evident in predictive analytics and energy management, where CPS and AIoT infrastructures contribute to monitoring emissions, forecasting CO_2_ equivalents, and improving asset performance across the building lifecycle [25,27,82,83]. In urban-scale applications, DTs are combined with AI and IoT to support sustainable smart city and building environments [5]. In addition, comprehensive studies focus on assessing the characteristics, applications, and challenges of AI-powered DTs in building performance simulation and intelligent built environments [84,85].

Despite the growing interest in smart, green, and zero-energy building (SGZEB) typologies—and the emerging points of convergence enabled by AI and DTs—the current literature remains fragmented, often analyzing AI and AI-driven DT applications in isolation or focusing on specific types of buildings and aspects of environmental sustainability. Specifically, while existing review studies have explored various applications of AI in smart buildings, they often focus on certain domains such as AIoT integration, ML-driven energy management, DT applications, thermal comfort optimization, and energy efficiency [42,53,[86], [87], [88]]. Other review studies have examined AI techniques for green buildings [89,90], AI-driven carbon emission forecasting [91], computational intelligence for HVAC system optimization [92], and AI and DT applications for zero-energy, net-zero energy, and positive energy buildings [27,69,80]. The roles of AI in fire safety [50], predictive control-based energy management [45], and building performance simulation [84] have also been reviewed. While these review studies provide valuable insights, they often adopt a fragmented approach, addressing AI and/or DT solutions in isolation rather than as part of a system-level approach. There is also a lack of a unified framework that consolidates AI- and DT-driven strategies across SGZEB typologies through the lens of environmental sustainability, providing an integrated and holistic perspective.

To address these gaps, this study conducts a comprehensive systematic review of leading-edge AI and AI-powered DT solutions applied across smart, green, and zero-energy buildings. It aims to provide a holistic understanding of how these solutions enhance environmental performance through the analysis of key building-related indicators. By synthesizing, comparing, and evaluating recent research, it examines how AI and AI-powered DT technologies facilitate integrated, system-level strategies that promote environmentally sustainable practices across the built environment. It highlights the interconnectedness among AI and DT technologies, building typologies, and environmental objectives in shaping more adaptive, efficient, resilient, and low-impact building systems. The study, by bridging these domains, offers deeper insights into the transformative potential of AI and AI-driven DTs in advancing sustainable smart buildings and establishes a structured foundation for future research and practical implementation. To achieve the overall aim and meet the objectives, this study is guided by the following research questions (RQs):

RQ1: How is AI currently applied to enhance the environmental performance of smart buildings?

This question focuses on applications of AI in intelligent building management systems, predictive analytics, and energy optimization in smart buildings.

RQ2: What role does AI play in optimizing the environmental performance of green buildings?

This question addresses AI's integration in sustainable design, green certifications, decision-support tools, and project management processes such as cost estimation and risk assessment.

RQ3: How does AI contribute to improving the environmental performance of zero-energy, net-zero-energy, and nearly-zero-energy, and positive-energy buildings?

This question examines the intersection of AI with energy-efficient building technologies, renewable energy integration, and performance benchmarking in highly energy-efficient buildings.

RQ4: In what ways can AI-powered DT technologies be leveraged to advance environmental goals in building systems or environments?

This question investigates the potential of AI-enabled DT frameworks for real-time monitoring, simulation, and system-level sustainability assessment.

RQ5: How can AI- and DT-enabled SGZEBs align with and contribute to advancing environmental sustainability, sustainable development, and sustainable smart cities?

This question explores the role of AI and DT integration in scaling building-level sustainability practices to broader urban and global agendas.

This study makes several concrete contributions to the evolving discourse on AI, DTs, and environmentally sustainable smart built environments:

• Conceptual integration of AI and DT for SGZEBs: The study develops a cohesive, principle-based framework that links AI and DT technologies across SGZEBs, highlighting their synergistic potential for advancing environmental sustainability in the built environment.
• Cross-typology reinforcement: It demonstrates how data-driven control in smart buildings, circularity in green buildings, and renewable integration in ZEBs can reinforce each other in a continuous feedback loop by analyzing how sustainability principles flow between different building typologies.
• Bridging building and urban scales: It positions buildings not as isolated assets but as interconnected actors within wider urban systems, demonstrating how AI–DT integration enables a systemic transition from building-level optimization to urban-level environmental performance and resilience.
• Advancing theory and practice: It synthesizes fragmented AI- and DT-related research into a unified perspective, providing both theoretical grounding (linking simulations, models, and real-time applications) and practical guidance for researchers, practitioners, and policy-makers.
• Strategic alignment with sustainability agendas: It aligns the AI–DT framework with broader environmental and societal goals, including net-zero transitions, circular economy adoption, climate resilience, and SDGs.
• Bridging innovation and implementation: It connects technological advancements in AI and DT with real-world application pathways, providing practical recommendations for diverse stakeholders and supporting the systemic transformation of buildings and cities towards environmental sustainability.

This study is structured as follows: Section 2 provides a survey of related work, identifying current research trends and highlighting key gaps in the integration of AI technologies in sustainable smart buildings. Section 3 outlines the systematic literature review methodology, including its integration with bibliometric analysis to ensure a comprehensive and structured exploration of the field. Section 4 presents the outcomes of the bibliometric analysis, offering a quantitative overview of research patterns and trends. Section 5 details the results of the tabulated thematic analysis and the thematic synthesis of the literature on the three building typologies and their integration with AI and DTs. It also introduces, illustrates, and elaborates on the novel integrated framework developed to drive environmental goals across SGZEBs. Additionally, it examines the relationship between the proposed framework and the broader objectives related to environmental resilience, sustainable development, and sustainable smart cities. Section 6 provides a comprehensive discussion, summarizing the key findings in relation to the research questions, offering a comparative analysis with previous related studies, exploring implications for research, practice, and policymaking, addressing the identified challenges, barriers, and methodological limitations, and suggesting future research directions. Finally, Section 7 concludes the study by synthesizing the key insights, emphasizing the contributions made, and offering final reflections on the future prospects for sustainable smart built environments.

## 2 Related work

AI has emerged as a key driver in the development of smart and sustainable buildings, offering transformative solutions for automation, energy optimization, improved performance, and achieving net-zero energy goals. Various review studies have examined AI's applications in the built environment, yet they often adopt a fragmented approach, focusing on specific domains or technologies rather than providing a comprehensive framework that integrates AI-driven strategies for environmental sustainability. This section synthesizes existing literature on AI applications in smart and sustainable buildings, highlighting key contributions and limitations that shape the research gap.

AI-driven automation in smart buildings has focused on integrating AI and AIoT to enhance operational efficiency, safety, and occupant comfort. Sleem and Elhenawy [42] provided an overview of AIoT technologies in smart buildings, emphasizing their role in optimizing building functionality, reducing energy consumption, and improving security. However, their study also addressed challenges such as data privacy concerns and interoperability issues, which hinder large-scale AIoT adoption. Similarly, Qolomany et al. [87] reviewed the role of ML and big data in smart building automation, detailing how predictive analytics can enhance real-time decision-making.

AI applications in energy management have gained significant attention, particularly through the use of ML models and DT technology. Alanne and Sierla [53] examined ML applications in smart buildings, focusing on how autonomous learning processes can enable adaptive energy management. Their study also discussed the role of DTs as AI-powered training environments for optimizing energy use. In contrast, Wang et al. [43] explored digital twin applications specifically for carbon peak management. They highlight their ability to monitor emissions in real time and model net-zero energy strategies.

Several studies have focused on the potential of AI in green and sustainable buildings, particularly in optimizing resource efficiency and minimizing ecological impact. Rodríguez-Gracia et al. [89] conducted a bibliometric analysis of AI applications in green and smart buildings, identifying key themes such as energy optimization, structural stability, and reduction of environmental impact. Debrah et al. [55] complemented this perspective by examining AI in green buildings through a mixed-methods approach, combining bibliometric and systematic analyses to present a comprehensive overview of state-of-the-art research. Their study identifies key research trends, major hotspots, and knowledge gaps, highlighting future directions including the integration of DTs, AIoT, blockchain, robotics, four-dimensional printing, and considerations of legal, ethical, and moral implications in AI-enabled green buildings. Wu et al. [90] extended this line of inquiry by shifting the focus from AI applications in buildings to AI as the central driver of green building technology innovation (GBTI). Through bibliometric and dynamic topic modeling analyses, they map the knowledge structure, thematic evolution, and emerging research paradigms of AI-driven GBTI. Hua et al. [91] examined the role of AI in forecasting and managing building carbon emissions, highlighting how AI-driven models enhance accuracy by up to 20% compared to traditional methods. Their study demonstrates that AI-based real-time monitoring and adaptive management strategies can reduce carbon emissions by up to 15%, improve energy efficiency by 25%, and lower operational costs by 10%.

Integrating elements of smart building technology but primarily oriented toward green building sustainability, Adewale et al. [93] presented a systematic review of AI applications across the sustainable building lifecycle. They focused on how AI can enhance energy efficiency, support predictive maintenance, and improve design simulation processes. The review underscores the use of advanced ML and DT technologies to enable data-driven decision-making and real-time performance optimization, while also identifying key barriers to implementation, including high costs, data security concerns, and technical complexity.

Mousavi et al. [80] reviewed AI applications for net-zero and positive energy buildings (NZEBs), examining how data-driven prediction and optimization models can enhance energy efficiency and optimize renewable energy generation. Their study highlights the importance of integrating AI into energy management systems to achieve sustainability targets. Meanwhile, Bibri et al. [2] proposed a DT-based framework for assessing and optimizing ZEB performance within sustainable smart cities. The study identifies key trends in the integration of DTs and ZEBs, emphasizing the increasing influence of AI, IoT, and cyber-physical systems (CPS). It also highlights specific research patterns that illustrate their synergistic interaction and their role in driving this convergence. Moreover, it demonstrates how UDTs enhance ZEBs' energy management and performance by improving energy efficiency, facilitating the integration of renewable energy, and reducing carbon emissions through real-time monitoring, advanced data analytics, predictive maintenance, and operational optimization.

HVAC systems represent one of the most energy-intensive aspects of buildings, and AI-driven strategies have been widely studied to enhance their efficiency. Sha et al. [92] presented a detailed review of computational intelligence techniques for optimizing HVAC system design, outlining how AI-driven models can improve energy efficiency while maintaining indoor comfort. Merabet et al. [86] further explored AI-based thermal comfort control, demonstrating that AI-assisted building control systems can balance energy savings with occupant comfort through real-time adaptive mechanisms. Similarly, Yussuf and Asfour [88] reviewed AI applications across various stages of the building lifecycle. They highlight predictive control, energy benchmarking, and fault detection as critical components of AI-driven HVAC optimization. While these studies contribute to AI's role in energy-efficient climate control, they primarily address individual HVAC improvements without considering AI's broader role in sustainable building design, operation, and user-centric optimization.

AI applications in building performance and safety management have also been explored. De Wilde [84] examined AI's integration with building performance simulation, discussing how AI-enhanced models can improve predictive accuracy and optimization capabilities in building operations. However, they focus primarily on simulation methodologies without addressing AI's role in sustainability-driven performance optimization. Zeng and Huang [50] investigated AI's application in fire safety design, demonstrating how AI-driven risk assessment models can enhance early fire detection and emergency response.

Although prior studies have provided extensive and diverse reviews of AI applications in smart and sustainable buildings, they often adopt a domain-specific approach by examining AI models and techniques in isolated contexts such as energy management, HVAC optimization, or DT simulations. Existing reviews on AI-driven energy efficiency primarily focus on technical improvements without fully addressing sustainability principles, while studies on AI for net-zero buildings and green construction often lack insights into AI's role in automation and lifecycle optimization. Furthermore, although DT applications have been explored in energy monitoring and carbon management, their potential in supporting holistic AI-driven sustainability frameworks remains underdeveloped.

This comprehensive systematic review addresses these gaps by providing a holistic perspective on the role of AI and AI-driven DT in achieving environmental objectives in smart and sustainable buildings. Unlike previous studies, this review adopts a cross-system approach, integrating AI and AI-driven DT applications across various building functions, typologies, and lifecycle stages to examine their collective environmental impact. Through this synthesis, the study aims to establish a structured framework for future research and practical implementations of both AI and AI-driven DT in environmentally sustainable smart built environments.

## 3 Research methodology

This study adopts a mixed-method research design that integrates a systematic literature review with bibliometric analysis to investigate how AI and AI-driven DT technologies contribute to environmental goals across SGZEBs. The systematic review serves as the central methodological framework, enabling an in-depth and structured examination of peer-reviewed studies that focus on the intersection between AI and DT technologies and sustainability objectives in the built environment. It addresses all five research questions guiding the study. Complementarily, the bibliometric analysis provides a quantitative lens to assess research trends, emerging patterns, and knowledge gaps in the interdisciplinary field as identified in this study, which helps situate it within the evolving scholarly landscape. It provides a macro-level overview of the research landscape and dynamics by identifying intellectual trends, thematic concentrations, and co-authorship networks, thereby enhancing the foundation of the study.

The systematic review facilitates a structured and qualitative synthesis of theoretical, empirical, and experimental findings by specifically identifying how advanced technologies—such as AI, ML, CV, NLP, GenAI, AIoT, and DTs—are being leveraged to enhance different aspects and objectives of environmental resource management across three major building typologies. Simultaneously, the bibliometric analysis, conducted using VOSviewer, maps the research field by analyzing keyword co-occurrences, citation networks, and thematic clusters to reveal the structure and evolution of the research field from 2020 to 2025. This dual-method approach offers both trend-level breadth and thematic depth, essential for a nuanced understanding of how AI and DT support environmental efforts across diverse architectural, technical, and operational contexts of buildings. It captures the trajectory of recent research while distilling insights relevant to the systematic integration of AI in environmentally sustainable smart building practices. It also ensures that both qualitative and quantitative dimensions of the literature are addressed, allowing for a comprehensive synthesis that encapsulates the multifaceted ways in which AI and DT capabilities are being harnessed to improve the environmental performance of building systems.

### 3.1 Research design

**Fig. 1:**
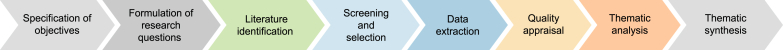
A multi-phase structured process of the systematic literature review.

The study was designed as a multi-phase, iterative process structured around the standard stages of a systematic literature review (Fig. 1). At its core, the goal is to map how AI and AI-driven DT technologies contribute to environmental sustainability through key indicators across SGZEBs. Accordingly, the research systematically examined applied solutions to energy efficiency, renewable integration, carbon footprint reduction, waste minimization, water efficiency, indoor environmental quality, and thermal comfort across these building typologies. This thematic mapping served as the analytical groundwork for identifying cross-cutting insights and guiding the development of the integrated framework.

The research design employs a thematic approach, which is particularly suitable for interdisciplinary inquiries that span environmental science, architectural design, engineering, sustainability, and technology. It aligns with the study's objective of developing a comprehensive understanding of AI's role in optimizing environmental processes and practices within the built environment. Each study was reviewed not only in terms of its technological focus but also for its contribution to one or more of the sustainability indicators across the three building typologies. This structured yet flexible categorization informed the development of comparative tables that map the intersection of AI, AI-driven applications, and sustainability objectives. In addition, the research design allowed for the identification of recurring themes, potential synergies, and underexplored areas. This, in turn, supported the development of an integrated framework that highlights key convergences in AI-enabled sustainable building practices.

### 3.2 PRISMA and literature search strategy

**Fig. 2:**
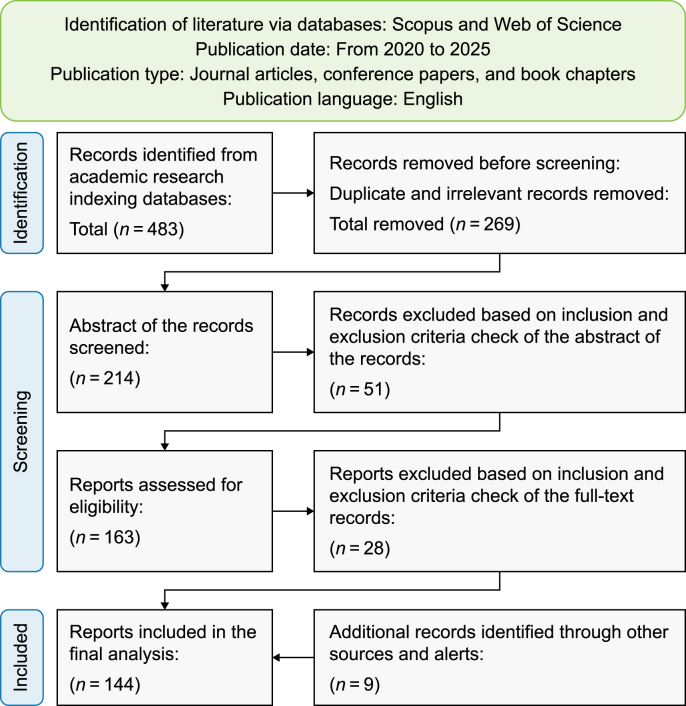
The PRISMA flowchart for literature search and selection.

The preferred reporting items for systematic reviews and meta-analyses (PRISMA) framework (Fig. 2) was adopted to ensure transparency and rigor in the literature search and selection process. The PRISMA flow diagram guided the documentation of the review process, from the initial identification of records through the final inclusion of eligible studies. This process included the systematic removal of duplicates, assessment of relevance based on titles and abstracts, full-text review, and quality appraisal.

The literature search was conducted across two major academic databases—Scopus and Web of Science—chosen for their breadth of interdisciplinary coverage and inclusion of authoritative sources of evidence. These databases were selected to ensure comprehensive retrieval of scholarly works pertinent to the multifaceted topic addressed in this study. To enhance specificity and relevance, search queries were formulated using tightly coupled keyword combinations, applied across the title, abstract, and keyword fields, to target both the technological dimensions of AI and the environmental objectives specific to the three building types. The curated keywords and their combinations guiding the literature search were systematically organized into thematic categories. These categories were designed to capture the breadth and depth of existing knowledge while aligning with the study's scope. The selection of keyword combinations reflected the multidimensional nature of this research and ensured a thorough exploration of related literature across various fields. Boolean operators were strategically used to refine the search scope and construct complex search strings that captured the intersectionality of various domains. Accordingly, among the query structures employed were:

(“Artificial Intelligence” OR “AI” OR “Machine Learning” OR “Deep Learning”) AND (“Smart Buildings” OR “Intelligent Buildings”)
(“Artificial Intelligence” OR “AI” OR “Machine Learning” OR “Deep Learning”) AND (“Green Buildings” OR “Sustainable Buildings”)
(“Artificial Intelligence” OR “AI” OR “Machine Learning” OR “Deep Learning”) AND (“Zero Energy Buildings” OR “Net Zero Energy Buildings” OR “Nearly Zero Energy Buildings” OR “Positive Energy Buildings”)
(“Artificial Intelligence” OR “AI” OR “Artificial Intelligence of Things” OR “AIoT”) AND (“Digital Twins” OR “DT”) AND (“Smart Buildings” OR “Green Buildings” OR “Zero-Energy Buildings”)
(“Smart Buildings” OR “Green Buildings” OR “Zero-Energy Buildings”) AND (“Environmental Indicators” OR “Performance Indicators” OR “Sustainability Indicators”)
(“Artificial Intelligence” OR “AI” OR “Artificial Intelligence of Things” OR “AIoT”) AND (“Environmental Sustainability” OR “Sustainable Development”) AND (“Smart Cities” OR “Sustainable Smart Cities” OR “Built Environment”)
(“Artificial Intelligence” OR “AI”) AND (“Energy Efficiency” OR “Thermal Comfort” OR “Performance” OR “Architectural Design”) AND (“Buildings” OR “Building Environment”)

These queries were iteratively refined to exclude irrelevant results while maximizing the inclusion of studies that address the convergence of AI and AI-driven DT technologies and environmental integrity in building systems. The review timeframe was set between 2020 and 2025 to capture the most recent and impactful developments in AI-driven environmental applications in the building sector. This period, especially 2022–2025, reflects a surge in AI research in the AEC sector, especially in the wake of global climate action and technological advances in AI, AIoT, and DTs. This strategy yielded a broad but thematically focused set of records, ensuring that the final sample reflects the diversity of AI and AI-driven DT applications across SGZEB typologies.

### 3.3 Inclusion and exclusion criteria

The inclusion and exclusion criteria were developed to ensure the relevance, quality, and coherence of selected studies. Only studies published in English between 2020 and 2025 were included, focusing on peer-reviewed journal articles, conference proceedings, scholarly book chapters, and policy documents. Eligible studies explicitly primarily addressed AI, ML, or DL and related DT applications in one or more of the three building typologies, with a direct connection to at least two environmental indicators.

Studies were excluded if they did not meet the time frame, lacked a focus on AI, AI-driven DT, or environmental sustainability; addressed buildings only peripherally; did not directly address AI or AI-driven DT applications in smart, green, or zero-energy buildings; or were non-peer-reviewed sources such as editorials, white papers, and grey literature. Studies that dealt solely with economic modeling, structural engineering unrelated to environmental performance, or speculative conceptual essays without empirical grounding were also excluded. This filtering process helped refine the dataset to include only high-quality, thematically relevant contributions that informed the study's research questions and comparative analysis.

### 3.4 Data extraction and quality appraisal

Following the selection process, a structured data extraction protocol was implemented to capture consistent and essential information across the included studies. Each study was reviewed for key metadata, including authorship, publication year, methodological approach, AI techniques applied, AI-driven DT solution, building typology, and environmental indicators addressed. Detailed notes were taken on objectives, experimental or modeling techniques, theoretical or conceptual emphases, outcomes or findings, and limitations or challenges. This process ensured consistency and accuracy while enabling cross-comparison among studies. A parallel quality appraisal was conducted to assess the methodological rigor and relevance of each study in relation to the research questions and transparency in reporting. Only those studies that demonstrated a clear application of AI and AI-driven DT in one or more environmental indicators were retained for further analysis. Studies with ambiguous methodologies, limited scope, or unclear relevance were excluded from the final outcome to maintain the focus of the review.

### 3.5 Data analysis

The data analysis was organized thematically, guided by the core environmental indicators and conceptual categories identified during the research design phase. Accordingly, each study was categorized based on its alignment with building typologies and one or more of these indicators. Studies were grouped under each building type and evaluated for convergence and divergence in AI models and applications. To systematically map these associations, a set of comparative tables was developed, each corresponding to SGZEBs. These tables function as a comparative matrix, enabling structured analysis across the three building typologies. The matrix serves not only as an analytical tool but also as a foundation for the subsequent framework development by visually organizing AI applications in relation to environmental indicators.

Cross-cutting themes such as DT integration and AI in AEC, and GenAI in architectural design were also examined. Bibliometric results were used to validate emerging themes, visualize research clusters, and support the identification of dominant and underexplored areas. Thematic overlaps were documented and explored in the discussion section, while gaps in the literature were flagged as areas for future research.

### 3.6 Synthesis of findings and framework development

The synthesis stage involved integrating insights from both the thematic and bibliometric analyses into a coherent narrative to build a comprehensive understanding of the topic on focus. This process entailed systematically comparing AI applications across different building typologies, identifying commonalities, distinctions, and emerging trends in relation to key environmental indicators. Through this comparative synthesis, patterns of AI convergence, such as shared applications in energy efficiency, predictive analytics, and automation, were highlighted, alongside areas of divergence where AI's role is more specialized for certain building types. In addition, underexplored intersections between AI techniques and sustainability objectives were identified, revealing gaps and opportunities for future research.

These synthesized insights informed the development of a novel integrated framework that visually and conceptually represents the alignment of AI technologies with environmental indicators across building typologies. The framework captures cross-cutting applications while also mapping distinct roles AI plays in each building type, serving as a tool for identifying pathways towards comprehensive integration of AI in the sustainable built environment. The study moves beyond fragmented understandings of AI applications in buildings to offer a holistic, actionable view by synthesizing findings across technological, environmental, and typological dimensions. The framework contributes to academic discourse and practical applications, supporting informed decision-making in building design, policy development, and urban planning.

## 4 A bibliometric analysis of smart, green, and zero-energy buildings: mapping artificial Intelligence's role in environmental sustainability in the built environment

To situate this study within the evolving scholarly landscape, the bibliometric analysis provides a quantitative lens through which to assess research trends, emerging patterns, and knowledge gaps across the interdisciplinary domain intersecting building typologies, advanced technologies, and environmental solutions. It was employed to map and visualize the landscape of integrating AI, ML, DL, CV, NLP, and Generative AI in advancing SGZEBs as part of broader environmental strategies. The objective was to uncover the thematic structure, dominant clusters, and emerging trends in this rapidly burgeoning field, thereby providing context and direction for the integrated tabulated analysis, qualitative synthesis, and framework development that follow.

The bibliometric analysis was conducted using VOSviewer version 1.6.17, a specialized tool for visualizing bibliometric networks. A comprehensive dataset was compiled in March 2025, targeting peer-reviewed articles published between 2020 and 2025, a timeframe selected to capture recent advancements and emerging research directions. The dataset spans multiple disciplines, including engineering, environmental science, energy, computer science, architectural design, construction, and building technology. A total of 678 documents were retrieved from three major sources: Scopus (88), Web of Science (47), and ScienceDirect (543). The bibliometric dataset was systematically retrieved from Scopus and WoS, which served as the core indexing databases for bibliometric analysis. ScienceDirect, by contrast, was used primarily as a full-text repository for screening and contextual reading, rather than as an indexing source. The higher number of publications visible on ScienceDirect reflects Elsevier's hosting of a wide range of journals, not all of which are indexed in Scopus or Web of Science. The search strategy adopted was designed to be both targeted and comprehensive, combining key terms related to AI subdomains, building typologies, and environmental indicators. Metadata, including titles, abstracts, author keywords, and citation data, were extracted for analysis.

**Fig. 3:**
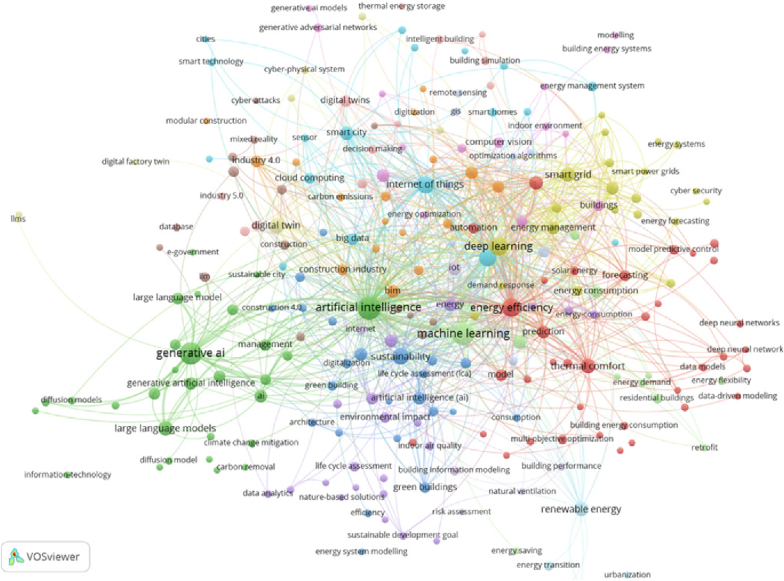
Result of the term co-occurrence analysis.

VOSviewer was used to conduct a term co-occurrence analysis, identifying frequently used terms and key themes that dominate the literature on AI in sustainable smart buildings. Fig. 3 displays a visual map of these co-occurring terms, where the size of each node represents term frequency, and the proximity between nodes indicates the strength of their co-occurrence. Different clusters are distinguished by color, reflecting thematic groupings within the research landscape.

The analysis identified 15 thematic clusters and 249 frequently occurring terms, each revealing a different facet of how AI is being applied to enhance the environmentally sustainable built environment.

Cluster 1 (36 items) is focused on artificial neural networks (ANNs), generative adversarial networks (GANs), automation, smart buildings, solar energy, indoor air quality, energy efficiency, and thermal comfort. This cluster highlights the integration of AI-driven energy management tools in smart buildings, particularly those leveraging predictive modeling and neural learning to enhance operational efficiency and occupant comfort.

Cluster 2 (29 items) reflects growing research on AI, Generative AI, diffusion models, large language models (LLMs), and AIoT applications targeting climate change mitigation, carbon removal, and SDGs. It shows how advanced generative architectures are being deployed to model, simulate, and optimize environmental performance in urban systems.

Cluster 3 (23 items) addresses themes such as sustainability, construction 4.0, energy system modeling, life cycle assessment (LCA), and digitalization, with a strong presence of terms related to green buildings and the built environment. This cluster highlights the application of AI and ML in monitoring resource consumption and enhancing energy performance through data-driven design strategies.

Cluster 4 (21 items) includes keywords such as carbon footprint, BIM, deep reinforcement learning, cybersecurity, and optimization. It focuses on DL approaches in managing energy demand and enhancing energy security, especially through integration with smart grids and CPS.

Cluster 5 (21 items) covers ZEBs, climate change, energy modeling, urban sustainability, waste management, and emissions reduction. This cluster represents the convergence of AI-enabled modeling tools and sustainability goals in designing buildings that meet net-zero or near-zero energy targets.

To further interpret these findings, each thematic cluster identified in the bibliometric analysis can be directly linked to specific advanced technologies. Cluster 1's focus on ANNs, GANs, and automation highlights the role of ML, DL, and AI-driven optimization in predictive building management. Cluster 2's emphasis on GenAI, LLMs, and AIoT reflects the integration of cutting-edge AI architectures with IoT systems to address climate change mitigation and SDG-related objectives. Cluster 3's themes of sustainability, LCA, and digitalization align closely with AI- and ML-enabled decision support for green building lifecycle performance. Cluster 4's inclusion of BIM, deep reinforcement learning, and cybersecurity demonstrates the role of DL and DT frameworks in optimizing energy demand while ensuring system resilience. Finally, Cluster 5's focus on ZEBs, emissions reduction, and urban sustainability illustrates how AI-powered simulation and modeling tools are being leveraged for net-zero and positive-energy building strategies. This linkage between thematic clusters and enabling technologies provides a clearer map of how AI, ML, CV, NLP, Generative AI, AIoT, and DTs are advancing the environmental performance of different building typologies.

**Fig. 4:**
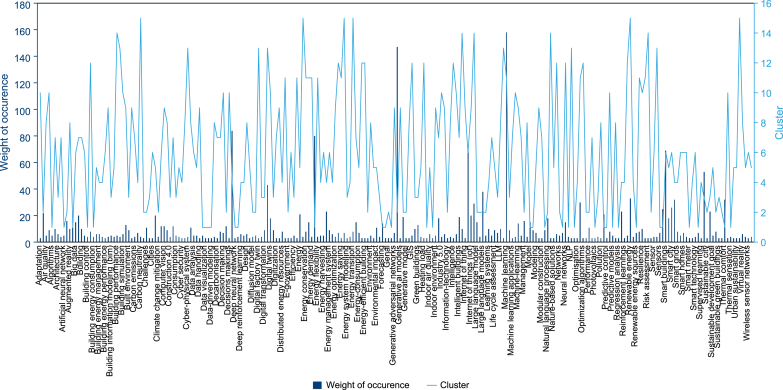
The relationship between different clusters and the weight of occurrence.

Fig. 4 illustrates the interrelationships among the 15 identified clusters, revealing how different strands of research are interconnected. The relative size of each cluster indicates the weight of occurrence, serving as a proxy for the dominance of each research theme in the current literature. Clusters 1 and 2 dominate the landscape, showing a strong emphasis on AI strategies for energy optimization, climate adaptation, and smart building automation. Clusters 3 and 4 underscore AI's contributions to lifecycle performance, carbon reduction, and security in green building contexts. Cluster 5 presents evidence of a focused yet still developing body of work on ZEBs, highlighting both the promise and gaps in this area. Overall, this clustering indicates the growing but uneven integration of AI across the three building typologies, with smart buildings receiving the most attention, followed by green buildings, and lastly ZEBs, which remain comparatively underexplored.

**Fig. 5:**
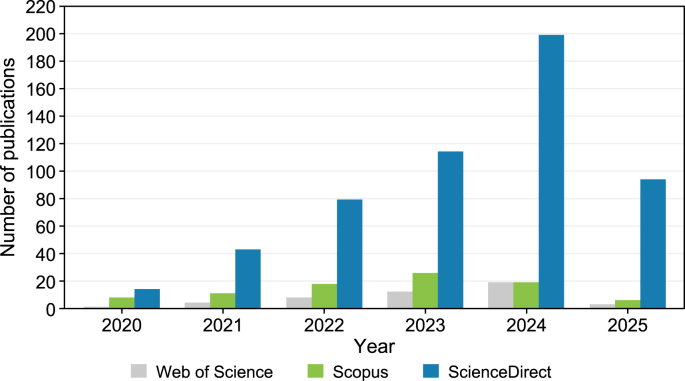
Number of publications from 2020 to 2025 across three databases (Web of Science, Scopus, and ScienceDirect).

Between 2020 and early 2025, the scholarly output on AI, Generative AI, ML, DL, CV, and NLP in the context of advancing smart, green, and zero-energy buildings as environmental solutions has grown significantly (Fig. 5). While publications were relatively sparse in 2020—with only 1 article indexed in Web of Science, 8 in Scopus, and 14 in ScienceDirect—the following years witnessed a steady rise. In 2021, the numbers climbed to 4, 11, and 43, respectively, reflecting growing academic engagement with AI applications in sustainable smart building practices. This upward trend continued into 2022, which saw 8 publications in Web of Science, 18 in Scopus, and 79 in ScienceDirect, suggesting the field was gaining traction across engineering, energy, and environmental science disciplines.

A sharper increase was evident in 2023, with 12 publications indexed in Web of Science, 26 in Scopus, and 114 in ScienceDirect. This marked a point at which AI's environmental potential in the built environment began to draw more focused attention. The most notable surge, however, occurred in 2024. That year, publications peaked at 19 in both Web of Science and Scopus, and an impressive 199 in ScienceDirect, highlighting a significant expansion of interdisciplinary research. This dramatic growth reflects an intensifying interest in AI's capacity to optimize energy systems, support predictive building operations, enable net-zero strategies, and contribute to broader sustainability goals within smart city frameworks.

While data for 2025 only covers the first quarter (January–March), the early figures—3 publications in Web of Science, 6 in Scopus, and 94 in ScienceDirect—indicate that the momentum has not slowed. If this trajectory continues, 2025 is likely to match or even surpass the previous year's record, reinforcing the notion that this domain is not only maturing but rapidly expanding.

These trends signal two important developments. First, there is an increasing scholarly focus on AI's critical role in addressing climate-related and environmental challenges through sustainable smart building systems. Second, the presence of emerging technologies such as Generative AI, advanced neural networks, and NLP points to a diversification of AI tools being employed—not just for monitoring and control, but also for generative design, emissions forecasting, and integrated sustainability planning. Together, these shifts highlight the growing complexity and promise of AI-powered solutions in shaping the future of environmentally sustainable buildings.

Overall, the publication trend (Fig. 5) confirms the emergence of a robust and rapidly evolving research domain, where AI-powered technologies are increasingly positioned as key enablers of sustainable transformation in architecture, engineering, construction, and the built environment. This trend is substantiated by studies in the AEC sector, including Zhang et al. [37], Momade et al. [38], Rafsanjani and Nabizadeh [39], Saka et al. [40], and Mor et al. [41], which document the application of AI in sustainable building design, operation, and management. The steady rise in scholarly output reflects growing recognition of AI's potential to advance environmental performance across the three building typologies. This trend is evidenced by numerous representative studies on smart buildings (e.g., Ref. [42,[46], [47], [48], [49], [50],^52^,^53^]), green buildings (e.g., Ref. [9,51,55,59,61,[54], [62], [94],^68^,^95^,^96^]), as well as ZEBs, NZEBs, nZEBs, and PEBs (e.g., Ref. [70,71,73,76,[78], [79], [80]]). At the same time, the uneven distribution of research across building types and sustainability indicators highlights a fragmented landscape—underscoring the need for integrative frameworks that can bridge disciplinary silos and support holistic, AI-driven environmental strategies.

In summary, the bibliometric analysis reveals an increasingly dynamic and interdisciplinary research landscape focused on the intersection of AI technologies and the environmental performance of the built environment. Through the identification of thematic clusters, co-occurring terms, and publication trends, the analysis highlights both the growing momentum and existing gaps in the field. Smart buildings dominate current research, particularly in relation to AI-driven energy optimization, automation, and climate mitigation and adaptation, while green buildings and ZEBs remain comparatively underexplored. The rapid rise in scholarly output—especially from 2023 onward—demonstrates expanding interest in the application of AI, including emerging tools such as Generative AI and NLP, to address sustainability challenges. However, the fragmentation across domains and the limited integration of AI across building typologies and sustainability indicators suggest the need for a more cohesive, integrated approach. These findings provide a critical foundation for the tabulated analysis, qualitative synthesis, and integrated framework that follow, offering direction for future research and practice.

## 5 Tabulated analysis and thematic synthesis

This section presents the outcomes of the systematic review conducted to address the five research questions guiding this study. The results are presented through a tabular and typological analysis, as well as a thematic synthesis of key evidence. Table 1 presents a comprehensive thematic mapping of AI applications across various building typologies, technologies, and domains, thereby supporting RQ1 through RQ4. Table 2, Table 3, Table 4 map AI's role in advancing environmental indicators in smart (RQ1), green (RQ2), and zero-energy building typologies (RQ3), respectively. It should be noted that the studies included in Table 3 are presented as illustrative subsets, selected to demonstrate how AI applications intersect with sustainability indicators in this typology; all studies are subsequently analyzed and synthesized thematically in greater depth in the subsequent subsection. This also applies to Table 1, where AI subdomains in this typology are highlighted for illustrative purposes. Together, these results form the foundation for developing the framework in response to RQ4, and further contribute to answering RQ5 by synthesizing insights across building environments towards broader environmentally SDGs in smart cities.

**Table 1:** Mapping AI subfields and subdomains across building research areas.

**No.**	**Article title**	**AI**	**AIoT**	**ML**	**DL**	**CV**	**NLP**	**GAI**	**Reference**
**Artificial intelligence in smart buildings**									
1	Survey of artificial intelligence of things for smart buildings: A closer outlook	✓	✓	✕	✕	✕	✕	✕	[42]
2	Carbon peak management strategies for achieving net-zero emissions in smart buildings: Advances and modeling in digital twin.	✓	✕	✓	✕	✕	✕	✕	[43]
3	Artificial intelligence and smart vision for building and construction 4.0: Machine and deep learning methods and applications	✓	✕	✓	✓	✕	✕	✕	[46]
4	Smart buildings and intelligent transportations with artificial intelligence and digitalization technology	✓	✕	✕	✕	✕	✕	✕	[47]
5	Machine learning for performance prediction in smart buildings: Photovoltaic self-consumption and life cycle cost optimization	✓	✕	✓	✓	✕	✕	✕	[44]
6	Machine learning and predictive control-based energy management system for smart buildings	✕	✓	✓	✕	✕	✕	✕	[45]
7	Deep learning in healthcare: Opportunities, threats, and challenges in a green smart environment solution for smart buildings and green cities—towards combating COVID-19	✕	✕	✕	✓	✕	✕	✕	[51]
8	Towards automated occupant profile creation in smart buildings: A machine learning-enabled approach for user persona generation	✓	✕	✓	✕	✕	✕	✕	[48]
9	Machine learning-based predictive model for thermal comfort and energy optimization in smart buildings	✓	✕	✓	✓	✕	✕	✕	[49]
10	An overview of machine learning applications for smart buildings.	✓	✕	✓	✕	✕	✕	✕	[53]
11	Application of an AI-based optimal control framework in smart buildings using borehole thermal energy storage combined with wastewater heat recovery	✓	✕	✕	✕	✕	✕	✕	[52]
12	Smart building fire safety design driven by artificial intelligence	✓	✕	✕	✕	✕	✕	✕	[50]
13	Energy-efficient heating control for smart buildings with deep reinforcement learning	✕	✕	✕	✓	✕	✕	✕	[97]
14	Artificial intelligence evolution in smart buildings for energy efficiency	✓	✕	✓	✓	✕	✕	✕	[98]
**Artificial intelligence for energy efficiency in buildings**									
15	Optimizing high-rise buildings for self-sufficiency in energy consumption and food production using artificial intelligence: Case of europoint complex in rotterdam	✓	✕	✕	✓	✕	✕	✕	[99]
16	An artificial intelligence-based method to efficiently bring CFD to building simulation	✓	✕	✕	✕	✕	✕	✕	[100]
17	Overview of computational intelligence for building energy system design	✓	✕	✕	✕	✕	✕	✕	[92]
18	Approximate model predictive building control via machine learning	✕	✕	✓	✓	✕	✕	✕	[101]
19	Heat loss coefficient estimation applied to existing buildings through machine learning models.	✕	✕	✓	✕	✕	✕	✕	[102]
20	Application of machine learning to estimate building energy use intensities	✕	✕	✓	✕	✕	✕	✕	[103]
21	The future role of artificial intelligence (AI) design's integration into architectural and interior design education is to improve efficiency, sustainability, and creativity.	✓	✕	✕	✕	✕	✕	✕	[104]
22	Artificial intelligence for calculating and predicting building carbon emissions: a review.	✓	✕	✕	✕	✕	✕	✕	[91]
23	An integrated artificial intelligence-driven approach to multi-criteria optimization of building energy efficiency and occupants' comfort: A case study	✓	✕	✕	✕	✕	✕	✕	[31]
24	Applications of artificial intelligence for energy efficiency throughout the building lifecycle: An overview.	✓	✕	✕	✕	✕	✕	✕	[88]
25	Intelligent management of industrial building energy saving based on artificial intelligence	✓	✕	✕	✕	✕	✕	✕	[105]
26	Building energy management and forecasting using artificial intelligence: Advance technique.	✓	✕	✕	✕	✕	✕	✕	[106]
27	Early energy performance analysis of smart buildings by consolidated artificial neural network paradigms	✓	✕	✕	✓	✕	✕	✕	[107]
28	AI-powered deep learning for sustainable industry 4.0 and internet of things: enhancing energy management in smart buildings	✓	✓	✕	✓	✕	✕	✕	[108]
29	A systematic literature review on the use of artificial intelligence in energy self-management in smart buildings	✓	✕	✕	✕	✕	✕	✕	[109]
30	Integrated applications of building information modeling and artificial intelligence techniques in the aec/fm industry.	✓	✕	✕	✕	✕	✕	✕	[37]
31	Optimal control of renewable energy in buildings using the machine learning method.	✕	✕	✓	✕	✕	✕	✕	[36]
32	Artificial intelligence for calculating and predicting building carbon emissions: A review	✓	✕	✓	✓	✓	✕	✕	[91]
**Artificial intelligence for thermal comfort in buildings**									
33	Using an ensemble machine learning methodology-bagging to predict occupants' thermal comfort in buildings	✕	✕	✓	✕	✕	✕	✕	[110]
34	Artificial intelligence tools and inverse methods for estimating the thermal diffusivity of building materials	✓	✕	✕	✕	✕	✕	✕	[111]
35	Energy and thermal modelling of an office building to develop an artificial neural networks model.	✓	✕	✕	✕	✕	✓	✕	[112]
36	Field studies of the artificial intelligence model for defining indoor thermal comfort to acknowledge the adaptive aspect.	✓	✕	✕	✕	✕	✓	✕	[113]
37	Adaptive thermal comfort approach to save energy in tropical climate educational building by artificial intelligence	✓	✕	✕	✕	✕	✓	✕	[114]
38	Intelligent building control systems for thermal comfort and energy-efficiency: A systematic review of artificial intelligence-assisted techniques	✓	✕	✕	✕	✕	✕	✕	[86]
39	Comprehensive integration of artificial intelligence in optimizing hvac system operations: a review and future outlook.	✓	✕	✕	✓	✕	✕	✕	[115]
40	Nonlinearity in thermal comfort-based control systems: A systematic review.	✓	✕	✕	✓	✕	✕	✕	[116]
41	Towards various occupants with different thermal comfort requirements: A deep reinforcement learning approach combined with a dynamic pmv model for hvac control in buildings.	✕	✕	✕	✓	✕	✕	✕	[117]
42	Optimizing building heat load prediction using advanced control strategies and artificial intelligence for hvac system.	✓	✕	✕	✕	✕	✕	✕	[118]
43	Enhancing IAQ, thermal comfort, and energy efficiency through an adaptive multi-objective particle swarm optimizer-grey wolf optimization algorithm for smart environmental control.	✓	✕	✕	✕	✕	✓	✕	[119]
44	AI based temperature reduction effect model of fog cooling for human thermal comfort: climate adaptation technology.	✓	✕	✕	✕	✕	✕	✕	[120]
45	Artificial intelligence (AI)-based occupant-centric heating ventilation and air conditioning (HVAC) control system for multi-zone commercial buildings	✓	✕	✕	✕	✕	✕	✕	[121]
**Artificial intelligence for building performance**									
46	Building performance simulation in the brave new world of artificial intelligence and digital twins: a systematic review.	✓	✓	✕	✕	✕	✕	✕	[84]
47	Applications of artificial intelligence enabled systems in buildings for optimized sustainability performance	✓	✕	✕	✕	✕	✕	✕	[122]
48	Optimizing building energy performance predictions: a comparative study of artificial intelligence models	✓	✕	✕	✕	✕	✕	✕	[123]
49	The artificial intelligence reformation of sustainable building design approach: a systematic review on building design optimization methods using surrogate models	✓	✕	✓	✕	✕	✕	✕	[124]
50	Application of artificial intelligence technique in optimization and prediction of the stability of the walls against wind loads in building design	✓	✕	✓	✓	✕	✕	✕	[125]
**Artificial intelligence in green buildings**									
51	Artificial intelligence in green building	✓	✕	✕	✕	✕	✕	✕	[55]
52	Constructing a smart framework for supplying the biogas energy in green buildings using an integration of response surface methodology, artificial intelligence and petri net modelling	✓	✕	✓	✕	✕	✕	✕	[59]
53	Research on sustainability evaluation of green building engineering based on artificial intelligence and energy consumption	✓	✕	✕	✕	✕	✕	✕	[61]
54	Review of artificial intelligence techniques in green/smart buildings	✓	✕	✕	✕	✕	✕	✕	[89]
55	Bim-supported automatic energy performance analysis for green building design using explainable machine learning and multi-objective optimization	✕	✕	✓	✕	✕	✕	✕	[35]
56	Thermal conductivity improvement in a green building with nano insulations using machine learning methods	✕	✕	✓	✕	✕	✕	✕	[67]
57	A machine learning-based two-stage integrated framework for cost reasonableness prediction of green building projects	✕	✕	✓	✕	✕	✕	✕	[62]
58	Application of hybrid machine learning algorithm in multi-objective optimization of green building energy efficiency	✕	✕	✓	✕	✕	✕	✕	[60]
59	Evaluation of waste management and energy saving for sustainable green building through analytic hierarchy process and artificial neural network model	✓	✕	✕	✕	✕	✕	✕	[68]
**Artificial intelligence in zero, net zero, and nearly zero energy buildings**									
60	A comprehensive review on technologies for achieving zero-energy buildings	✓	✕	✕	✕	✕	✕	✕	[81]
61	Net zero energy cost building system design based on artificial intelligence	✓	✕	✕	✕	✕	✕	✕	[33]
62	Prospective research trend analysis on zero-energy building (ZEB): An artificial intelligence approach	✓	✕	✕	✕	✕	✓	✕	[79]
63	Data-driven prediction and optimization toward net-zero and positive-energy buildings: a systematic review.	✓	✕	✓	✕	✕	✕	✕	[80]
64	Energy management in zero-energy building using neural network predictive control.	✓	✕	✕	✕	✕	✓	✕	[72]
65	Leveraging digital twins for zero-energy building ratings in sustainable smart cities: a comprehensive review and novel framework	✓	✕	✕	✕	✕	✕	✕	[2]
66	Optimizing NZEB performance: A review of design strategies and case studies.	✓	✕	✕	✕	✕	✕	✕	[76]
67	ExplainerX: An integrated and explainable AI framework for nearly zero-energy buildings.	✓	✕	✕	✕	✕	✕	✕	[78]
68	Design and accomplishment of ai control strategy with api in nearly zero energy building smart grid.	✓	✕	✕	✕	✕	✕	✕	[77]
69	A hybrid optimization approach for autonomy enhancement of nearly-zero-energy buildings based on battery performance and artificial neural networks	✓	✕	✕	✕	✕	✓	✕	[74]
70	Intelligent optimization framework of near zero energy consumption building performance based on a hybrid machine learning algorithm	✕	✕	✓	✕	✕	✕	✕	[75]
71	Artificial intelligence method for the forecast and separation of total and HVAC loads with application to energy management of smart and NZE homes.	✓	✕	✕	✕	✕	✕	✕	[70]
72	Nearly zero-energy building load forecasts through the competition of four machine learning techniques	✕	✕	✓	✕	✕	✕	✕	[71]
73	An optimal surrogate-model-based approach to support comfortable and nearly zero energy buildings design.	✓	✕	✕	✕	✕	✓	✕	[73]
**Artificial intelligence-driven digital twins in buildings**									
74	Machine learning and artificial intelligence for digital twin to accelerate sustainability in positive energy districts.	✕	✕	✓	✕	✕	✕	✕	[34]
75	Cyber-physical systems improving building energy management: Digital twin and artificial intelligence	✓	✕	✓	✕	✕	✕	✕	[25]
76	Artificial intelligence and a digital twin are effecting building energy management.	✓	✓	✓	✕	✕	✕	✕	[82]
77	AI -powered digital twins and internet of things for smart cities and sustainable building environment	✓	✕	✕	✕	✕	✕	✕	[5]
78	Prediction of an efficient energy-consumption model for existing residential buildings in lebanon using an artificial neural network as a digital twin in the era of climate change.	✓	✕	✕	✕	✕	✓	✕	[29]
79	Digital twin technology for thermal comfort and energy efficiency in buildings: A state-of-the-art and future directions.	✓	✕	✕	✓	✕	✓	✕	[26]
80	A digital twin for energy consumption prediction and thermal comfort monitoring in residential buildings	✓	✓	✕	✕	✕	✕	✕	[28]
81	Improving building energy footprint and asset performance using digital twin technology.	✕	✓	✕	✕	✕	✕	✕	[83]
82	Digital twin with machine learning for predictive monitoring of CO_2_ equivalent from existing buildings.	✓	✓	✓	✕	✕	✕	✕	[27]
83	Digital twins in built environments: An investigation of the characteristics, applications, and challenges	✓	✓	✕	✕	✕	✕	✕	[85]
**Artificial intelligence and generative artificial intelligence for architectural design**									
84	A generative architectural and urban design method through artificial neural networks.	✓	✕	✓	✕	✕	✕	✕	[126]
85	Artificial intelligence applied to conceptual design. A review of its use in architecture.	✓	✕	✓	✓	✕	✕	✕	[127]
86	Generative design of outdoor green spaces based on generative adversarial networks	✓	✕	✓	✓	✕	✕	✕	[128]
87	Learning to generate urban design images from the conditional latent diffusion model.	✓	✕	✕	✓	✓	✕	✕	[129]
88	Artificial intelligence applications in earthquake resistant architectural design: determination of irregular structural systems with deep learning and image AI method	✓	✕	✕	✕	✕	✕	✕	[130]
89	The role of artificial intelligence in architectural design: conversations with designers and researchers.	✓	✕	✓	✕	✕	✕	✕	[131]
90	Visualized co-simulation of adaptive human behavior and dynamic building performance: An agent-based model (ABM) and artificial intelligence (AI) approach for smart architectural design	✓	✕	✓	✕	✕	✕	✕	[132]
91	AI-assisted design: Utilizing artificial intelligence as a generative form-finding tool in architectural design studio teaching	✓	✕	✕	✕	✕	✕	✓	[133]
92	Integrating multimodal generative ai and blockchain for enhancing generative design in the early phase of architectural design process	✓	✕	✕	✕	✕	✕	✓	[134]
93	Generative artificial intelligence and building design: Early photorealistic render visualization of façades using local identity-trained models	✓	✕	✕	✕	✕	✕	✓	[135]
94	Experiments on generative AI-powered parametric modeling and bim for architectural design.	✓	✕	✕	✕	✕	✕	✓	[136]
95	Generative AI for architectural design: A literature review.	✓	✕	✕	✕	✕	✕	✓	[137]
96	Sketch-to-architecture: Generative AI-aided architectural design	✓	✕	✕	✕	✕	✕	✓	[138]
97	Generative AI design for building structures.	✓	✕	✕	✕	✕	✕	✓	[139]
98	Design process with generative AI and thinking methods: Divergence of ideas using the fishbone diagram method	✓	✕	✕	✕	✕	✕	✓	[140]
99	Bibliometric analysis of generative design, algorithmic design, and parametric design in architecture	✓	✕	✕	✕	✕	✕	✕	[141]
100	Can artificial intelligence mark the next architectural revolution? Design exploration in the realm of generative algorithms and search engines.	✓	✕	✕	✕	✕	✕	✓	[142]
101	Prototyping with generative AI.	✓	✕	✕	✕	✕	✕	✓	[143]
102	Generative AI and the history of architecture	✓	✕	✕	✕	✕	✕	✓	[144]
103	Generative vs. non-generative AI: Analyzing the effects of AI on the architectural design process.	✓	✕	✓	✕	✕	✕	✓	[145]
104	Rethinking computer-aided architectural design (CAAD)—From generative algorithms and architectural intelligence to environmental design and ambient intelligence	✓	✕	✓	✕	✕	✕	✓	[146]
105	Building layout generation using site-embedded GAN model	✕	✕	✕	✕	✕	✕	✓	[147]
106	3D building fabrication with geometry and texture coordination via hybrid GAN	✕	✕	✕	✕	✕	✕	✓	[148]
107	Architectural layout generation using a graph-constrained conditional generative adversarial network (GAN)	✕	✕	✕	✕	✕	✕	✓	[149]
108	GAN as a generative architectural plan layout tool: A case study for training DCGAN with Palladian plans and evaluation of DCGAN outputs	✕	✕	✕	✕	✕	✕	✓	[150]
109	Generation of geometric interpolations of building types with deep variational autoencoders	✕	✕	✕	✕	✕	✕	✓	[151]
110	FloorDiffusion: Diffusion model-based conditional floorplan image generation method using parameter-efficient fine-tuning and image inpainting	✕	✕	✕	✕	✕	✕	✓	[152]
111	Research on predicting building façade deterioration in winter cities using diffusion model	✕	✕	✕	✕	✕	✕	✓	[153]
112	Using generative AI Midjourney to enhance divergent and convergent thinking in an architect's creative design process	✕	✕	✕	✕	✕	✕	✓	[154]
113	Text semantics to controllable design: A residential layout generation method based on stable diffusion model	✕	✕	✕	✕	✕	✕	✓	[155]
114	Generating accessible multi-occupancy floor plans with fine-grained control using a diffusion model	✕	✕	✕	✕	✕	✕	✓	[156]
115	Exploring the potential of artificial intelligence as a tool for architectural design: A perception study using Gaudí’s works	✕	✕	✕	✕	✕	✕	✓	[157]
116	Exploration of the intelligent-auxiliary design of architectural space using artificial intelligence model	✓	✕	✕	✓	✕	✕	✕	[158]
117	A machine learning model driven by geometry, material, and structural performance data in the architectural design process	✓	✕	✓	✕	✕	✕	✕	[159]
**Artificial intelligence in the architecture, engineering, and construction industry**									
118	Artificial intelligence in construction engineering and management	✓	✕	✕	✓	✕	✕	✕	[160]
119	Systematic review of application of artificial intelligence tools in architectural, engineering and construction.	✓	✕	✕	✕	✕	✓	✕	[38]
120	Towards human-centered artificial intelligence (AI) in architecture, engineering, and construction (AEC) industry.	✓	✕	✕	✕	✕	✕	✕	[39]
121	Conversational artificial intelligence in the AEC industry: A review of present status, challenges and opportunities	✓	✕	✕	✕	✕	✓	✕	[40]
122	Application of artificial intelligence in sustainable construction.	✓	✕	✕	✕	✕	✕	✕	[41]

**Table 2:** Contributions of artificial intelligence to environmental sustainability indicators in smart buildings.

**No.**	**Energy efficiency and demand reduction**	**Renewable energy integration and optimization**	**Carbon footprint and emissions monitoring**	**Water efficiency and resource management**	**Indoor environmental quality and thermal comfort**	**Predictive maintenance and building lifecycle optimization**	**Reference**
1	✓	✕	✕	✕	✕	✕	[42]
2	✓	✓	✕	✕	✕	✕	[43]
3	✓	✕	✕	✓	✕	✓	[46]
4	✓	✓	✓	✕	✕	✕	[47]
5	✓	✓	✕	✕	✕	✕	[44]
6	✓	✓	✕	✕	✕	✓	[45]
7	✓	✓	✕	✕	✕	✕	[51]
8	✓	✕	✕	✕	✓	✕	[48]
9	✓	✕	✕	✕	✓	✓	[49]
10	✓	✓	✓	✕	✕	✕	[53]
11	✓	✓	✓	✕	✕	✕	[52]
12	✕	✕	✕	✕	✕	✓	[50]
13	✓	✕	✕	✕	✕	✕	[97]
14	✓	✓	✓	✕	✕	✕	[98]

**Table 3:** Contributions of artificial intelligence to environmental sustainability indicators in green buildings.

**No.**	**Energy performance and passive design optimization**	**Renewable energy integration and net-zero goals**	**Carbon footprint reduction and climate adaptation**	**Sustainable water and resource management**	**Indoor environmental quality and well-being**	**Waste reduction, circular economy, and sustainable materials**	**Reference**
1	✓	✕	✓	✕	✓	✓	[55]
2	✓	✕	✕	✕	✕	✓	[59]
3	✓	✓	✕	✓	✕	✓	[61]
4	✓	✕	✓	✓	✕	✕	[89]
5	✓	✕	✓	✕	✕	✕	[35]
6	✓	✕	✕	✕	✕	✓	[67]
7	✓	✕	✕	✕	✕	✓	[62]
8	✓	✕	✓	✕	✕	✓	[60]
9	✓	✓	✕	✕	✕	✓	[68]

**Table 4:** **Contributions of** artificial intelligence to environmental sustainability in zero, net-zero, and nearly-zero energy buildings.

**No.**	**Energy efficiency and demand reduction**	**Renewable energy generation and storage optimization**	**Carbon footprint reduction and net-zero carbon strategies**	**Water efficiency and resource management**	**Smart indoor environmental quality and thermal comfort**	**Optimized predictive maintenance and lifecycle management**	**Reference**
1	✓	✓	✓	✕	✓	✓	[81]
2	✓	✓	✕	✕	✕	✕	[33]
3	✓	✓	✓	✓	✕	✕	[79]
4	✓	✓	✓	✕	✓	✓	[80]
5	✓	✓	✕	✕	✕	✓	[72]
6	✓	✓	✓	✕	✓	✓	[76]
7	✓	✕	✕	✕	✕	✕	[78]
8	✓	✕	✕	✕	✕	✕	[77]
9	✓	✓	✕	✕	✕	✓	[74]
10	✓	✓	✓	✕	✓	✓	[75]
11	✓	✕	✕	✕	✕	✓	[70]
12	✓	✕	✕	✕	✕	✓	[71]
13	✓	✓	✕	✕	✓	✓	[73]

In complement to the tabular and thematic mapping presented in the first part of the results, the synthesis deepens the analysis by categorizing the studies into four major areas of advancement. Each area corresponds to one or more aspects of the research questions and collectively highlights how AI and DT technologies are operationalized across SGZEB typologies. This structured synthesis identifies the core innovations within each category and uncovers cross-cutting patterns, emerging trends, and evolving practices that position AI and DTs as key drivers of environmental sustainability across building domains and scales. This section provides a comprehensive and integrated view of the state-of-the-art landscape by bridging the detailed mapping of applications with broader thematic insights.

### 5.1 Comprehensive analysis of artificial intelligence applications in building systems and environmental sustainability indicators

This subsection presents a detailed analysis of the first part of this section, starting with Table 1, which includes 5.1.1, 5.1.2. It summarizes nine thematic areas and explores their interconnections in the broader context of AI applications across building typologies, domains, and technologies. Table 1 highlights how various AI subdomains are applied and interrelated, offering insights into their collective impact on smart building design, energy efficiency, thermal comfort, building performance, green buildings, ZEBs, DTs, generative architectural design, and key aspects of AI applications in the AEC industry. Subsection 5.1.3 focuses on established environmental indicators across SGZEBs, highlighting how AI and DT technologies enhance their implementation and performance.

In Subsection 5.1.4, which involves Table 2, Table 3, Table 4, the focus shifts to how AI contributes to the environmental indicators related to the three building typologies. These tables systematically map the integration of AI in addressing specific environmental metrics across SGZEBs. The organization of findings provides a holistic understanding of the role of AI in sustainable smart building practices, providing insights into how environmental indicators shape the design and performance of these building typologies. Through this tabulated and thematic analysis, the comparative component becomes evident, allowing for the identification of patterns, trends, and contrasts across the three typologies. This comparison highlights the differing ways AI contributes to environmental goals and points to areas that may require further exploration. The dual approach examines how AI-driven innovations align with sustainable practices and uncovers synergies between technology, environment, and architectural design. Overall, these tables form a comprehensive perspective on how AI is transforming the built environment and enhancing the environmental performance of SGZEBs.

#### 5.1.1 Tabulated thematic analysis of artificial intelligence models in building typologies, domains, and technologies

Table 1 presents a systematic mapping of 109 peer-reviewed studies that apply AI in the context of buildings. Each row indicates whether a particular AI model or subdomain is applied in the given study, allowing for a quantitative and thematic overview of how AI is being implemented across different areas of building research. Table 1 provides a detailed overview of how AI is being integrated into various building typologies, domains, and technologies, reflected in nine key themes: (1) AI in smart buildings, (2) AI for energy efficiency, (3) AI for thermal comfort, (4) AI for building performance, (5) AI in green buildings, (6) AI in ZEBs, (7) AI and DTs in buildings, (8) AI and generative AI in architectural design, and (9) AI in the AEC industry. It functions as a meta-review tool, supporting both trend analysis and gap identification by highlighting which AI technologies are most prevalent and which areas remain underexplored or require integration.

Across these themes, it is clear that AI is transforming the built environment in several important ways. In the realm of smart buildings, AI is increasingly linked with IoT to optimize building management systems, enabling real-time monitoring, predictive control, and improved decision-making. A major focus lies in energy optimization, where ML- and DL-driven models enhance efficiency, reduce consumption, and integrate renewables. Another strong thread is occupant-centered applications, which enhance comfort, safety, and well-being. Beyond energy and comfort, AI is also advancing safety and risk management.

When it comes to energy efficiency, AI's role is primarily driven by ML and DL techniques, which enable the development of data-driven models for predicting energy usage patterns and optimizing building performance. These algorithms are instrumental in detecting inefficiencies, forecasting demand, and automating the operation of HVAC systems and lighting. ML and DL facilitate intelligent control systems that dynamically adapt to user behavior and environmental conditions, resulting in reduced energy consumption, cost savings, and enhanced sustainability across various building types.

Thermal comfort is another key area where AI, including DL and NLP, enhances occupant well-being while supporting energy optimization. DL models are used to analyze complex environmental data and predict indoor comfort conditions with high precision. At the same time, NLP techniques enable systems to interpret user feedback and natural language inputs about thermal preferences. These AI techniques allow smart HVAC systems to dynamically adjust temperature, humidity, and airflow conditions in real-time, ensuring personalized comfort while maintaining energy efficiency.

In connection with building performance, AI aids in predicting how buildings will function over their lifespan, both in terms of energy use and structural integrity. AI tools simulate various operational scenarios, allowing for the optimization of building design and the identification of potential performance issues before they arise. This predictive capability is key to improving the operational efficiency of buildings both after construction and during the design phase. Technologies, such as ML, DT, CPS, and IoT, are increasingly interconnected with building performance simulation.

AI is also playing a significant role in advancing green buildings. AI, including ML, contributes to reducing the environmental impact of buildings by optimizing energy use and resource management. This includes the integration of renewable energy sources, efficient waste management systems, and sustainable material choices. Through data analysis, AI helps make green buildings more efficient, cost-effective, and aligned with sustainability goals.

The push for ZEBs, NZEBs, and nZEBs is another area where AI is making a major impact. AI is used to balance energy consumption with energy generation, enabling buildings to produce as much energy as they consume. AI-driven strategies support the integration of renewable energy sources and the optimization of energy storage and distribution systems. ML techniques are widely applied for load forecasting and system optimization, while NLP has emerged as a tool for interpreting user feedback or documentation to fine-tune energy strategies. These applications are critical to achieving net-zero energy targets and advancing global sustainability efforts.

The concept of DT is transforming how buildings are managed and optimized. DTs, as digital replicas of physical buildings powered by AI, enable real-time monitoring, simulation, and optimization of building performance. These virtual models identify issues before they arise, leading to more efficient building operations and better decision-making. ML plays a crucial role in predicting energy consumption patterns and enhancing system efficiency. The integration of the AIoT allows for seamless communication between devices, contributing to smarter building operations and data collection. In addition, NLP is used to analyze building-related documents or feedback, providing valuable insights for more efficient resource management and better decision-making. These technologies lead to more proactive building operations and significant improvements in sustainability.

Furthermore, AI is significantly advancing architectural design beyond the capabilities of traditional methods. Through ML and DL, architects can analyze complex datasets, optimize spatial layouts, and enhance building performance predictions at early design stages. While Generative AI is enabling the automated exploration of innovative, functional, and aesthetic design solutions, broader AI techniques support design decision-making by processing spatial, environmental, and user data. This integration of AI, spanning from predictive analytics to creative generation, is opening up new possibilities for both efficiency and creativity in the architectural design process.

Lastly, AI is making a significant impact across the AEC industry. From enhancing project management and construction processes to improving material selection and safety, AI technologies are streamlining operations and reducing inefficiencies. NLP, as noted in two studies, is being applied to interpret construction documents, extract actionable insights, and improve communication across stakeholders. More broadly, AI tools are automating routine tasks, predicting delays, optimizing workflows, and enhancing on-site safety through predictive hazard detection and risk mitigation strategies. These applications are transforming the AEC industry by driving smarter, more data-informed decision-making.

Worth noting, among the various AI models and subdomains examined across the 122 studies, CV appears to be the least commonly utilized, with only a few instances identified across the thematic landscape. This limited representation can be attributed to several interconnected factors. First, many CV applications depend on large volumes of visual data, such as images, video streams, or sensor-based spatial inputs, which are not always readily available or feasible to collect in typical building environments, particularly in existing or retrofitted structures. Moreover, the integration of visual monitoring technologies into occupied spaces often raises significant privacy and ethical concerns, especially when monitoring occupants or user behavior in real time. These concerns have likely constrained the broader deployment of CV tools in real-world smart and sustainable building contexts.

In addition, the thematic focus of most studies tends to revolve around areas such as energy efficiency, predictive maintenance, thermal comfort, or renewable integration—domains where ML and DL techniques, often based on numerical, time-series, or categorical data, are more directly applicable. In contrast, CV methods are typically associated with more specialized use cases, such as construction monitoring, visual defect detection, or occupancy estimation, which may not align as directly with the core environmental indicators that dominate the reviewed literature. Moreover, the technical complexity and resource requirements of deploying CV systems also present barriers. Implementing such systems often demands specialized hardware (e.g., cameras, light detection and ranging [LiDAR]), robust computational infrastructure, and advanced image processing capabilities, elements that may be beyond the scope of many research initiatives. As a result, while CV holds clear potential in specific aspects of the built environment, its practical integration into AI-driven sustainability frameworks remains limited and context-dependent.

In conclusion, across these nine themes, AI is driving a transformation in the built environment. From enhancing energy efficiency and occupant comfort to enabling smarter, more sustainable design and construction practices, AI is at the heart of the future of SGZEB technologies. These developments highlight AI's ability to make buildings more intelligent, efficient, and environmentally friendly, paving the way for a more sustainable and innovative built environment.

#### 5.1.2 Thematic convergence of AI applications in the built environment

The previous subsection addresses the individual themes that define the role of AI in building typologies, domains, and technologies, analyzing their unique contributions. However, the real power of AI in this context lies not just in isolated applications but in the way these elements are integrated to enhance overall building outcomes. This subsection delves into the synergies between these AI-driven innovations, highlighting how their interconnections create smarter, more efficient, and sustainable building environments. Understanding these relationships offers valuable insight into how AI can transform the built environment as an integrated whole, thereby driving progress across multiple dimensions of design, energy efficiency, comfort, and performance**.**

The relationship between AI in smart buildings and energy efficiency is particularly synergistic. Smart buildings rely on AI to collect and analyze data from building systems, such as HVAC, lighting, space usage, and security, optimizing them in real-time for both performance and efficiency. ML algorithms are used to predict energy consumption based on environmental data and occupancy patterns. The feedback from energy-efficient strategies in smart buildings enhances the AI's learning capabilities, which allows for continuous optimization over time. This feedback loop ensures that energy management becomes more accurate, reducing overall consumption while maintaining comfort levels.

AI's role in thermal comfort is closely tied to energy efficiency. AI can dynamically adjust heating or cooling systems to maintain comfort while optimizing energy usage by utilizing real-time data from sensors measuring temperature, humidity, air quality, and occupancy. As AI systems become more precise in understanding human comfort preferences and environmental conditions, they can better balance energy savings with occupant satisfaction. For instance, AI can optimize HVAC systems to lower energy consumption while keeping spaces at comfortable temperatures, thus addressing both thermal comfort and energy efficiency simultaneously.

AI for building performance and green buildings are interconnected through the optimization of building systems and the reduction of environmental impact. AI is used to simulate and predict a building's performance across various operational scenarios, such as energy use, structural integrity, and resource consumption, helping to identify areas for improvement. These predictions, combined with AI's ability to model green building systems, allow for the fine-tuning of energy systems, water usage, waste management, and materials in ways that maximize sustainability. As buildings become more performance-driven and energy-efficient, the potential for achieving green building standards such as leadership in energy and environmental design (LEED) or building research establishment environmental assessment method (BREEAM) increases, which demonstrates how AI drives sustainable architectural design.

NZEB concept directly benefits from AI's ability to optimize energy efficiency and renewable energy integration and optimization. Buildings can be designed to achieve a state where they produce as much energy as they consume by using AI algorithms to balance energy inputs (from renewable sources) with internal consumption. AI's predictive capabilities and real-time data analysis assist in forecasting energy generation patterns, managing energy storage, and integrating systems that align building performance with environmental goals. The shift towards NZEB is closely tied to AI's ability to optimize energy flows and system efficiencies, while also contributing to these broader goals by reducing reliance on nonrenewable energy sources.

AI-powered DTs play an important role in enhancing building performance. These virtual replicas of physical buildings allow for real-time monitoring, simulation, and optimization. The integration of AI into DTs enables the creation of highly accurate models that simulate how buildings will behave over time under different conditions. AI can predict maintenance needs, optimize systems, and improve building lifespan by integrating performance data into these virtual models. In this sense, AI enhances building performance through simulations and predictive maintenance and helps make adjustments that are both cost-effective and sustainable.

The connection between AI in architectural design and smart buildings is rooted in the potential of AI to inform and optimize design decisions before construction even begins. AI algorithms can be used to simulate building designs, considering various factors such as energy use, occupant behavior, and environmental impact. These AI-driven designs can then be integrated with smart building systems to ensure that the building operates efficiently from the moment it is completed. Furthermore, AI-generated designs can make buildings more adaptable to future needs, integrating features such as smart sensors and automated systems that enhance overall building intelligence.

The integration of AI into the AEC industry and smart buildings is mutually reinforcing. In construction, AI technologies are used for project planning, scheduling, resource management, and quality control. These technologies ensure that smart building features are implemented effectively and with precision. For example, AI tools are used to optimize the construction process, detect potential design flaws, and improve collaboration between stakeholders, which in turn results in more efficient smart building designs. Moreover, the data collected during the construction phase can be fed into building management systems to allow AI to continuously monitor and optimize performance.

AI's integration into the AEC industry influences both thermal comfort and green buildings by driving more sustainable and efficient building designs. ML models can optimize energy flows, design passive solar systems, and incorporate natural ventilation strategies in ways that enhance both comfort and sustainability. For instance, an AI-based model might suggest the most energy-efficient placement of windows to maximize natural lighting and reduce heating or cooling costs, thus ensuring that the building remains comfortable and energy-efficient. This approach reduces the reliance on mechanical systems and minimizes environmental impact, which aligns well with green building principles.

Taken as a whole, these themes form a network where each contributes to and enhances the others. AI's ability to optimize energy systems, predict building performance, and improve sustainability is a key thread that runs through all of these areas. With new advances in AI, the relationship between themes such as energy efficiency, thermal comfort, and building performance becomes increasingly integrated, allowing for a more holistic approach to building design, operation, and sustainability. In this context, AI does not just serve isolated functions; rather, it enables a more interconnected, dynamic, and responsive built environment where performance, comfort, and sustainability are continuously optimized through data-driven insights.

By bringing together these diverse elements, AI can establish a comprehensive framework that transforms the built environment into a more intelligent, adaptable, and future-ready system. This interconnectedness demonstrates how AI's subdomains, including those in relation to data analytics and predictive modeling, play critical roles across various building functions, enabling advanced technologies in each area to complement and enhance one another seamlessly.

#### 5.1.3 Environmental indicators enhanced by artificial intelligence and digital twins across smart, green, and zero-energy buildings

To explore the intersection of AI, particularly ML and DL, and DTs with environmentally sustainable built environments in the subsequent subsections, this study utilizes well-established environmental indicators that are recognized across academic and industry research. These indicators, which are used to assess the environmental performance of buildings, form the foundation for evaluating the impacts of AI and DT technologies on energy efficiency, carbon emissions reduction, resource management, and overall sustainability in SGZEBs. The application of these indicators is crucial for understanding how advanced technologies can enhance environmental outcomes in these buildings, making them a critical lens through which the contributions of AI and DTs are assessed.

This study situates these indicators within a broader context, ensuring that the reviewed literature validates them and highlights the advancements that AI and DT technologies bring to the field. The following synthesis presents key studies that report on how these indicators have been operationalized and improved across SGZEB typologies.

Smart buildings represent a dynamic typology where digital intelligence is tightly integrated into the design, operation, and performance of the built environment. Several studies focus on defining and measuring the concept of smartness in buildings. Dakheel et al. [161] explore the concept of smart buildings, highlighting their main features, functions, and technologies, while also developing a set of nine groups of representative performance indicators. Their results emphasize the need for quantified guidelines to enhance energy performance and technological innovation in smart buildings. Similarly, Ghansah et al. [162] investigate indicators for measuring the smartness of buildings in the construction industry. Using survey data from 227 respondents, they find that awareness of smart building technologies (SBTs) is moderately high in the Ghanaian construction industry and develop a blueprint guidance model to support policymakers and improve building performance. Alanne [163] introduces the learning ability index (LAI) to quantify the learning capacity of buildings. Applying the index to three case studies, the author demonstrates that LAI provides a flexible and illustrative measure of building intelligence, monitoring data-driven processes, and supporting strategies for higher levels of smartness.

Other studies examine environmental impacts and sustainability performance. Lagarde et al. [164] assess the environmental impact of integrating connected devices in residential buildings using life cycle assessment with uncertainty analysis. Their results show that while connected devices improve environmental performance compared to the original building, full refurbishment remains the most effective strategy across almost all indicators. They also emphasize the importance of measurement campaigns to more accurately quantify energy gains. Koller et al. [165] explore the environmental dimensions of smart buildings through a literature review, building analysis, and expert interviews. The authors demonstrate that standardized definitions, enhanced data availability, and stakeholder collaboration are essential for achieving measurable ecological benefits. They provide case studies illustrating the practical impacts on building sustainability.

A separate group of studies addresses smart building readiness and indoor environmental quality. Delavar et al. [166] examine the smart readiness indicator (SRI), aiming to evaluate buildings’ readiness to support energy-efficient and adaptive functionalities. Their findings reveal rapid growth in SRI research, primarily focused on energy efficiency, and show that the SRI can be applied beyond individual buildings to neighborhoods and districts. They identify six understudied research areas necessary for advancing the evolution of smart buildings, including the applicability of SRI across various contexts and its integration with other standards. Aldakheel et al. [167] focus on AI techniques for evaluating indoor environmental quality in smart buildings. The authors demonstrate that smart real-time monitoring and intelligent ventilation strategies optimize occupant comfort and energy efficiency, highlighting the role of ML and DL in selecting appropriate indicators and measurement technologies for smart indoor environments.

Regarding green buildings, several studies focus on developing, assessing, and prioritizing indicators to measure the sustainability performance of green buildings across environmental, social, and economic dimensions. Abdel-Basset et al. [168] aim to establish a framework for evaluating sustainable green building indicators in developing countries under uncertain conditions. Using a multi-criteria decision-making (MCDM) method combined with the Delphi method and analytical hierarchy process (AHP), the authors assess and prioritize the dimensions and indicators of green building design. Their results show that water efficiency is the most significant dimension (weight = 0.330), while energy efficiency is the least significant (weight = 0.100) for green buildings in developing countries. The study concludes with practical administrative implications for applying sustainable development strategies in green buildings, emphasizing the need for adaptation to local characteristics and resource availability.

Focusing on ecological indicators for green building construction, Liu and Lin [169] quantify regional differences in ecological performance. Using a slack-based data envelopment analysis approach and a panel dataset from 1995 to 2012, the authors reveal that roughly half of China's provinces have the potential to improve ecological performance by more than 60%, with developed areas outperforming developing areas. In addition, they identify the 11th five-year plan as a turning point, where national green building policies significantly enhance ecological performance, highlighting the strong influence of policy and planning on sustainable building practices.

Marotta et al. [170] examine whether green buildings can serve as an indicator of broader sustainable development. Using data from Eurostat and green building directories across 27 European Union countries (2010–2019), the authors apply linear regression analysis and confirm the environmental Kuznets curve hypothesis: economic growth in developed countries is associated with environmental improvements. The study demonstrates that the variance in green building implementation correlates strongly with both gross domestic product per capita (*p* = 0.0004, *R*^2^ = 0.8475) and greenhouse gas emissions (*p* = 0.0002, *R*^2^ = 0.8825), supporting the idea that green buildings are an effective measure of sustainable development and emphasizing the importance of policies such as tax incentives to encourage their adoption.

Li et al. [171] develop key performance indicators (KPIs) for operational monitoring of green buildings, seeking a practical, efficient alternative to comprehensive evaluation standards that are time- and labor-intensive. The authors establish a library of 27 KPIs encompassing outdoor and indoor environmental quality, HVAC systems, renewable energy, total resource consumption, and occupant behavior. Using the Delphi method and the specific, measurable, achievable, relevant, and time-bound (SMART) principle, the KPIs are validated through two Chinese case studies, demonstrating that the framework enables long-term monitoring while being more practical and systematic than conventional approaches, thereby reducing evaluation time and costs.

Braulio-Gonzalo et al. [172] focus on how green building rating systems (GBRS) address sustainability and life cycle frameworks in residential buildings. Analyzing 387 indicators across eight GBRS, they classify them by sustainability dimension, information module, and construction stage. Their results indicate that the environmental dimension is most emphasized, while social and economic dimensions require more attention. Furthermore, most indicators focus on the product and construction stages (A1–A5) rather than the early design or operational stages, suggesting that a more holistic, lifecycle-spanning approach is necessary. Building on this, the study by Sartori et al. [173] focuses on developing a schematic environmental impact assessment (EIA) framework for building design, integrating LCA and GBRS. The authors compare LCA and GBRS methodologies, analyze the inclusion of LCA parameters in GBRS, and review LCA software compatible with GBRS requirements. The findings suggest that the most suitable EIA approach varies according to the stage of the design life cycle. Combining LCA's quantitative analysis with GBRS's qualitative criteria enhances transparency, supports better-informed design decisions, and improves environmental performance assessment, especially when linked to graphical outputs and three-dimensional modeling.

ZEBs, NZEBs, nZEBs, and PEBs represent the forefront of sustainable design, emphasizing energy self-sufficiency, carbon neutrality, and long-term environmental resilience. Research on these building typologies emphasizes the need for robust performance indicators and holistic assessments to guide design, construction, and operation. Key studies have explored various approaches to evaluate energy efficiency, renewable energy use, and environmental sustainability. Indicators such as self-consumption, self-production, loss-of-load probability, and coverage rate are proposed to measure a building's energy performance, although their practical application can be challenging [174]. Complementary metrics, including the overall renewable energy fraction (OREF), extend conventional indicators by accounting for both on-site and off-site renewable energy, emphasizing independence from fossil fuels and highlighting the benefits of self-consumption over exported energy [175]. Early selection of performance thresholds is recommended to guide design decisions, with seven key thresholds identified to balance trade-offs and overcome societal and technical barriers in NZEB development [176].

Environmental aspects in nZEB design have also been investigated. Sensitivity analyses indicate that maximizing renewable energy generation, particularly through photovoltaic systems, often reduces life-cycle environmental impacts more effectively than increasing insulation, especially in Mediterranean and continental climates [177]. However, life cycle assessments indicate that the carbon footprint of renewable energy technologies, such as photovoltaic (PV) panels, depends on the energy mix of the country where they are produced, which can influence overall greenhouse gas emissions [178]. Together, these studies suggest that achieving NZEB/nZEB goals requires a multi-criteria approach, combining energy performance indicators with life-cycle environmental analyses. Using both types of assessments ensures that designs not only meet net-zero energy targets but also minimize broader environmental impacts.

Furthermore aiming to facilitate the transition from PEBs to positive energy communities (PECs), the study by Cai and Gou [179] introduces a set of KPIs to evaluate site planning and energy autonomy potential. Through geographic analyses and simulations based on data from 81 PEBs, the study evaluates factors such as energy surplus, spatial coverage, and shared energy dynamics under different photovoltaic (PV) installation scenarios and spatial ranges. The results indicate that establishing PECs from existing PEBs is feasible, with optimal community boundaries typically falling between 150 and 250 m. The proposed KPIs offer practical guidance for site selection, community planning, and policymaking, supporting the creation of sustainable and energy-positive urban developments.

Overall, these studies provide robust validation for the environmental indicators used in this review, demonstrating their relevance and applicability across various building typologies. They reveal how the role of AI and DT technologies is not only compatible with established sustainability metrics but also enhances their implementation, accuracy, and real-world impact. The reviewed literature showcases an evolving paradigm in which intelligent systems drive measurable environmental improvements by integrating these technologies with domain-specific indicators—laying the groundwork for more adaptive, efficient, and ecologically integrated built environments.

#### 5.1.4 Tabulated thematic mapping and cross-typology comparative analysis of environmental indicators and artificial intelligence in smart, green, and zero-energy buildings

This subsection presents a detailed thematic and comparative analysis of environmental indicators driven by AI across three distinct building typologies: SGZEBs. The analysis begins with Table 2, Table 3, Table 4, which systematically map peer-reviewed studies on AI integration within these typologies and synthesizes their insights according to these key tailored indicators. These tables provide a visual representation of how AI contributes to sustainability across building typologies. The comparative approach adopted identifies patterns and trends across these typologies, contrasting the role of AI in supporting different indicators. The analysis highlights knowledge gaps, such as underrepresented indicators in specific typologies, and synthesizes key insights into the broader role of AI in advancing environmental goals in the built environment.

Table 2 systematically maps 14 peer-reviewed studies on the integration of AI in smart buildings, specifically evaluating their contribution to the identified indicators. It is organized by citation and the categorical presence of these indicators. Each row reflects whether a given study addresses specific sustainability dimensions, providing a thematic overview of how AI applications align with environmental goals in the built environment. Table 2 facilitates a visual analysis of prevalent research foci, revealing areas that require further investigation in AI-enabled smart building design and operation.

AI applications in smart buildings overwhelmingly concentrate on energy efficiency and demand reduction (Table 2), which appears consistently across nearly all studies. Predictive maintenance and renewable energy integration also emerge as recurring themes, whereas water efficiency and carbon footprint monitoring remain largely underexplored. This distribution suggests that research on AI in smart buildings is still primarily oriented towards operational optimization, with sustainability dimensions such as resource management and emissions reduction representing notable gaps for future investigation.

Table 3 systematically maps nine peer-reviewed studies as an illustrative subset on the integration of AI in the context of green buildings. Similarly organized by citation and categorical presence, it highlights how AI supports sustainability goals in this typology. It provides a thematic overview of prevalent research trends and highlights underexplored areas in AI-enabled green building design and operation.

Research on AI in green buildings most frequently emphasizes energy performance and passive design optimization (Table 3), which is a consistent focus across all reviewed studies. Waste reduction and circular strategies also recur, often in conjunction with more specific applications. In contrast, areas such as renewable energy integration, carbon footprint reduction, and particularly sustainable water management are far less explored. This imbalance indicates that while AI is being applied to optimize building performance and material efficiency, its potential to drive climate resilience, net-zero transitions, and holistic resource management in green buildings remains underdeveloped.

Table 4 systematically maps 13 peer-reviewed studies on the integration of AI within zero-energy, net-zero-energy, and nearly zero-energy buildings. Likewise, it uses the same categorical mapping approach to identify which sustainability indicators are addressed. The table provides a comparative overview of AI's role in advancing energy neutrality and sustainability, while also highlighting indicators that remain relatively underrepresented.

Table 4 presents AI applications in zero, net-zero, and nearly-zero energy buildings, which most prominently address energy efficiency, renewable energy generation and storage optimization, and predictive maintenance, reflecting a strong operational and systems-integration focus. A number of studies also link AI with carbon reduction and net-zero carbon strategies, underscoring its central role in achieving climate targets. By contrast, water efficiency and, to a lesser extent, indoor environmental quality remain underexplored, suggesting that resource management and occupant-centered outcomes are secondary priorities in this domain. Overall, the research landscape reveals that AI is being leveraged primarily to ensure energy neutrality and long-term building performance, while opportunities for a more holistic sustainability approach are not yet fully realized.

The indicators featured in the three comparative tables represent a shared framework of environmental concerns—energy, water, carbon, indoor environmental quality, and lifecycle optimization—that are broadly relevant across all three typologies. However, their implementation is tailored to the core objectives of each building typology. For example, while energy efficiency is a common goal, smart buildings emphasize real-time demand response, green buildings focus on passive performance, and ZEBs prioritize achieving net-zero status through energy balancing.

These shared indicators offer a consistent lens through which AI contributions to environmental goals can be assessed, while still acknowledging the specific design logic and functional priorities of each typology. In smart buildings, AI is primarily applied to real-time system optimization and environmental quality control. In green buildings, AI supports passive design enhancement, climate resilience, and long-term material planning. In zero-energy typologies, AI enables seamless integration of on-site renewables with energy demand, supporting energy-positive or carbon-neutral operation. Although the same thematic indicators are used to structure the comparison across typologies, their implementation and significance vary depending on each building type's sustainability strategy. This shared framework enables structured cross-typology analysis while honoring each typology's distinct trajectory.

The synthesis of the three typology-specific tables reveals both convergence and divergence in how AI supports environmental performance (Table 5). Smart buildings prioritize automated control and operational efficiency. Green buildings focus on lifecycle intelligence, natural system optimization, and resource circularity. ZEBs emphasize energy neutrality through AI-optimized energy generation and storage.

**Table 5:** Comparative analysis of artificial intelligence-driven sustainability indicators across smart, green and zero-energy buildings.

**Sustainability indicators**	**Smart buildings**	**Green buildings**	**Zero-energy buildings**
Energy efficiency and demand reduction	AI automates HVAC, lighting, and energy use through real-time data and adaptive controls.	AI optimizes passive design strategies, including natural ventilation, shading, and insulation.	AI balances energy demand using predictive modeling and smart grids.
Renewable energy integration and storage optimization	AI integrates renewables with smart grids, optimizes battery storage, and enables demand response.	AI forecasts renewable energy availability and enhances hybrid energy system integration.	AI ensures on-site renewables meet energy needs, manages energy storage, and interacts with the grid.
Carbon footprint reduction and net-zero strategies	AI tracks emissions, optimizes electrification, and suggests carbon reduction pathways.	AI selects low-carbon materials, models building lifecycle emissions, and supports carbon-neutral design.	AI-driven carbon accounting ensures net-zero operations and offsets unavoidable emissions.
Water efficiency and resource management	AI detects leaks, optimizes irrigation, and predicts water demand.	AI improves rainwater harvesting, greywater recycling, and sustainable plumbing design.	AI integrates water-energy nexus for efficiency and monitors real-time consumption.
Indoor environmental quality and thermal comfort	AI-based HVAC, air quality monitoring, and adaptive lighting optimize occupant well-being.	AI enhances passive thermal comfort strategies and daylighting optimization.	AI balances thermal comfort with energy neutrality while ensuring air quality.
Predictive maintenance and lifecycle optimization	AI-powered DTs and fault detection reduce energy and resource waste.	AI predicts material degradation, lifecycle impacts, and resource reuse.	AI ensures long-term building performance while maintaining zero-energy targets.

Merging the strengths of the three typologies offers the most comprehensive path forward. These findings suggest that no single AI-driven approach is universally adequate. Instead, the most promising pathway lies in hybrid models that integrate automation, passive strategies, and net-zero frameworks. This hybridization forms the foundation for a unified, typology-aware, cross-domain AI sustainability framework, one that supports intelligent, resilient, and environmentally optimized building design. AI can unlock a built environment that is adaptive, high-performing, and holistically sustainable by merging the strengths of SGZEB approaches.

### 5.2 Thematic synthesis of advancements and applications in smart, green, and zero-energy buildings: artificial intelligence and digital twins for environmentally sustainable smart built environment

This subsection analyzes and synthesizes the selected studies to provide a deeper, thematic understanding of how AI, ML, DL, and DT technologies are enhancing the environmental outcomes of SGZEBs. It moves beyond the tabular comparative analysis by offering an integrated examination of key trends, innovations, and implementation areas. Specifically, it explores how AI and DTs enable smart buildings to achieve greater efficiency, performance, thermal comfort, and intelligent control; how green buildings leverage AI for enhanced energy efficiency, sustainable design, and waste management; how AI supports the optimization, management, and realization of zero-energy, net-zero-energy, and nearly-zero-energy buildings; and finally, how AI-driven DTs are applied across building systems to support the development of sustainable smart built environments. This thematic synthesis offers a more comprehensive account of the transformative role of AI and DTs, connecting technological advancements and applications to broader environmental objectives.

#### 5.2.1 Smart buildings: leveraging artificial intelligence and machine learning for enhanced efficiency, performance, thermal comfort, and control

In recent years, AI has emerged as a key driver in the evolution of smart buildings. As cities around the world strive to enhance sustainability, energy efficiency, and occupant well-being, AI-powered smart buildings are at the forefront of this transformation, enabling more intelligent, adaptive, and environmentally conscious urban spaces. The reviewed studies provide a comprehensive overview of how AI and ML are transforming the environmental aspects of smart buildings. Covering diverse applications, they illustrate a multifaceted approach that supports the development of more efficient, adaptive, and user-centered smart building environments.

Sleem and Elhenawy [42] provide a comprehensive overview of the emerging field of AIoT and its applications in smart buildings. AIoT, which merges AI algorithms with data generated by IoT, enables real-time monitoring, automation, and intelligent decision-making. The study highlights the potential of AIoT to enhance smart building operations by reducing energy consumption and operational costs, improving occupant comfort and productivity, and strengthening safety and security systems. It also outlines key challenges in implementing AIoT in smart buildings, including issues of data privacy, security, interoperability, and the demand for specialized technical expertise. Overall, it positions AIoT as a transformative enabler in the development of smarter and more efficient building systems. The study lays the foundation for subsequent research by highlighting how real-time data from IoT devices can optimize various building systems, setting the stage for AI-driven solutions to enhance building performance across multiple domains.

In an integrative approach, Pan and Zhou [47] examine how AI and digitalization technologies are transforming smart buildings and intelligent transportation systems. Their study emphasizes the role of integrated digital solutions in advancing carbon neutrality through renewable energy integration, energy efficiency, and smart mobility. The discussion highlights smart buildings as key components within e-mobility and energy-sharing frameworks, illustrating the growing convergence of digital infrastructure across built and transport environments. AI and digitalization are further portrayed as crucial enablers of sustainability and grid independence in urban systems. This perspective aligns with Wang et al. [43], who discuss the use of DTs for energy optimization, and emphasizes the interconnected nature of smart cities, where building and transportation systems must be optimized together.

In this line of thinking, Sen et al. [45] explore the critical role of smart buildings within the broader context of smart city development. Emphasizing the importance of sustainable urban infrastructure, the authors provide an overview of how smart buildings integrate renewable energy sources, such as solar photovoltaics, mini wind turbines, and biomass, with energy storage and smart grid technologies. The focus is placed on the development of predictive control-based energy management systems that leverage ML for enhanced performance. A particular emphasis is given to the application of ML techniques for forecasting variable parameters related to both energy generation and consumption. These forecasts are then used to optimize energy management through predictive control, aiming to balance efficiency, occupant comfort, and sustainability. The study positions smart buildings as fundamental to the realization of smart cities and highlights the importance of integrating ML and control systems to improve operational efficiency and reduce energy consumption. This aligns with the findings of Gupta et al. [97] on RL for heating control and extends the discussion on predictive models by linking real-time data inputs with long-term energy optimization. It also relates to several studies that focus on improving the adaptability and efficiency of building systems.

Exploring strategies for achieving carbon peak and net-zero emissions in smart buildings, Wang et al. [43] integrate AI and DT technologies into renewable energy management. The authors introduce a modified differential evolution (DE) algorithm combined with RF regression to forecast renewable energy generation and optimize power distribution in a smart building microgrid. They aim to balance economic efficiency with environmental resilience, reducing both operational costs and emissions by formulating a nonlinear multi-objective optimization model. The study demonstrates the flexibility of the proposed AI approach under different power exchange scenarios and compares its performance against particle swarm optimization (PSO) using real-time data. A key innovation is the incorporation of DT—virtual models of physical microgrid systems—to simulate and optimize the behavior of renewable energy sources such as solar and wind. This integration enhances predictive capabilities and operational control, positioning DTs as a powerful tool in transitioning towards low-carbon, energy-efficient smart building ecosystems. The study's outcome aligns with the findings on ML's role in optimizing photovoltaic energy consumption [180] and thermal storage solutions [52]. These approaches support the transition to net-zero emissions and contribute to reducing the environmental footprint of smart buildings.

Farzaneh et al. [98] examine how AI is transforming smart buildings to achieve higher energy efficiency. The authors highlight the integration of sensors, big data, and AI technologies in building management systems (BMS) and demand response programs (DRPs) to improve energy control, automation, and system reliability. They categorize AI applications across energy use prediction, occupant comfort, building design, and maintenance. The findings align with Alanne and Sierla [53], who emphasize comprehensive adaptability in smart buildings through autonomous AI agents and DTs. They also complement Anik et al. [48], who demonstrate the use of ML to automate occupant profiling, further enhancing the efficiency and user-centeredness of AI-driven building management systems.

In their investigation of how ML can be leveraged to improve PV self-consumption and optimize life cycle costs in smart buildings, Amini Toosi et al. [180] address the challenge of modeling energy storage systems (ESS), which is often complex and time-consuming. In this context, the authors evaluate 24 ML models as surrogate tools for analyzing PV performance. Among them, Gaussian process regression, neural networks, support vector machines (SVM), and Ensemble Trees emerged as top performers for accurate and efficient predictions. They further explore how short-term thermal energy storage (TES), when paired with electric heat pumps, can greatly improve PV self-consumption. A key outcome shows that optimizing TES size using ML-based life cycle cost analysis can yield up to 7.1% savings over a 30-year building lifespan. This study identifies effective ML models for PV prediction and demonstrates their potential in enabling smarter, cost-effective, and energy-efficient building systems. This study complements Wang et al.'s focus on renewable energy integration and aligns with the ongoing discussions about optimizing renewable energy systems [52] to reduce reliance on traditional power grids. It also feeds into the broader dialogue on AI's role in managing energy systems more efficiently.

Anik et al. [48] introduce an ML-based approach to automate occupant profiles that personalize building management systems, an essential step towards human-centered design and energy efficiency. The study directly contributes to enhancing building management systems and energy optimization efforts, particularly in terms of personalized control over heating, lighting, and ventilation. Using the Residential Energy Consumption Dataset, six ML algorithms (e.g., RF, SVM, AdaBoost) were tested to classify and predict 16 occupant characteristics, including thermal comfort, age, and cooling preferences. The models achieved over 90% accuracy for certain features such as age group and cooling equipment usage. The study demonstrates that ML can effectively streamline persona smart creation, supporting smarter, more personalized building design. This focus on occupant modeling provides a foundation for Boutahri and Tilioua [49], who extend ML-driven personalization into real-time occupancy prediction and thermal comfort optimization, thus bridging static user profiling with dynamic energy management in smart buildings.

The integration of occupant data into energy management systems is discussed further by Boutahri and Tilioua [49], where an ML-based predictive model optimizes thermal comfort based on real-time occupancy data and energy efficiency in smart buildings. Using data from sensor-equipped Raspberry Pi devices, the study evaluates four ML algorithms—SVM, ANN, RF, and extreme gradient boosting (XGBoost)—to forecast thermal comfort (via predicted mean vote [PMV]) and optimize HVAC energy consumption. Among the tested models, RF and XGBoost demonstrate the highest accuracy (up to 96.7%), notably outperforming SVM. The findings highlight the strong potential of ML algorithms, particularly ensemble methods, to improve both user comfort and energy efficiency in intelligent building systems. This study further develops the ideas from Anik et al. [48] by incorporating real-time data from occupants to dynamically adjust building systems for optimal energy use and predict thermal comfort needs. It also connects with Sen et al. [45] on predictive control, reinforcing the importance of using AI to anticipate building needs and enhance energy efficiency.

Alshamrani et al. [52] propose an AI-enhanced optimal control framework designed to improve energy efficiency and sustainability in smart residential buildings. The framework integrates borehole thermal energy storage (BTES) with wastewater heat recovery, heat pumps, and a smart ventilation system. Using TRNSYS and MATLAB, the authors develop and simulate an intelligent energy system that reclaims heat from wastewater and radiator return water and preconditions ventilation air, enhancing energy reuse and reducing carbon emissions. At the core of the system is an AI-assisted control strategy, specifically an ANN, which optimizes energy storage and usage in real time. This smart integration achieves significant improvements over conventional ventilation systems, showing a reduction in energy costs by $41.5 MWh^−1^, a total cost saving of over $10,000, and a CO_2_ emissions reduction of 1.7 kg MWh^−1^. This study also reveals that performance can be further optimized through strategies like adjusting mass flow rates and borehole depth, contributing to both environmental and economic benefits. This work ties into the broader sustainability goals outlined by Wang et al. [43] and Amini Toosi et al. [44], where energy systems are optimized for carbon neutrality. It also links AI with practical energy management solutions in real-world applications.

Gupta et al. [97] propose a deep reinforcement learning (DRL)-based heating controller to enhance thermal comfort and reduce energy consumption in smart buildings. Using real-world temperature data in simulation experiments, the DRL controller demonstrates a 15–30% improvement in thermal comfort and a 5–12% reduction in energy costs compared to conventional thermostat systems. The study also compares centralized and decentralized DRL-based control in multi-building scenarios, finding that decentralized control performs better as the number of buildings and their temperature preferences vary. It highlights the potential of DRL for more adaptive and energy-efficient building management systems. The study relates to the predictive control models discussed by Sen et al. [45] and Maurya et al. [51], which emphasize the importance of data-driven solutions for optimizing heating systems and energy use in buildings.

Baduge et al. [46] investigate how AI, ML, and DL are being applied across the full lifecycle of buildings within the framework of Construction 4.0. The study focuses on the use of these technologies in diverse areas, including architectural and structural design, material optimization, offsite manufacturing, construction management, safety monitoring, smart operations, and building maintenance. A notable strength of the study is its holistic approach, examining how AI and smart vision systems support buildings from initial concept to end-of-life, with an emphasis on life cycle analysis and the circular economy. The study positions AI as a transformative force in creating more intelligent, sustainable, and efficient buildings throughout their lifecycle. This work aligns with earlier works on AI in building management systems by showcasing its application in optimizing materials, structural design, and lifecycle analysis. It links well with studies on smart operations and the need for continuous AI-based monitoring and learning (e.g., Ref. [45,52]).

Alanne and Sierla [53] examine the integration of ML and AI in smart buildings, emphasizing the role of these technologies in improving energy efficiency, adaptability, and resilience in the face of unpredictable operational changes, especially those related to climate change. The authors take a comprehensive approach, with a focus on autonomous AI agents that can make independent decisions for energy management across a building's life cycle. They highlight the use of DTs as training environments to enhance the learning processes in building. The study concludes that the greatest potential for improving energy efficiency lies in incorporating AI-driven adaptability solutions within HVAC control systems and electricity market participation. This study supports the idea that AI can autonomously optimize energy systems in real time, an approach that complements predictive control models and reinforcement learning discussed earlier (e.g., Ref. [45,[49], [97]]), which focus on improving energy management through AI.

Maurya et al. [51] discuss the role of DL in fostering sustainable, green smart buildings and cities. The authors present renewable energy as a key alternative to coal, focusing on smart buildings as part of the future of clean energy solutions. The study links AI applications to the broader theme of reducing carbon footprints and supporting green cities, aligning with research that examines AI's role in energy optimization, renewable energy integration, and achieving carbon neutrality [43,180]. It emphasizes how AI can help reduce energy consumption and promote sustainability in smart buildings.

Zeng and Huang [50] introduce an AI-driven approach to building fire safety design through the Intelligent Fire Engineering Tool (IFET), which leverages large datasets from high-fidelity fire simulations. The AI system captures spatiotemporal patterns of fire development, enabling fast and accurate prediction of detector and sprinkler response times under dynamic conditions. It facilitates performance-based fire safety evaluations for complex architectural spaces and can identify critical design thresholds in seconds. The tool aims to support more adaptive, responsive, and efficient fire safety design in smart buildings, with potential for continuous learning and future expansion to broader fire scenarios. This work complements other studies focused on optimizing energy and comfort (e.g., Ref. [49,97]).

Overall, the reviewed studies demonstrate the innovative potential of AI and ML in improving the environmental outcomes of smart buildings. They showcase how these technologies are applied to optimize energy consumption, integrate renewable energy sources, enhance occupant comfort, and improve safety. Each study builds on, relates to, or expands upon the other ones, progressively broadening the scope of AI applications and deepening our understanding of how AI addresses the complex challenges of sustainability in smart buildings. Together, the studies form a cohesive narrative that highlights both the practical applications and conceptual advancements of AI in building technologies. They contribute to the development of smarter, more adaptive, and more sustainable built environments by connecting and building upon each other's findings.

#### 5.2.2 Green buildings

AI is rapidly emerging as a transformative force in advancing green building design, performance, and overall project management. As the AEC industry continues to prioritize sustainability and environmental responsibility, AI-powered solutions are revolutionizing the design, construction, and operation of green buildings. AI applications in green buildings offer vast potential for improving environmental outcomes. Its integration into building systems enables innovative design processes, smarter energy efficiency solutions, waste reduction strategies, and carbon emission monitoring—critical components for achieving carbon neutrality. In addition, AI is increasingly applied in cost estimation, risk assessment, and overall project evaluation, supporting more informed decision-making, optimized resource allocation, and reduced operational and financial uncertainties. The reviewed studies provide a comprehensive overview of how AI and ML are reshaping green buildings, highlighting key advancements and applications across various domains.

##### 5.2.2.1 Artificial intelligence applications in green building design: performance, prediction, optimization, and sustainability

Recent research highlights the diverse ways AI is being integrated into green building design, ranging from predictive modeling and compliance support to optimization frameworks and critical reflections on the role of creativity. The reviewed studies demonstrate AI's role not only in improving efficiency and sustainability but also in reshaping professional practice and decision-making in the architecture and construction sectors.

Bura and Bharati [94] investigate the application of AI in green building design, emphasizing its potential to streamline compliance with sustainability rating systems. The authors highlight how AI facilitates faster and more reliable decision-making in areas such as energy efficiency, water management, ventilation, and daylighting. The study demonstrates that AI tools can support architects, engineers, and designers in optimizing building performance by enhancing the likelihood of achieving green building certification. It concludes that AI provides a broad scope of applications and significant advantages in improving productivity, communication, and sustainability outcomes in the green building sector. This study relates to Sari et al. [181], as both explore AI methods for enhancing the efficiency and predictive capabilities of green building design.

Omar and Al-Boridi [96] examine how AI can improve green building construction for environmental sustainability, focusing on reducing carbon emissions and energy consumption. Their work employs predictive analytics and ML algorithms, including SVM and GAs, to optimize construction decisions, concrete mix strength, and energy use. The findings show that AI models can achieve prediction accuracies above 95%, with genetic algorithms (GA) models predicting CO_2_ emissions with an *R*^2^ of 0.95. In addition, k-fold cross-validation confirmed the robustness of these models, demonstrating that AI can significantly lower operational costs, improve efficiency, and reduce greenhouse gas emissions during construction. These findings extend the practical applications discussed by Bura and Bharati [94] by providing concrete examples of AI in operational green building processes.

In examining the broader implications of AI adoption in sustainable building design, Jain and Babu [95] focus on its impact on architectural practice and human creativity. The study identifies potential risks, including reduced innovation, diminished personal expression, and the simplification of professional roles, resulting from reliance on AI-generated solutions. Despite these challenges, the authors argue that AI can serve as a valuable tool for augmenting human decision-making, provided it is applied judiciously. Their findings advocate for a human-centered approach in AI-assisted design, ensuring that technology enhances, rather than undermines, the cognitive and artistic contributions of architects. This critical perspective highlights an often-overlooked dimension in research on AI in green buildings, encouraging other studies to consider not only technical performance and efficiency but also the preservation of creativity, professional judgment, and the cultural and intellectual richness of architectural practice.

Sari et al. [181] focus on developing ML models to predict green building design performance, aiming to accelerate the design process while maintaining sustainability standards. The study evaluates criteria such as energy efficiency, indoor environmental quality, water efficiency, and site planning. Among the tested models, the combination of ANNs with an IF-ELSE algorithm produces the most accurate predictions, achieving a mean square error of 1.3. These results suggest that ML-based predictive models can effectively support designers in creating optimized green buildings more efficiently, thereby reducing the time and complexity traditionally associated with integrating sustainable design. This study relates to Bura and Bharati [94] because both emphasize AI/ML for design efficiency, but it focuses specifically on predictive modeling rather than broad design compliance or sustainability assessment.

Shen and Pan [35] propose a framework that combines BIM with XML and multi-objective optimization to predict and optimize energy performance in green building design. The framework integrates DesignBuilder simulation, Bayesian Optimization-LightGBM (BO-LGBM), and SHAP (Shapley additive explanation) to provide accurate predictions of energy performance. It also uses the AGE-MOEA algorithm for multi-objective optimization, minimizing energy consumption, CO_2_ emissions, and indoor thermal discomfort. The results showed that the BO-LGBM model achieves a prediction accuracy with an *R*^2^ value greater than 93.4% and a mean absolute percentage error smaller than 2.13%. The optimization process yields a 13.43% improvement in energy performance, and considering uncertainty further enhances the results by approximately 4%. This approach enhances transparency and efficiency in green building design by providing interpretable predictions and optimizing key building performance factors. This study complements both Sari et al. [181] and Bura and Bharati [94], as all three emphasize AI/ML support for design efficiency, though Shen and Pan [35] advance the discussion by combining predictive modeling with interpretable optimization tools for greater transparency and multi-objective performance improvement.

Liu et al. [58] propose a BIM-enabled hybrid ML framework to address the challenge of balancing multiple objectives in green building design. The approach achieves optimized design parameters that simultaneously reduce life cycle carbon emissions, lower costs, and enhance thermal comfort by integrating BIM-DesignBuilder simulations with RF prediction, Grey Wolf Optimization, and NSGA-II, The case study shows reductions of 16.6% in carbon emissions, 2% in economic cost, and an 18.3% improvement in comfort, highlighting the framework's value in supporting reliable multi-objective optimization for sustainable building design. This study complements Shen and Pan [35], as both employ BIM with AI-driven optimization to enhance energy performance, while Liu et al. [58] extend the scope by explicitly integrating cost and comfort trade-offs into the optimization process.

The study by Mahmood et al. [56] investigated how to optimize green building design by applying ML and DL techniques to the ASHARE-884 dataset, with preprocessing methods such as *Z*-Score normalization and label encoding to improve model performance. A range of algorithms are tested, including ML models like RF, DT, and EGB, and DL models such as GNN, LSTM, and RNN, with evaluation based on metrics like accuracy, precision, recall, and F1-score. The findings show that GNN and LSTM outperform conventional DL techniques, offering greater efficiency and accuracy in enhancing environmental practices. Accelerating the design process and enhancing decision-making, these models help reduce environmental impacts, optimize resources, and improve occupant comfort, underscoring AI's crucial role in shaping more sustainable green building design practices. This work complements Liu et al. [58] by further demonstrating how advanced AI models can optimize resource use and occupant comfort, extending optimization strategies beyond energy and cost to encompass more holistic sustainability goals.

On the whole, these works indicate that AI applications in green buildings are evolving from predictive tools to comprehensive decision-support and optimization systems, addressing energy, cost, comfort, and sustainability in increasingly integrated ways, while also raising essential questions about creativity and human-centered design. The convergence of technical precision, operational efficiency, multi-objective optimization, and critical reflection underscores AI's transformative yet complex role in advancing sustainable architectural design.

##### 5.2.2.2 Leveraging artificial intelligence for advancing energy efficiency, waste management, thermal comfort, and sustainability

Recent research highlights how predictive modeling, ML, and multi-objective optimization frameworks can improve operational performance, reduce energy use and carbon emissions, and support sustainable decision-making across building design, materials, and occupant management.

Xiang et al. [61] propose an AI-based energy management model (AI-EMM) designed to optimize energy consumption in green buildings. The AI-EMM utilizes infrared communication systems and smart user identification subsystems to adapt energy use based on the internal and external environments, aiming to enhance user comfort, safety, and energy efficiency. The model incorporates long short-term memory (LSTM) techniques to predict energy needs, thereby enhancing the efficiency of HVAC systems. The study's experimental results demonstrate a high performance ratio of 94.3%, a 15.7% reduction in energy consumption, a prediction accuracy of 97.1%, and an energy management level of 95.7%. These findings demonstrate that AI can play a crucial role in enhancing energy management in green buildings, aligning with environmental objectives. This study complements Mahmood et al. [56] by demonstrating predictive energy management using AI, where both studies emphasize accurate forecasting to improve HVAC efficiency and reduce energy consumption.

Shahsavar et al. [59] introduce a smart framework for supplying biogas energy in green buildings by integrating response surface methodology (RSM), AI, and Petri net modeling. The study focuses on addressing energy supply and waste management in green buildings, particularly in relation to SDGs. The framework employs various AI techniques, including random tree, RF, ANN, and adaptive-network-based fuzzy inference system (ANFIS), to predict accumulated biogas production (ABP). Among these, ANFIS achieves the highest accuracy, with a correlation coefficient of 0.99. The study also integrates a dynamic control system using Petri Net modeling to optimize the biogas production process. This novel approach emphasizes the synergy between energy supply, waste management, and sustainability in green buildings. This work aligns with Lu et al. [68] in addressing sustainable waste management and energy efficiency, and both studies extend the discussion on AI's role from optimizing waste strategy to predicting and dynamically controlling energy recovery processes. It also connects to Feng et al. [57], as integrating renewable energy sources complements AI-driven energy efficiency strategies.

Lu et al. [68] propose a framework for evaluating waste management and energy-saving strategies in green buildings, integrating the analytic hierarchy process (AHP) with ANN. The study focuses on construction and demolition waste, aiming to reduce waste sent to landfills and lower the use of energy and resources. Their approach evaluates various waste management strategies, including incineration, composting, and landfilling, taking into account environmental, social, and economic factors. The study finds that composting performs best when environmental aspects are prioritized, while incineration and landfilling are more favorable when considering social and economic criteria. This study builds on Shahsavar et al. [59] by exploring decision-making strategies in waste management, complementing their focus on dynamic biogas production. It also links to Xiang et al. [61] in emphasizing AI-supported optimization to improve overall building efficiency and sustainability outcomes.

Ghalandari et al. [67] propose the use of ML models to optimize the thermal conductivity and energy efficiency of green buildings through the application of nano-insulation. The authors focus on the impact of different insulation thicknesses and configurations on energy consumption, utilizing ML methods such as SVM, Gaussian process regression, and decision trees. Their results demonstrate that the decision tree model offers the best performance for predicting thermal conductivity with an accuracy above 99%. It reveals that buildings with Nano insulation save up to 40% more energy compared to conventional insulation materials. Moreover, the energy savings per unit area and the reduction in CO_2_ emissions range between 290 and 293 kg m^−3^, depending on various factors such as weather conditions and insulation specifications. This study complements Xiang et al. [61] and Mahmood et al. [56,182], as all emphasize AI/ML-driven improvements in energy efficiency, and it also connects to Zhu et al. [60] by demonstrating how optimization of materials and design parameters can feed into multi-objective building performance frameworks.

Addressing the problem of energy inefficiency caused by gaps in occupant behavior and energy management, Mahmood et al. [56] introduce an ML approach based on active learning for predictive modeling in green buildings. Their work develops models capable of predicting heating and cooling demands with high accuracy, using a wide range of regressors including RF, DT, GB, XGBoost, CatBoost, LGBM, KNN, and LR. The proposed CBR-AL model achieves exceptional predictive performance, with *R*^2^ values of 0.9975 for cooling and 0.9883 for heating. Beyond its technical accuracy, the model demonstrates significant potential for reducing energy consumption, improving operational efficiency, lowering carbon footprints, and generating cost savings. This predictive framework sets a benchmark for next-generation energy management systems in green buildings. This study extends Xiang et al. [61] by demonstrating broader predictive capabilities for both cooling and heating, and it complements Feng et al. [57] by offering a technical foundation for achieving projected reductions in energy use and emissions through AI.

Zhu et al. [60] present a multi-objective optimization framework for green building design that integrates BIM-DB, Bayesian-RF (Bayesian-RF), and non-dominated sorting genetic algorithm III (NSGA-III). This framework aims to optimize energy efficiency, reduce emissions, enhance cost-effectiveness, and improve thermal comfort by accurately predicting building performance across these factors. The study shows that BIM-DB efficiently generates building data through simulation and orthogonal tests. The Bayesian-RF method significantly improves prediction accuracy, achieving a mean squared error (MSE) below 0.08 and an *R*^2^ above 0.85 for all three prediction objectives. Furthermore, the Bayesian-RF-NSGA-III optimization algorithm reduces energy consumption by 7.68%, carbon emissions by 6.48%, and cost by 1.77%, while also improving overall thermal comfort. These results demonstrate the framework's effectiveness in reducing resource consumption and enhancing comfort while optimizing multiple objectives in green building design. This framework complements Ghalandari et al. [67] by integrating material and design optimization into a predictive and multi-objective platform, and it aligns with Xiang et al. [61] and Mahmood et al. [56] by using AI for predictive energy efficiency and comfort optimization.

Feng et al. [57] investigate how AI can enhance energy management in green buildings by enabling precise forecasting, advanced environmental analysis, and the integration of renewable energy. The results reveal that AI applications can reduce energy consumption by about 8% and CO_2_ emissions by 19% in typical mid-size office buildings by 2050 compared to conventional approaches. Moreover, when combined with energy efficiency policies and low-emission energy production, reductions of up to 40% in energy use and 90% in CO_2_ emissions are projected. The study offers a systematic framework for quantifying AI's energy and carbon-saving potential across building types and climates, providing evidence of its long-term value in achieving sustainability goals. This study is complementary to Xiang et al. [61], Mahmood et al. [56], and Shahsavar et al. [59], as all explore AI for energy efficiency, predictive modeling, and integration with renewable or recovered energy to achieve broader sustainability goals.

In summary, these studies reveal that AI applications in green buildings are evolving from isolated predictive tools to integrated optimization systems that simultaneously enhance energy efficiency, manage waste, improve thermal comfort, and reduce environmental impact. AI enables more sustainable, efficient, and adaptable building practices by linking predictive accuracy with multi-objective decision-making, while also reinforcing the importance of balancing technological performance with occupant needs and environmental goals.

##### 5.2.2.3 Artificial intelligence-driven cost estimation and risk management: enhancing financial accuracy, risk mitigation, and sustainable decision-making

Recent advances in AI are revolutionizing the planning, execution, and management of green building projects. AI applications in this domain enhance the accuracy of cost estimation and provide robust tools for risk assessment, supporting more informed decision-making and sustainable resource allocation throughout the construction lifecycle.

Exploring cost estimation and control in sustainable construction, Zhang [65] introduces the AI-driven comprehensive cost dynamics model (AICD-CDM) to address the complexity of green building projects. The framework integrates multiple ML techniques, including linear regression (LR), ANN, RF, XGBoost, light gradient boosting (LGBoost), and natural gradient boosting (NGBoost), to provide both point predictions and probabilistic forecasts for cost management. Findings demonstrate that the model effectively captures nonlinear relationships among diverse cost-influencing factors, offering enhanced accuracy, adaptability, and computational efficiency. The study demonstrates that the AICD-CDM framework can significantly enhance resource allocation and cost optimization, providing decision-makers with a powerful tool for sustainable project management. This study complements Alshboul et al. [63], who also explore ML approaches for green building cost prediction, by introducing a broader multi-algorithmic framework with probabilistic forecasting capabilities.

In addressing safety considerations, Xu [64] examines fire risk assessment in green intelligent buildings using AI. Leveraging IoT data and expert input, a deep neural network model is developed to predict and assess fire risks. The model is continuously trained and refined, enabling more precise risk predictions for individual building units. Results highlight the potential of AI to integrate real-time data and expert knowledge, providing robust early-warning systems and supporting proactive fire risk management in smart green buildings. This work relates to Zhu et al. [66], as both studies utilize AI for risk assessment in green buildings. Specifically, Xu [64] emphasizes fire safety, while Zhu et al. [66] develop a broader, multi-risk predictive framework.

Focusing on risk management, Zhu et al. [66] propose a hybrid ML approach combining the fuzzy analytic hierarchy process (FAHP), multilayer perceptron neural networks (MLPNNs), and PSO to quantify and predict risks in green building projects. Through structured input from 30 experts, ten risk categories are prioritized, with economic, market, and functional risks identified as the most critical. The model forecasts the impact of the top five risks on project cost, time, quality, and scope, achieving RMSE values between 0.06 and 0.09 and *R*^2^ values up to 0.95. These results indicate the framework's strong predictive capability and its utility for actionable, data-driven risk management in sustainable construction. This study expands on concepts similar to Xu [64] by applying AI for multi-risk prediction beyond fire safety, and it provides a methodological complement to Zhang [65] by quantifying risk impacts that can inform cost and project management decisions.

Addressing cost prediction from a machine learning perspective, Alshboul et al. [63] develop models for forecasting construction costs in green buildings, which present unique challenges due to new technologies and limited stakeholder experience. Using XGBOOST, deep neural networks (DNN), and RF, the study evaluates model performance across soft and hard cost attributes. XGBOOST achieved the highest accuracy of 0.96, followed by DNN at 0.91 and RF at 0.87. The findings demonstrate that AI-based models can provide reliable benchmarks for construction costs, supporting informed decision-making and enhancing automation in green building project management. This study complements Zhang [65], which also addresses AI-based cost estimation, but Zhang extends the approach with a multi-algorithmic framework and probabilistic forecasting for enhanced uncertainty management.

Wu et al. [62] propose a two-stage integrated ML framework for predicting cost reasonableness in green building projects (GBPs). Their approach uses principal component analysis (PCA) integrated with an SVM algorithm for cost prediction and least squares SVM (LSSVM) for determining the cost deviation range. The results, based on 126 project samples, demonstrate that the PCA-SVM model outperforms traditional models, such as SVM and multiple regression analysis, with significantly lower prediction errors. Only 17% of the projects deviated beyond the reasonable cost range. This framework addresses dimensionality challenges and ensures accurate, project-specific cost predictions, supporting sustainable investment in green buildings. This study complements Zhang [65] and Alshboul et al. [63], as all three focus on AI-driven cost estimation. Wu et al. [62] advance the discussion by refining prediction accuracy and tackling dimensionality challenges that others only partially address.

In summary, these studies demonstrate that AI can significantly enhance both the financial and operational management of green buildings. Decision-makers are better equipped to predict costs, manage uncertainties, and mitigate project risks by integrating ML techniques, probabilistic forecasting, and hybrid risk assessment frameworks, thereby advancing the sustainability and efficiency of green building development.

#### 5.2.3 Harnessing artificial intelligence for zero-energy, net-zero-energy, nearly-zero-energy, and positive energy building optimization, real-time control, and transparency

The global surge in energy demand, particularly from the building sector, has intensified the urgency to rethink how building structures are designed, operated, and integrated into broader energy ecosystems. In response, ZEBs, nZEBs, and NZEBs have emerged as key solutions for advancing environmental goals. These building paradigms aim to drastically reduce carbon emissions by balancing or minimizing energy consumption through a blend of energy-efficient designs, renewable energy sources, and advanced management systems. Central to accelerating the realization of these sustainable buildings is the integration of AI, which offers new pathways for optimizing energy performance, forecasting consumption patterns, managing renewable energy flows, and enhancing occupant comfort, all with a level of precision and adaptability previously unattainable. Recent research reflects a growing convergence between AI techniques, such as ML, DL, optimization algorithms, and predictive control systems, and the objectives of ZEBs, nZEBs, and NZEBs constructions.

##### 5.2.3.1 Zero-energy, nearly-zero, and positive energy buildings

The pursuit of zero-energy, nearly-zero, and positive-energy Buildings has intensified in recent years, with AI emerging as a transformative enabler for energy efficiency, renewable integration, and intelligent building management. Wang et al. [81] review the latest advancements in ZEBs, focusing on the growing role of AI to improve energy efficiency. The study highlights three main technological areas: energy-efficient measures (EEMs), renewable energy technologies (RETs), and building energy management systems (BEMS). It emphasizes how EEMs reduce energy demand by enhancing building design, using phase change materials, optimizing HVAC systems, and influencing occupant behavior. It also highlights how renewable sources, such as solar, wind, biomass, and geothermal energy, can be integrated through distributed energy systems. Lastly, it underscores the role of BEMS in managing energy use, detecting faults, and optimizing performance, all while leveraging AI to further improve system efficiency. This work provides a foundation for subsequent studies by demonstrating how AI-driven technologies can integrate energy management and renewable energy, a perspective that is further developed and applied in the works of Yao et al. [183], Megahed et al. [72], and Rocha et al. [33]**.** The focus on the role of AI and BEMS builds upon previous research by introducing a technology-driven dimension to renewable energy integration in ZEBs.

Examining the evolving trajectory of ZEB research, Jin and Bae [79] apply AI and NLP to analyze public research and development grant data. The study provides a detailed analysis of trends within ZEB research, focusing on the entire energy continuum, which encompasses energy supply, demand, distribution, and realization within architectural frameworks. It highlights emerging areas of interest, theoretical gaps, and provides practical recommendations for practitioners and policymakers. It presents both academic insights and practical guidance for the implementation of sustainable strategies in the development of ZEBs. Their use of AI and NLP offers a novel perspective on understanding ZEB research trends, contributing to the academic foundation by providing a data-driven exploration of energy supply, demand, and distribution. This study provides a contextual foundation that complements the application-oriented research of Megahed et al. [72] and Rocha et al. [33], reinforcing the growing role of AI in energy management and guiding future technological interventions in ZEBs.

A novel energy management technique for ZEBs using neural network predictive control (NNPC) is proposed by Megahed et al. [72]. This technique combines two methodologies: neural networks and model predictive control, to optimize energy usage in ZEBs. The key features of NNPC include its real-time operation, ability to connect to the Internet, simple controls, and disturbance reduction. Notably, the system is designed to learn from human behavior, making it more adaptive and efficient. Furthermore, the study introduces a forecasting technique using an ANN to predict renewable energy sources, specifically wind and photovoltaic, to maximize energy utilization without relying on the electrical grid. This study was conducted on a building with a hybrid system and energy storage units, using data from wind and solar measurements over seven months, focusing on a high-energy consumption day. It connects to several key studies in the ZEB field, particularly in the areas of energy management and renewable energy integration. It builds on and complements the work by Wang et al. [81] regarding the integration of renewable energy sources in ZEBs.

Rocha et al. [33] introduce a solution to energy planning in buildings by introducing the concept of nZEcB, buildings with zero or nearly zero annual energy costs. The study employs a range of AI techniques, including bidirectional LSTM, ordinary least squares linear regression, K-means, Pearson's correlation, decision tree, and binary gravitational search algorithm, to design an optimal distributed generation system. This system incorporates renewable energy sources such as wind and photovoltaic, along with a battery bank and an automated capacitor bank for power factor compensation. A case study on a real public building showed that the distributed generation system produced 2.805 GWh annually, which met 160.5% of the building's electrical demand and nearly eliminated energy costs. Despite an excess production of energy, which could not be fully exported due to lower feed-in tariffs, the system proved to be cost-effective with a payback period of 6.79 years. This study extends the work of Megahed et al. [72] by applying AI techniques to optimize distributed generation systems in ZEBs, offering a more comprehensive, AI-driven solution. It advances the understanding of energy management in ZEBs, particularly by incorporating renewable energy sources with AI-based optimization techniques. It also supports the findings of Wang et al. [81], who identified AI and BEMS as key components for enhancing energy efficiency and integrating renewable energy in ZEBs. Furthermore, it corroborates the insights from Jin and Bae [79], reinforcing the growing role of AI in energy management in ZEBs.

Focusing on data-driven approaches for achieving net-zero and positive-energy buildings (PEBs), Mousavi et al. [80] explore how ML, AI, and building modeling simulations can predict energy production and optimize building systems to achieve PEB goals. The authors highlight key factors, including occupant comfort, building efficiency, economic benefits, and clean energy provision, as critical to achieving PEB targets. They categorize data-driven techniques used in PEBs, including renewable energy supply prediction, optimizing building envelope design, and improving comfort control with IoT. They outline a framework for applying these techniques, focusing on reducing energy demand, enhancing energy efficiency, and enabling effective energy management in various building types. Their approach to optimizing renewable energy supply and demand, as well as reducing energy consumption through building envelope design, aligns with the objectives explored by Rocha et al. [33] regarding the use of AI for optimizing energy management in buildings. In addition, this study reinforces the importance of ML and AI for achieving efficient and effective PEB and net-zero building outcomes, complementing Jin and Bae [79], who highlight the growing role of AI in shaping ZEB research trends and future directions.

These advances demonstrate how AI-driven approaches are shifting buildings towards adaptive, self-sufficient systems that meet energy targets and strengthen the foundations for sustainable and resilient built environments. Importantly, they highlight the transition from isolated energy-efficient measures to integrated, data-driven frameworks that align with broader climate goals and the future of smart urban ecosystems.

##### 5.2.3.2 Net-zero-energy buildings

NZEBs are increasingly leveraging AI and data-driven strategies to optimize energy performance, integrate renewable energy sources, and enhance occupant comfort and building autonomy. Focusing on their development and optimization, Ibrahim et al. [76] explore design strategies, technological innovations, and their impact on energy efficiency. The authors highlight the role of AI in enhancing NZEB performance, particularly in predictive energy analytics, intelligent HVAC systems, and real-time energy management. They also address significant barriers to NZEB implementation, such as high costs, regulatory limitations, and inadequate stakeholder participation. They suggest region-specific solutions, such as integrating renewable energy systems and optimizing building envelopes, to overcome the challenges of diverse climates and varying regulatory frameworks. The study advocates for enhanced cooperation and tailored approaches to promote the adoption of NZEB, offering valuable insights for researchers, policymakers, and industry stakeholders seeking to promote sustainable building practices. This study builds upon the work of Wang et al. [81] and Rocha et al. [33] by expanding the role of AI in optimizing energy performance in NZEBs. The focus on renewable energy integration and optimizing building envelopes in regional contexts complements the work of Megahed et al. [72] and Mousavi et al. [80], who also investigate strategies for integrating renewable energy sources and improving energy performance through data-driven approaches.

An ExplainerX is proposed by Kermiche et al. [78] as an integrated XAI framework designed to improve the prediction of energy usage in NZEBs. This framework addresses common shortcomings in current AI solutions, such as the lack of transparency in data and results, issues with model drift, and the use of disparate tools during model development. ExplainerX streamlines the prediction process by providing transparency at each stage, ensuring both performance and interpretability. The framework incorporates components of the CRISP-DM methodology, providing detailed explanations of decision-making processes. A case study using real datasets from the European Union Improvement Project demonstrates the practical application of ExplainerX, showcasing its potential to enhance energy management in NZEBs. This study builds upon the work of Wang et al. [81] and Megahed et al. [72] by addressing the transparency and interpretability issues commonly associated with AI models in the context of ZEBs. Their focus on XAI solutions complements and enhances these studies by adding a layer of transparency that improves trust in the AI-driven energy management systems. This study contributes to the growing body of work on AI applications in NZEBs by providing a clear methodology for developing transparent and XAI models that can enhance both operational performance and stakeholder confidence.

Yu et al. [77] focus on the design and implementation of an AI-based control strategy for NZEBs within a smart microgrid framework. The authors propose integrating a supervisory control and data acquisition (SCADA) system for online monitoring of energy consumption and environmental parameters. The control strategy aims to optimize power management and heat recovery efficiency. The study demonstrates the practical application of this strategy in several NZEBs, showcasing its ability to manage energy more effectively within the context of a smart microgrid. This study extends previous research by integrating AI with SCADA systems for dynamic monitoring, providing a more comprehensive solution for managing energy within NZEBs. The practical implementation in real-world projects enhances the applicability of AI solutions discussed by Wang et al. [81], who emphasize the integration of energy management systems in ZEBs.

A hybrid optimization strategy aimed at enhancing the autonomy of NZEBs by minimizing grid energy dependency is presented by Georgiou et al. [74]. Their approach combines linear programming (LP) for real-time optimization of battery dispatch, ANNs for forecasting energy demand and PV generation, and genetic algorithms (GA) to refine the dispatch process. Moreover, the system advisor model (SAM) from the national renewable energy laboratory (NREL) was integrated to better capture battery behavior. Applied to a real building case study, the method successfully reduced annual grid energy usage by 53% and achieved 60% renewable energy coverage, showing that this integrated method significantly advances NZEB autonomy. This study contributes a comprehensive, real-time, hybrid solution for improving energy self-sufficiency in NZEBs.

Wu et al. [75] develop an intelligent optimization framework aimed at enhancing the performance of NZEBs. The framework integrates BIM with DesignBuilder and a hybrid ML approach combining RF and NSGA-III. The model optimizes multiple objectives (e.g., energy efficiency, comfort, environmental impact, and cost) through NSGA-III by simulating building design scenarios and predicting their performance with RF A case study of a building validates the method, demonstrating significant energy savings (21.25%) and high model accuracy (*R*^2^ values between 0.91 and 0.93). This integrated approach offers a powerful tool for the multi-objective design optimization of NZEBs.This study extends the work of Georgiou et al. [74] by introducing a hybrid ML and optimization framework that targets multiple performance goals simultaneously, not just energy management. The use of RF and GA also aligns with the predictive and optimization techniques discussed in Mousavi et al. [80] and Ibrahim et al. [76].

Qin et al. [71] aim to enhance energy efficiency in NZEBs by improving the accuracy of heating and cooling load predictions, which are crucial for optimal control of HVAC systems. The authors apply four ML methods—multivariate polynomial regression, SVR, multilayer perceptron, and XGBoost—to build datasets. The study highlights the significance of feature selection in enhancing model accuracy and simplifying input complexity. Real-world factors such as occupancy changes and weather uncertainties are also considered. Results indicate that proper feature selection significantly enhances model performance, while deployment challenges, such as thermal inertia effects, must be addressed to achieve consistent prediction accuracy. This study advances the predictive modeling strand found in Georgiou et al. [74] by conducting a direct comparative analysis of multiple ML models tailored for NZEB load forecasting. Its detailed attention to feature selection and real-world deployment challenges resonates with the transparent AI modeling concerns raised by Kermiche et al. [78]. In addition, its focus on HVAC load forecasting aligns with the smart grid and energy management applications presented in Yu et al. [77], while diverging in its emphasis on predictive rather than control strategies.

Chegari et al. [73] develop a multi-objective optimization approach for NZEBs that balances minimizing energy consumption, maximizing thermal comfort, and enhancing energy self-sufficiency. Their method uses a surrogate model based on ANNs and optimizes it through multi-objective particle swarm optimization (MOPSO). Applied to residential buildings across different climate zones, their approach significantly improves building performance metrics, achieving average reductions of 75% in energy consumption, 50% in thermal comfort, and 85% in self-sufficiency. The study highlights the practicality and adaptability of their surrogate-model-based optimization framework for architects, engineers, and designers aiming to create energy-resilient and comfortable NZEBs. This study expands on the optimization strategies explored by Georgiou et al. [74] and Wu et al. [75], particularly by integrating surrogate modeling to streamline the optimization process for NZEBs. Their use of ANNs and MOPSO aligns with broader AI-driven optimization trends seen in Mousavi et al. [80], but Chegari et al. [73] specifically differentiate themselves by focusing on balancing energy, comfort, and self-sufficiency simultaneously rather than prioritizing a single objective. Furthermore, their emphasis on practical, climate-adapted solutions echoes the regional adaptability concerns raised by Ibrahim et al. [76].

Alden et al. [70] introduce a novel deep learning-based method for separating HVAC energy consumption from total residential loads, aiming to enhance home energy management systems (HEMS) in smart and NZE homes. The authors develop LSTM encoder-decoder models that utilize future weather data instead of standard weather forecasts to accurately predict both HVAC and PV energy usage. Utilizing the extensive SHINES dataset, the proposed method achieves low prediction errors well within recognized academic and ASHRAE standards. In addition to improving energy monitoring, their approach also demonstrates the ability of smart homes to act as dispatchable loads or energy generators within a virtual energy operation framework. This study enhances the forecasting capabilities essential for smart NZE home management, closely paralleling the goals outlined by Qin et al. [71], who also emphasized accurate HVAC load predictions using diverse ML models. Their integration of LSTM networks aligns with the AI-driven predictive frameworks presented in Kermiche et al. [78] and Wu et al. [75]. However, Alden et al. [70] focus on real-time energy separation and management, extending these prior works by offering actionable solutions for existing residential infrastructures without specialized HVAC submetering.

Overall, the reviewed studies in the two subsections demonstrate the growing sophistication and diversity of AI and ML applications in the pursuit of ZEBs, nZEBs, NZEBs, and PEBs. From predictive load forecasting and energy optimization to explainable frameworks and smart control strategies, these works expand, diversify, and deepen the understanding of the field as to how intelligent systems can transform building performance. While each study offers distinct methodological advancements and focuses on different aspects, such as transparency, optimization, or real-time control, they all converge on the critical goal of enhancing energy efficiency, autonomy, and occupant comfort. These insights underscore the importance of integrated, data-driven solutions in overcoming current technical and practical barriers, thereby establishing a robust foundation for future research and real-world deployment in sustainable building development, which advances environmental goals.

#### 5.2.4 Artificial intelligence-powered digital twins in building systems: applications for advancing smart, green, and zero-energy building environments

The integration of AI and DT technologies within building systems is reshaping the landscape of sustainable smart built environments. These technologies enable the optimization of building operations through real-time data analysis, predictive modeling, and intelligent decision-making, which are essential for advancing environmental goals. They play a key role in enhancing energy efficiency, resource management, and performance optimization in buildings, making them more sustainable and intelligent by reducing energy consumption, improving comfort, and meeting environmental targets. The reviewed studies explore the key contributions and implications of AI and DT applications across diverse building typologies, highlighting their potential to drive sustainability and foster adaptive built environments.

Deena et al. [82] and Agostinelli et al. [25] both explore AI-driven DT applications in energy management, specifically in residential buildings. Agostinelli et al. [25] investigate DT approaches for residential districts, analyzing energy efficiency interventions and how DTs help assess energy production from renewable sources to meet nZEB criteria. Similarly, Deena et al. [82] focus on neighborhoods, showcasing how AI, combined with IoT and DT technologies, can optimize energy consumption in buildings, with a particular emphasis on achieving NZEB standards. They model various energy-efficient scenarios to ensure optimal comfort levels while minimizing energy consumption. Shen et al. [34] take a step further, focusing on how DT can optimize PEDs, which integrate energy systems across entire neighborhoods or districts. The authors highlight the role of DTs in coordinating multiple systems (e.g., energy, transportation) and improving urban sustainability. This study aligns with Arowoiya et al.’s work [26] by demonstrating how DT can be scaled up from individual buildings to entire districts, thereby driving sustainable urban development. It emphasizes the role of AI and big data in optimizing PEDs, with applications in real-time analysis and sustainability goals.

De Wilde [84] presents a comprehensive review of building performance simulation in the context of AI and DTs. This study critiques and synthesizes emerging trends in building simulation, identifying conceptual overlaps and distinctions between DT technology and traditional simulation methods. This work bridges the gap between simulation and real-world applications of AI in building performance, particularly in the context of energy and sustainability. It complements the more specific case studies mentioned earlier, such as those by Deena et al. [82] and Agostinelli et al. [25], by providing a theoretical foundation for integrating AI and DTs into building performance modeling. The study underscores the role of these technologies in enhancing building energy management and sustainability at both the building and district levels.

Seen from a different perspective, El-Gohary et al. [29] extend the application of DTs into energy consumption prediction in residential buildings, specifically under the influence of climate change. The authors employ an ANN in the DT model to predict energy use, highlighting the role of AI in understanding energy patterns across different materials and designs. This is particularly important in the context of green building design, where minimizing energy consumption is crucial to reducing environmental impact. This study expands the scope of earlier works by focusing on predictive modeling, enabling engineers and architects to select materials that optimize energy use. In a recent systematic review, Semeraro et al. [184] address the role of DT technology in advancing smart and green buildings, focusing on its potential to enhance sustainability, energy efficiency, performance monitoring, and occupant well-being. The study shows that DT applications in green and smart buildings are primarily supported by BIM, AI, and IoT, which enable real-time data integration, automation, and system optimization. DTs are being applied across various areas, including energy management, predictive maintenance, occupant-centered control, and environmental monitoring. The findings also reveal that while DTs hold strong promise for achieving net-zero energy performance and waste reduction goals, most current studies remain conceptual or simulation-based, with limited large-scale empirical validation. This study aligns with De Wilde [84], who highlights the conceptual gaps between DTs and traditional simulation methods, and complements El-Gohary et al. [29], who demonstrate the predictive potential of AI-powered DTs in addressing energy consumption under climate change scenarios.

Arsiwala et al. [27] explore the application of DT and ML to monitor CO_2_ emissions in existing buildings. Their study emphasizes the importance of monitoring carbon emissions and optimizing the operational energy performance of buildings to meet net-zero goals. It adds a new dimension to the discussion by highlighting the use of AI not only for energy optimization but also for reducing the environmental footprint of buildings. It complements the findings of the study by Alnaser et al. [5], who focus on the use of AI-powered DTs in smart cities, by introducing a practical application for carbon footprint management in existing building stock, reinforcing the broader goal of carbon neutrality and sustainability in the built environment. Alnaser et al. [5] discuss how DTs are being used in construction, facility management, and energy optimization for ZEBs, further supporting the shift towards more resilient and sustainable urban ecosystems. Their study ties together the findings from previous studies by focusing on the intersection of DTs, AI, and IoT, emphasizing the need for smart city frameworks to achieve urban sustainability.

Dinmohammadi and Shafiee [28] expand on the application of AI and DT by addressing thermal comfort and energy consumption in residential buildings under varying indoor and outdoor conditions. Their study extends beyond energy prediction by incorporating real-time data from sensors and IoT devices to optimize both thermal comfort and energy consumption. This contributes to the sustainability discussion by emphasizing the occupant experience in energy-efficient building designs, while also using DTs for monitoring and predicting building performance. Likewise, Arowoiya et al. [26] focus on thermal comfort and energy efficiency, performing a comprehensive review of DT technology in buildings. The authors identify the need for more research on human-centered approaches, such as occupant perceptions of comfort, and advocate for more refined algorithms to improve predictive accuracy. Their work complements the findings of Dinmohammadi and Shafiee [28] by emphasizing the importance of occupant well-being in smart building designs, particularly in the context of thermal comfort and energy management.

Jafari et al. [83] propose a novel DT architecture integrated with asset management and building simulation technology to optimize building performance and energy usage. Their approach aligns with the broader theme of integrating real-time data and AI algorithms to improve the operational control of buildings, similar to the DT and AI-driven systems explored in earlier studies. The study focuses on asset performance and maintenance strategies for both new and existing buildings, contributing to the understanding of how DT technologies can enable predictive maintenance, energy efficiency, and cost savings in buildings. This work also complements the study by Alnaser et al. [5], who advocate for smart city applications of DTs, by focusing specifically on how the latter can be used in asset management and building operations.

AI-driven DTs play an important role in enhancing the environmental goals of SGZEBs. In the context of smart buildings, they offer real-time monitoring and optimization capabilities, enabling adaptive energy management and operational efficiency. Smart buildings can automate responses to environmental variables, enhance occupant comfort, and optimize resource usage, integrating AI with DTs, aligning with the core principles of smart building design. For green buildings, these technologies enable precise environmental monitoring, allowing for more efficient use of renewable resources and a reduced environmental footprint. They provide data-driven insights that help in achieving sustainability benchmarks, such as lower carbon emissions and improved energy usage. In ZEBs, AI-powered DTs help track energy production and consumption to ensure that energy produced from renewable sources meets or exceeds the building's consumption. This integration enables continuous optimization, allowing buildings to remain self-sufficient while contributing to broader environmental sustainability efforts. DTs push these building typologies towards more adaptive, efficient, and resilient futures by supporting data-driven, dynamic decision-making.

### 5.3 Artificial intelligence-digital twin integration for environmentally sustainable smart built environments and cities

This subsection presents a unified framework that integrates AI and DT technologies to advance environmental goals in smart buildings. It also explores the broader implications of this framework for sustainable urban development and environmentally sustainable smart cities.

#### 5.3.1 A framework for environmentally sustainable smart built and urban environments as enabled by artificial intelligence-digital twin integration across smart, green, and zero-energy buildings

**Fig. 6:**
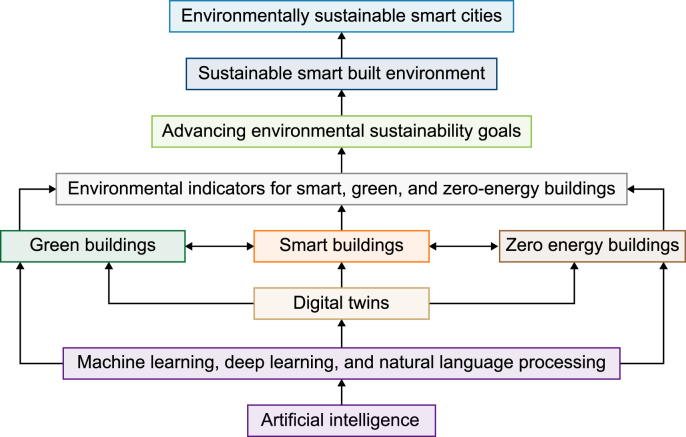
A framework for environmentally sustainable smart built and urban environments, as enabled by artificial intelligence-digital twin integration across smart, green, and zero-energy buildings.

The proposed framework (Fig. 6) is developed based on insights gained from the analysis and synthesis of recent interdisciplinary literature presented in the two parts of the results section. It is grounded in the application and integration of AI and DTs as foundational technologies to advance the development of environmentally sustainable smart built environments. Both AI and DTs serve as critical enablers in terms of enhancing the intelligence, adaptability, and operational efficiency of built assets within urban ecosystems.

At the foundation of the framework lies AI, which provides the essential computational capacity for perceiving, learning, reasoning, and decision-making. Building on this base, the next layer comprises AI subdomains, such as ML, DL, and NLP, that supply the methodological tools and algorithmic models for extracting patterns, generating predictions, performing classifications, and enabling intelligent interactions from the diverse datasets collected through sensors, IoT devices, and BMS. In the subsequent layer, DTs establish dynamic, real-time virtual representations of physical buildings and infrastructure, continuously synchronized with real-world conditions. Operating in unison, AI and DTs drive predictive analytics, real-time monitoring, anomaly detection, scenario simulation, adaptive optimization, and strategic planning, thereby forming the intelligence backbone of the framework.

These technologies are deployed across three key building typologies: smart buildings, where AI and DTs enable intelligent control over energy consumption, HVAC systems, lighting, occupant comfort, and security; green buildings, where sustainability-oriented performance metrics, such as water conservation, indoor air quality, material circularity, and waste minimization, are continuously monitored, analyzed, and improved; and ZEBs, where AI and DT simulations balance renewable energy generation and energy consumption, dynamically adjusting operations to maintain a net-zero or positive energy balance. The principles underlying each typology reinforce one another (↔): the data-driven, adaptive control of smart buildings supports the sustainability objectives of green buildings; the resource efficiency and circularity principles of green buildings inform the energy self-sufficiency and balancing strategies in ZEBs; and the renewable energy integration and dynamic optimization of ZEBs feed back to enhance operational intelligence and occupant-centered strategies in smart buildings. Together, these interactions create a continuous loop of principle-based improvement that strengthens both performance and sustainability across building systems.

Through these applications, AI and DTs directly influence and improve critical environmental indicators, including energy efficiency and demand reduction, renewable energy integration and storage optimization, carbon footprint reduction and net-zero strategies, water efficiency and resource management, indoor environmental quality and thermal comfort, as well as predictive maintenance and lifecycle optimization. These technologies enable buildings to meet and adaptively exceed established environmental benchmarks and certification standards (e.g., see Ref. [69] for a systematic review) by facilitating continuous monitoring, predictive interventions, and evidence-based decision-making.

The framework operates through a dynamic feedback loop, wherein AI learns from continuously updated DT data, simulations inform future planning and operational strategies, and real-world building performance feeds back into refining AI models. This self-improving cycle ensures that buildings and urban spaces are not static achievements but living systems that evolve in response to environmental conditions and shifts. Overall, the framework provides a transformative pathway towards achieving broader environmental goals in urban environments, promoting climate action, and fostering sustainable development by leveraging the synergies among AI, DTs, and SGZEB practices.

The ultimate contribution of this integrated system is the realization of environmentally sustainable smart built environments characterized by interconnected, adaptive, and self-optimizing assets that operate within a larger urban ecosystem committed to sustainability, resilience, and human well-being. Buildings are no longer isolated entities but active nodes within a responsive, data-driven network that collectively advances resource efficiency, climate resilience, and the quality of life.

This framework does not operate in isolation but is integral to the broader vision of environmentally sustainable smart cities. AI and DT technologies enable cities to be both technologically advanced and ecologically responsible by integrating intelligent, self-adaptive buildings into a cohesive urban ecosystem. They enable a systemic transition from fragmented sustainability efforts towards fully integrated, city-wide environmental management strategies. SGZEBs thus act as foundational components of a larger, interconnected urban fabric, one that actively enables carbon neutrality, promotes circular resource flows, supports climate resilience, enhances biodiversity, and contributes meaningfully to global environmental and climate goals. Through continuous innovation, data-driven adaptability, and human-centered design, the framework envisions smart cities as living systems that can thrive in harmony with both people and the planet. Interconnected, adaptive building systems actively contribute to holistic urban sustainability objectives. By ensuring that built environments dynamically interact with and support broader urban flows, such as energy grids, mobility systems, material usage, and water networks, AI and DT technologies enable the emergence of regenerative urban ecosystems. Thus, the proposed framework offers not only a micro-level roadmap for sustainable building performance but also a macro-level strategy for reshaping cities as integrated, intelligent, and environmentally restorative entities.

#### 5.3.2 Connecting the framework to environmental sustainability, sustainable development, and sustainable smart cities

The proposed framework, centered on the integration of AI and DTs across SGZEBs, directly advances the broader goals of environmental sustainability, sustainable development, and the creation of smart cities. Recent scholarly contributions reinforce and contextualize the framework's relevance in these domains.

Bibri [9] explores how AI and AIoT technologies drive the development of smarter eco-cities by embedding circular economy principles, metabolic circularity, and tripartite sustainability into urban systems. These technologies are shown to enable resource optimization, waste minimization, and reduced environmental impacts, fostering resource-efficient urban environments. These principles are central to the framework's building-level and city-scale environmental integration. Building on this, Bibri [8] investigate the role of both AI and AIoT in advancing the environmental performance of emerging smarter eco-cities. The study highlights how AI and AIoT solutions can optimize resource use, enhance infrastructure efficiency, monitor environmental parameters, reduce carbon footprints, and foster climate resilience. This study aligns with the framework's dynamic environmental monitoring and operational optimization of SGZEBs by enabling real-time emission reductions and enhancing urban resilience. Bibri et al. [1] extend this perspective by emphasizing the synergistic interplay of AI, AIoT, and UDTs in data-driven environmental planning in sustainable smart cities. They demonstrate how the integration of these technologies reshapes sustainable urban development by enabling adaptive planning, enhanced environmental monitoring, and dynamic decision-making. This underscores the transformative role of AI- and AIoT-driven DT in aligning smart city functions and domains with environmental goals, directly supporting the core foundation of the proposed framework.

In a more focused study, Alnaser et al. [5] provide further evidence for the role of AI-powered DT in sustainable buildings and smart cities. They highlight how the integration of AI, IoT, and DTs enables energy optimization, enhances building resilience, and supports sustainability in urban environments. These insights contribute directly to the framework's emphasis on energy efficiency and resource management across different spatial scales. Kumar et al. [185] add to this perspective by illustrating how AI-driven DT can simulate and optimize resource use and energy consumption in real time, among others. This strengthens the framework's operational foundation for urban efficiency and climate-responsive infrastructure.

In addition, Matei and Cocoşatu [12] complement these findings by examining the role of sensor-based DT systems and their integration with AIoT, intelligent decision-making algorithms, and cloud networks in environmentally sustainable urban management. Their study highlights how data-driven urban computing frameworks facilitate real-time environmental monitoring, predictive analytics, and decentralized control, which are essential to the framework's AI-DT integration layer. While also acknowledging participatory dimensions, it primarily highlights how interconnected sensor networks and digital ecosystems support environmentally informed governance, aligning with the framework's emphasis on adaptive and ecologically responsive smart city operations. Supporting these insights, Thamik et al. [18] further discuss AIoT's role in advancing environmental protection, renewable energy systems, and smart community development, thus aligning with the framework's focus on interconnected, adaptive urban environments. Expanding on this, Mishra et al. [17] demonstrate how AIoT technologies enhance energy efficiency, promote renewable energy transitions, and support circular economy models. This complements the established role of IoT and big data analytics in advancing environmental solutions for sustainable smart cities, including buildings, particularly in enhancing energy efficiency and reducing carbon emissions [186]. IoT and big data analytics are complementary and integrated, with IoT generating data and analytics extracting actionable insights in a continuous loop that supports real-time, predictive, and adaptive decision-making. They provide a holistic, system-level understanding of sustainable urban development paradigms, making complex city systems measurable, knowable, and tractable in terms of their operations and management, thereby enhancing resilience, efficiency, and livability [187,188]. Applying this same data-driven, integrative approach to building-scale, AI-driven DT frameworks enables coordinated optimization of building performance, energy management, and environmental outcomes across different scales in smart cities (see, e.g., Ref. [5]).

From a broader perspective, Shaamala et al. [189] emphasize AI's capacity to enhance green infrastructure, with applications in air quality monitoring, biodiversity preservation, urban heat island mitigation, and energy-efficient design. Their proposed environmental planning framework complements the optimization strategies embedded within the AI-DT dynamic feedback loops of the framework. Extending these applications, Nti et al. [190] examine AI's role in sustainable resource management, specifically water conservation, energy optimization, and transportation efficiency. Their focus on AI-based decision support systems strengthens the framework's predictive, adaptive management capabilities. Further, Yadav and Singh [191] stress the need for advanced AI decision-making tools for climate change mitigation and disaster resilience—challenges that the framework's continuous learning and predictive analytics aim to address.

Chaudhary [192] highlights AI's broad applications across clean energy and pollution control, emphasizing its critical role in achieving the SDGs. This perspective aligns with the framework's integrated and continuously adaptive approach, which addresses environmental objectives of sustainable development across multiple spatial scales. Kumari and Pandey [193] also focus on AI's potential in pollution control and clean energy, as well as natural resource management, within the SDGs framework. Thamik et al. [18] and Mishra et al. [17], as discussed earlier, contribute further to the role of AIoT in fulfilling SDGs through urban sustainability innovations, including buildings and smart grids. Together, these studies provide empirical and conceptual support for the framework, demonstrating how AI and AIoT can operationalize sustainable development objectives across urban systems and building scales.

Several other recent studies highlight the innovative role of AI in advancing sustainable energy systems and reinforcing key objectives of the framework. Wan et al. [194] demonstrate AI's application in improving the environmental sustainability of large-scale solar energy systems. Their findings align with the framework's emphasis on renewable energy integration within zero-energy and green building typologies. Anbarasu et al. [195] similarly examine AI's significant impact on bioenergy systems, feedstock management, and energy optimization, supporting the framework's vision for clean, efficient, and low-impact energy systems across the built environment. Rasheed et al. [196] examine how AI can balance industrial development with environmental responsibility, particularly by enhancing energy efficiency and promoting renewable energy. These two studies reinforce the framework's integrated approach, demonstrating how AI applications can advance sustainable energy management and environmental performance across multiple sectors of the built environment.

On the whole, these diverse yet interconnected studies affirm the relevance and potential of the proposed framework. The framework provides a strategic pathway for realizing environmentally sustainable smart cities by operationalizing AI- and AI-driven DT optimization across both building and urban scales. It enhances micro-level building performance and facilitates broader systemic transformations towards resource efficiency, climate resilience, circularity, and urban ecological restoration, ultimately advancing the global agenda for environmentally sustainable urban development.

## 6 Discussion

The increasing urgency to mitigate climate change impacts, promote environmental sustainability, and enhance urban resilience has positioned the building sector at the forefront of global sustainability efforts. In this context, this study systematically examined how AI and AI-driven DT technologies are advancing environmental goals across SGZEBs. By analyzing and synthesizing diverse research streams, it offers an integrated understanding of the transformative role of these technologies in optimizing building performance, enhancing resource efficiency, and supporting broader SDGs. This discussion elaborates on the key findings, interprets their significance, compares them with previous research, as well as outlines their implications for research, practice, and policymaking. It also reflects on the challenges and limitations identified and proposes directions for future research in this rapidly evolving field.

### 6.1 Summary of the key findings and their interpretation

This study revealed key patterns, trends, and areas of technological convergence, providing insights into how AI-driven strategies are shaping more adaptive, efficient, and low-impact building systems. The findings also highlight the interconnected role of AI and DTs in operationalizing broader environmental goals at the building and urban scales. A detailed discussion of the findings and their interpretation follows.

As for RQ1, the study found that AI and ML play a critical role in enhancing the environmental performance of smart buildings. AI technologies support optimized energy management through adaptive control strategies that respond dynamically to environmental conditions and occupant behaviors, leading to more efficient and self-regulating building operations. AI also facilitates the integration of renewable energy by improving supply-demand forecasting and optimizing storage systems, thus promoting cleaner energy use. Furthermore, AI enables occupant-centered design by personalizing environmental controls to balance comfort with sustainability goals. Lastly, predictive system control powered by AI allows smart buildings to anticipate operational needs and proactively maintain efficiency, highlighting AI's capacity to future-proof building performance in dynamic settings. This collected evidence suggests that AI fundamentally transforms smart buildings from reactive infrastructures into proactive, self-optimizing systems capable of contributing to achieving environmental goals. AI empowers smart buildings to continuously align with sustainability objectives in dynamic and complex environments. This positions AI as both a support tool and a core driver of systemic change towards low-carbon, energy-efficient, and resilient built environments.

Regarding RQ2, the study highlighted that AI is increasingly integrated into green building practices, spanning diverse domains. Concerning Subsection 5.2.2.1, recent studies show that AI applications in green building design span predictive modeling, compliance support, optimization frameworks, and critical reflections on creativity. AI can streamline sustainability compliance, improve certification outcomes, reduce emissions, and lower operational costs through predictive analytics and optimization. ML models accelerate the design process and enhance performance prediction, while BIM-integrated frameworks achieve multi-objective optimization across energy use, cost, carbon emissions, and thermal comfort. Advanced DL models outperform conventional approaches in improving environmental performance and occupant comfort. At the same time, AI adoption may reduce creativity and professional agency, highlighting the need for careful, human-centered implementation. These findings indicate that AI in green buildings is evolving from isolated predictive tools into integrated decision-support and optimization systems capable of balancing multiple sustainability goals. However, technological efficiency must be paired with human creativity, judgment, and professional integrity. The future of AI in sustainable architecture likely lies in hybrid approaches that combine advanced optimization with human-centered design, ensuring both environmental performance and cultural richness in the built environment.

In connection with Subsection 5.2.2.2, recent research demonstrates that AI significantly enhances energy efficiency, waste management, thermal comfort, and sustainability in green buildings. AI-based energy management models improve the efficiency of heating, cooling, and HVAC systems while reducing overall energy consumption and maintaining high predictive accuracy. Predictive frameworks for biogas production using AI and dynamic control systems optimize energy recovery from organic waste, supporting both sustainability and resource efficiency. AI-driven waste management strategies show that composting is the most environmentally friendly option, while incineration and landfilling can better meet social and economic goals, ultimately reducing landfill use and overall energy demand. ML optimization of advanced insulation materials greatly improves thermal performance, leading to substantial energy savings and reductions in carbon emissions. Active learning models for predicting heating and cooling demand enable more efficient building operation, lower energy consumption, and decreased carbon footprints. Multi-objective optimization frameworks help balance energy efficiency, cost-effectiveness, emissions reduction, and occupant comfort, while AI integration with renewable energy and supportive policies can further enhance long-term sustainability outcomes.

These findings indicate that AI has a transformative role in making green buildings more sustainable and efficient. AI enables these buildings to operate at their optimal performance, thereby reducing waste and unnecessary energy use. The integration of AI in waste management and energy recovery demonstrates that technology can simultaneously address environmental, social, and economic objectives (see Ref. [9]). Optimization of materials and design parameters through ML improves energy efficiency and supports reductions in carbon emissions, contributing to climate goals. Active learning and multi-objective frameworks demonstrate that AI can effectively balance competing priorities, thereby providing a holistic approach to building design and operation. In essence, AI is a strategic enabler for sustainable, resource-efficient, and human-centered green buildings.

In relation to Subsection 5.2.2.3, recent research shows that AI can greatly improve green building project outcomes by improving cost estimation accuracy, providing probabilistic forecasts, and optimizing cost control. AI-based frameworks effectively manage complex, non-linear relationships between numerous cost-influencing factors, achieving high prediction accuracy across different algorithms, including neural networks, gradient boosting, and RF. In addition, AI facilitates comprehensive risk assessment by identifying and quantifying critical project risks such as economic, market, functional, and fire-related hazards. Hybrid approaches that combine expert judgment with ML further increase predictive reliability by enabling the prioritization of key risks and the optimization of mitigation strategies. As a whole, AI applications demonstrate strong potential for reducing uncertainties, improving decision-making, and supporting sustainable construction practices.

These findings suggest that AI is no longer merely a supportive tool but a strategic enabler in sustainable green building management. Project managers can proactively address both cost and safety challenges by integrating predictive analytics with risk assessment, thereby reducing resource waste and enhancing project efficiency. The convergence of multiple AI techniques underscores the importance of tailored, context-specific applications, where models are selected based on project needs and available data. Furthermore, the evidence points towards a shift in professional practice: reliance on AI can enhance human decision-making without replacing critical judgment, ultimately fostering a more robust, data-driven approach to green building design and construction.

As regards RQ3, the study demonstrated that AI has become central to the optimization and management of ZEBs, nZEBs, PEBs, and NZEBs. As to Subsection 5.1.3.1, recent research underscores the central role of AI-driven technologies in advancing energy efficiency, renewable energy integration, and intelligent energy management. EEMs are shown to reduce building demand through advanced design strategies, material innovations, and HVAC optimization, while RETs such as solar, wind, biomass, and geothermal are increasingly integrated through distributed generation systems. BEMS enhanced by AI play a critical role in monitoring, fault detection, and performance optimization. Advanced predictive models, including neural networks, model predictive control, and ML algorithms, enable real-time optimization of energy consumption, accurate forecasting of renewable production, and occupant-aware adaptation. Case studies demonstrate cost-effectiveness, with systems producing more energy than consumed, reducing or eliminating energy costs, and achieving favorable payback periods. In addition, data-driven frameworks emphasize balancing energy demand reduction, renewable supply optimization, occupant comfort, and economic performance to achieve net-zero or positive energy outcomes at both building and district scales.

These findings underscore the transition from static, efficiency-focused strategies towards dynamic, AI-enhanced building ecosystems capable of self-learning, adapting, and optimizing energy use in real time. The integration of AI with BEMS and distributed renewable systems demonstrates that ZEBs are no longer only about minimizing consumption, but also about intelligently managing generation, storage, and distribution to create resilient, cost-effective, and environmentally aligned buildings. The emphasis on predictive modeling and adaptive control points to a model where buildings function as active participants in energy networks, capable of balancing supply and demand, anticipating environmental conditions, and enhancing user comfort. This convergence pushes ZEBs and PEBs beyond compliance-oriented sustainability, positioning them as key enablers of future smart cities and carbon-neutral energy systems.

In regard to Subsection 5.1.3.2, research shows that AI and ML are key to optimizing NZEB performance across multiple dimensions, including predictive energy analytics, intelligent HVAC systems, and real-time energy management. Hybrid optimization methods, surrogate models, and predictive frameworks enhance energy efficiency, renewable energy integration, and building autonomy. XAI frameworks and advanced forecasting models improve transparency, trust, and deployment accuracy in real-world contexts. Multi-objective optimization approaches balance energy consumption, thermal comfort, and self-sufficiency, while smart home management systems enable buildings to act as flexible energy resources. Region-specific strategies, load forecasting improvements, and integration with smart grids and microgrids further demonstrate the practical applicability of AI in achieving net-zero and positive-energy objectives.

These findings indicate that NZEBs are evolving into adaptive, data-driven systems that leverage AI and ML for operational efficiency, occupant comfort, and environmental performance. The convergence of various techniques allows buildings to actively manage energy flows, integrate renewable sources, and minimize grid dependence. The inclusion of XAI frameworks and climate-adapted solutions emphasizes that technical performance must be paired with transparency, stakeholder trust, and contextual adaptability. Overall, these studies illustrate a shift from isolated energy-efficiency measures towards comprehensive, intelligent, and resilient building systems, setting a strong foundation for large-scale deployment of sustainable buildings.

With respect to RQ4, the study revealed that AI-driven DTs in smart buildings drive performance optimization through automation, predictive maintenance, and occupant-centered control. Applications include thermal comfort optimization, environmental monitoring, and system integration via IoT and BIM, which enable adaptive decision-making and resilience in building operations. Although promising, most studies remain simulation-based, pointing to a need for large-scale empirical validation. In green buildings, AI–DT integration enhances energy efficiency, reduces carbon emissions, and improves occupant comfort by enabling predictive modeling, real-time monitoring, and intelligent material selection. Studies highlight applications in energy consumption prediction under climate change, CO_2_ emissions tracking, and resource optimization, while also emphasizing the importance of occupant well-being and human-centered design. For ZEBs and PEDs, AI-DT integration provides advanced energy management and strategic planning by balancing renewable energy production with demand across both buildings and neighborhoods. Case studies show how AI–DT models optimize district-scale systems, integrating energy, transportation, and resource flows to achieve net-zero or positive energy goals.

These findings suggest that the integration of AI and DTs offers innovative potential for advancing sustainability in the built environment. They allow buildings to minimize consumption and actively contribute to net-zero and positive energy goals. At larger scales, they provide the infrastructure for smart cities and sustainable districts, where multiple systems can be integrated for improved resilience and efficiency. The emphasis on occupant comfort and carbon reduction shows that DTs are not limited to technical performance but extend to human-centered and environmental priorities. However, the predominance of simulation-based evidence indicates that more large-scale, real-world applications are necessary to validate their potential. Taken together, AI-powered DTs represent a key step towards adaptive, efficient, and sustainable building ecosystems.

Concerning RQ5, the proposed framework demonstrates that AI- and DT-enabled SGZEBs can advance the goals of environmental sustainability, sustainable development, and sustainable smart cities. AI and AIoT technologies are shown to optimize resource use, minimize waste, integrate renewable energy, and enhance energy efficiency at both building and urban scales. AI supports circular economy integration, real-time emissions reduction, climate resilience, and green infrastructure. At the urban scale, interconnected AI- and DT-enabled SGZEBs facilitate the creation of energy-positive districts, support climate-resilient infrastructure, and enable sustainable resource management. They also align operational building performance with broader sustainability and SDG objectives. By combining AI and DT capabilities, SGZEBs serve as both local and networked sustainability enablers. At the building level, they reduce environmental impacts and operational costs. When integrated across neighborhoods or cities, they provide aggregated benefits, including grid stabilization, reduced emissions, and enhanced urban resilience. This dual impact demonstrates that AI-DT-enabled SGZEBs are a practical pathway for advancing sustainable smart cities: they translate real-time data and predictive intelligence into actionable strategies that connect micro-level efficiency with macro-level sustainability goals.

The framework was designed to address critical sustainability challenges such as resource efficiency, climate change mitigation, energy resilience, and ecological health. It supports the transition to sustainable smart cities by operationalizing sustainable energy integration, fostering circular economy practices, and enabling real-time environmental responsiveness. This cross-scale, dynamic approach ensures that buildings and urban spaces evolve from static entities into intelligent, self-optimizing systems that contribute proactively to sustainable development objectives. The framework provides a transformative pathway towards building energy-efficient, resilient, and ecologically restorative cities by embedding AI and DTs at the core of urban systems.

### 6.2 Comparative analysis: advancing beyond fragmented AI applications towards a holistic framework for sustainable smart built and urban environments

While numerous studies have explored the role of AI and DTs in smart and sustainable buildings, the majority adopt a fragmented or domain-specific approach by focusing narrowly on applications, such as energy management, HVAC optimization, building automation, AI–IoT integration, and/or DT simulations, in isolation.

While Sleem and Elhenawy [42] primarily emphasize AIoT-driven operational efficiency, predictive analytics, and security optimization, they do not examine AI's systemic potential to achieve broader environmental or net-zero energy goals. This study extends these findings by demonstrating how AI-driven automation can simultaneously optimize building performance, renewable energy integration, and support carbon-neutral objectives. Similarly, Qolomany et al. [87] highlight ML and big data applications in building automation, showing predictive analytics for real-time decision-making, but they do not integrate these capabilities with sustainability and cross-building typology optimization. In contrast, this study explicitly connects AI-driven automation with energy efficiency, occupant comfort, and broader environmental goals.

In energy management, Alanne and Sierla [53] explore ML applications enabling adaptive energy management and using DTs as AI-powered optimization environments. Wang et al. [43] focus on DTs for carbon peak management in terms of monitoring emissions and modeling net-zero strategies. Although these studies recognize AI's role in energy efficiency and carbon reduction, they primarily approach these aspects from an energy management perspective and do not fully explore AI's broader impact on sustainable building performance. This study builds upon these studies by combining AI and DT-based optimization with a holistic sustainability perspective, coordinating energy management, renewable integration, and occupant comfort across multiple building typologies.

Regarding green and sustainable buildings, Rodríguez-Gracia et al. [89] provide a bibliometric mapping of AI applications, and Debrah et al. [55] combine bibliometric and systematic analyses to identify trends, gaps, and future directions, including DTs, AIoT, blockchain, robotics, and ethical considerations. Wu et al. [90] further position AI as a driver of GBTI, while Hua et al. [91] demonstrate AI's role in reducing carbon emissions and improving operational efficiency. Although these studies highlight AI's potential in green buildings, they primarily focus on predictive modeling and thematic analyses, without fully synthesizing AI applications across environmental indicators or in relation to other building typologies. This study advances these findings by providing a comprehensive framework that integrates AI-driven strategies for emission reduction, energy optimization, waste minimization, sustainable materials, adaptive design, cost estimation, and risk assessment simultaneously, thereby operationalizing systemic sustainability objectives.

For net-zero and positive energy buildings, Mousavi et al. [80] show that data-driven prediction and optimization can greatly enhance energy efficiency and renewable energy integration. However, while their findings demonstrate energy savings at the building scale, they do not address how such gains translate into systemic sustainability outcomes across diverse building typologies. In contrast, this study extends these results by demonstrating how AI-enabled prediction and optimization can be embedded within a holistic SGZEB framework that aligns energy efficiency with carbon reduction, lifecycle sustainability, and cross-scale integration. Similarly, Bibri et al. [2] find that DT-based frameworks strengthen ZEB assessment in smart cities by improving energy management, enabling real-time monitoring, and facilitating renewable energy integration through AI, IoT, and CPS convergence. Yet, their results remain confined to performance assessments of ZEBs. This study builds on these insights by integrating AI-DT-enabled monitoring and predictive mechanisms into a broader sustainability framework that links ZEB optimization to built and urban goals, thereby achieving cumulative environmental benefits beyond isolated building-level improvements.

In sum, the existing literature predominantly analyzes individual AI technologies and isolated building functions, missing the opportunity to explore their holistic role in fostering environmentally sustainable smart built environments. Our study advances beyond these fragmented approaches by synthesizing AI applications across multiple building typologies and environmental sustainability indicators, integrating predictive, adaptive, and DT-driven mechanisms into a comprehensive framework that links micro-level building performance with macro-level urban sustainability goals. This framework addresses the fragmented nature of previous research by emphasizing the synergistic role of AI and DTs in optimizing resource use, reducing carbon footprints, enhancing adaptive operations, and enabling real-time environmental monitoring and circularity. Furthermore, it extends the discourse beyond individual building performance to the creation of interconnected urban ecosystems, contributing a fresh perspective that aligns with broader SDGs and provides a strategic roadmap for the realization of environmentally sustainable smart urban environments.

### 6.3 Implications of the proposed framework for research, practice, and policy-making

The proposed framework carries significant implications for advancing research, guiding practical implementation, and informing policy-making towards the realization of an environmentally sustainable smart built environment. In terms of research, the framework establishes a structured foundation for future interdisciplinary studies that further explore the integration of AI, DT, environmental science, and sustainable development across building and urban scales. It emphasizes the importance of developing dynamic, cross-scale models capable of capturing the complex interactions between intelligent building systems, urban ecosystems, and environmental outcomes. Researchers should also focus on designing new metrics, simulation environments, and performance evaluation tools that can assess the long-term ecological impacts of AI- and DT-enabled building systems in broader urban contexts.

From a practical perspective, the framework provides architects, urban planners, engineers, sustainability consultants, and facility managers with actionable guidance for designing, operating, and optimizing SGZEBs. It provides a roadmap for integrating AI and DT technologies into building management systems, enabling real-time, predictive, and adaptive optimization of energy consumption, environmental quality, resource flows, occupant comfort, and carbon emissions. Practitioners can leverage the framework to support operational resilience, enhance building lifecycle performance, and contribute more effectively to broader urban sustainability agendas. In addition, the framework supports the development of industry standards, certification schemes, and best practices that integrate intelligent and sustainable technologies into the planning, design, construction, and retrofitting of urban infrastructure.

For policy-making, the framework highlights the necessity of creating supportive regulatory environments, incentives, and investment strategies that promote the large-scale deployment of AI- and DT-enabled SGZEBs. It advocates for policies that ensure interoperability, ethical AI use, data transparency, and the alignment of digital innovation with global climate goals. Moreover, the framework underscores the importance of integrating intelligent building systems into municipal and national sustainable development strategies, climate action plans, and urban resilience frameworks. Policy-makers can enable the systemic transition towards dynamic, adaptive, and regenerative urban ecosystems that meet current sustainability targets by operationalizing the transformative and synergistic capabilities of AI and DTs.

### 6.4 Challenges, barriers, and limitations

While the proposed framework offers significant potential, several challenges, barriers, and methodological limitations need to be addressed to fully realize its impact. This subsection discusses these key issues from both a theoretical and practical standpoint, providing a comprehensive understanding of the obstacles researchers, practitioners, and policy-makers may encounter in the adoption and implementation of AI–DT technologies in the built environment.

#### 6.4.1 Technological and operational challenges

One of the primary challenges associated with the integration of AI and DTs in SGZEBs is the technological complexity involved in deploying these systems at scale. AI and DTs require substantial computational power, sophisticated algorithms, and vast amounts of high-quality, real-time data to function effectively. Collecting, processing, and integrating data from diverse sources, such as sensors, IoT devices, and BMS, presents significant operational difficulties. The integration of AI models with DTs must account for the diversity of building typologies, varying levels of data availability, and potential interoperability issues across different technologies and platforms. In addition, AI models used in these systems must be continually trained and updated to adapt to dynamic environmental conditions, building behaviors, and evolving energy demands. Ensuring that AI-driven systems remain effective and responsive to these changes in real-time is a major challenge, particularly when dealing with the large-scale, multi-functional nature of sustainable smart cities.

Furthermore, the adoption of AI and DT technologies requires a robust technical infrastructure that may not be available in existing buildings or urban settings. The need for significant investments in retrofitting existing infrastructure to integrate intelligent systems poses both financial and logistical challenges. Moreover, the complexity of operating these technologies across multiple scales, ranging from individual buildings and neighborhoods to entire city systems, demands an advanced level of coordination and synchronization. Ensuring seamless interaction between AI and DT systems across various building types and urban scales is a complex, ongoing challenge.

#### 6.4.2 Environmental risks and costs

While AI and DT technologies offer transformative potential for enhancing the environmental performance of buildings, they also introduce significant environmental risks and costs that must be carefully considered. The development, training, and operation of AI models, especially large-scale DL Systems, require substantial computational power, leading to high energy consumption and increased carbon emissions [[197], [198], [199], [200]]. Similarly, maintaining DT systems necessitates continuous data collection, transmission, storage, and processing, all of which contribute to considerable resource and energy demands over their lifecycle [1,[201], [202], [203]]. The production and disposal of sensors, IoT devices, servers, and other hardware components associated with DT ecosystems also raise concerns related to electronic waste (e-waste) and the depletion of critical raw materials. If left unmanaged, these hidden environmental costs could offset the environmental gains achieved through smarter and more sustainable building operations. Therefore, a paradox emerges: technologies intended to promote environmental sustainability may, without responsible lifecycle management and renewable energy sourcing, create additional burdens. To mitigate these risks, it is crucial to implement strategies such as green AI or computing practices (e.g., model efficiency optimization), sustainable DT design, renewable-powered data centers, e-waste recycling initiatives, and full lifecycle assessments for AI and DT deployments in the built environment.

#### 6.4.3 Data privacy and security and other ethical concerns

Data privacy and security are among the most pressing concerns in the integration of AI and DTs in the built environment. The collection and analysis of vast amounts of real-time data from sensors, IoT devices, and other monitoring systems pose significant risks to data protection, particularly due to the sensitive nature of information on occupants’ behaviors, energy consumption, and environmental conditions. There is an inherent challenge in safeguarding this data against potential breaches, unauthorized access, or misuse. In addition, the integration of AI and DTs often involves sharing data across different stakeholders, including building owners and operators, service providers, and government agencies, which may raise concerns about data ownership, accountability, and compliance with privacy regulations.

In addition to data privacy, there are concerns regarding the ethical use of AI. As AI systems are designed to make decisions based on large datasets, questions arise about the transparency and fairness of these algorithms. It is crucial to ensure that AI systems do not inadvertently perpetuate biases, leading to unfair or discriminatory outcomes, particularly in relation to energy distribution, resource allocation, and occupant comfort. To address these challenges, the study critically analyzes current mitigation strategies, including algorithmic transparency, bias detection protocols, ethical AI frameworks, and stakeholder accountability mechanisms. By systematically reviewing these approaches, the framework provides actionable guidance for researchers, practitioners, and policymakers to implement AI and DT systems responsibly, balancing technological advancement with privacy, fairness, and equity considerations. Addressing these ethical concerns requires the development of transparent and accountable AI systems, along with robust data governance frameworks that prioritize privacy, security, and fairness.

#### 6.4.4 Standardization and interoperability barriers

Another significant barrier is the lack of standardization and interoperability across the various technologies involved in AI and DT systems. The success of integrated sustainable smart building solutions relies on the seamless communication and data exchange between different hardware and software components, including sensors, IoT devices, BMS, AI models, and DT platforms. However, the lack of a universal set of standards for integrating these technologies can lead to compatibility issues between systems, hindering the scalability of AI and DT applications across various building typologies and urban environments. This lack of standardization also makes it difficult for stakeholders, such as building owners, developers, service providers, and regulatory bodies, to adopt and implement these technologies in a consistent and coordinated manner.

Efforts to establish industry-wide standards for AI and DTs in sustainable smart cities are still in the early stages, and their development is often fragmented. Without standardized protocols and frameworks for data exchange, system integration, and performance evaluation, the potential for AI and DT technologies to drive meaningful change in environmental sustainability remains constrained. To overcome these barriers, efforts must be made to develop standardized guidelines and best practices for the design, implementation, and evaluation of AI systems and AI-driven DT frameworks in sustainable smart built environments.

#### 6.4.5 Financial and institutional barriers

Financial and institutional barriers pose real challenges to the widespread adoption of AI and DT technologies for enhancing environmental practices in the built environment. The initial investment required to implement these technologies can be prohibitive, particularly for small- and medium-sized enterprises or property owners with limited financial resources. The interplay of financial and institutional factors with economic and industrial structures highlights the complexity of adoption rates and environmental outcomes [204]. In addition, many organizations lack the expertise or infrastructure necessary to integrate AI and DT systems into existing building operations. There is also a lack of financial incentives, subsidies, or support programs from governments and industry bodies to encourage the adoption of these technologies.

Institutional barriers, including resistance to change and the slow pace of regulatory approval processes, can further delay the implementation of AI and DT-driven solutions. As these technologies evolve rapidly, it is crucial for policy-makers and regulatory bodies to create a supportive environment that facilitates innovation while ensuring safety, fairness, and accountability. This includes creating policies that promote collaboration between public and private sectors, provide financial incentives, and support workforce development initiatives to build the necessary skills and expertise.

#### 6.4.6 Methodological and framework limitations

From a research perspective, there are several methodological limitations associated with the systematic review process employed in this study. Despite its comprehensive scope, this study faced several constraints. The review was limited to peer-reviewed articles published in English and retrieved from two major academic databases, which may have excluded relevant insights from non-English sources, region-specific studies, or non-indexed academic work. In addition, by concentrating on literature published between 2020 and 2025, earlier foundational studies that continue to shape the evolution of AI applications in SGZEBs may have been overlooked. The thematic synthesis and categorization of studies required interpretive judgment, which, despite methodological rigor, may have introduced subjectivity, particularly in classifying research spanning multiple building typologies or environmental sustainability dimensions.

Moreover, the exclusion of grey literature, including industry reports, technical standards, and professional white papers, may have omitted practical innovations and real-world applications that could further contextualize the academic findings. While the comparative analysis illuminated differences, synergies, and complementarities across building typologies and AI applications, the uneven availability and quality of metadata in some studies limited the granularity of the analysis, potentially leaving nuanced dynamics underexplored.

In terms of the framework, while the review has been comprehensive in its scope, the availability and quality of studies on the integration of AI and DTs in SGZEBs remain uneven. Many studies focus on isolated technologies or specific building typologies, and there is a lack of comprehensive, cross-cutting research that addresses the holistic integration of AI and DTs across different environmental metrics. As a result, the framework presented in this study is based on a synthesis of available literature that may be fragmented in terms of scope.

Furthermore, there is a lack of consensus on the most appropriate methodologies for evaluating the performance of AI and DT systems in the context of sustainability. Different studies employ various metrics and performance indicators, making it difficult to compare results across studies and draw definitive conclusions about the effectiveness of these technologies in achieving environmental targets. Developing standardized evaluation frameworks that can be consistently applied across different research contexts is essential for advancing the field and enabling more robust comparative analyses.

In conclusion, significant challenges, barriers, and limitations must be addressed to ensure the effective integration and widespread adoption of AI–DT integration in SGZEBs. These challenges require coordinated efforts from researchers, practitioners, policymakers, and industry stakeholders to overcome. Addressing these challenges can unlock the full potential of AI and DTs in building future sustainable smart urban environments.

### 6.5 Suggestions for future directions

The integration of AI and DT technologies in the development of environmentally sustainable smart built environments offers fascinating opportunities, but also poses several unresolved issues. While this study presents a comprehensive framework for leveraging these technologies, it is clear that further research and practical innovations are needed to overcome current barriers and unlock their full potential. This subsection outlines several key areas for future research, technological development, and policy advancement that will shape the evolution of SGZEBs in the years to come.

#### 6.5.1 Expanding real-world case studies, pilot projects, and inclusion criteria

One of the key recommendations for future research is the expansion of real-world case studies and large-scale pilot projects that test the effectiveness and scalability of AI and DT technologies in diverse building systems and urban contexts. Existing studies largely focus on theoretical models or small-scale applications that may not fully capture the complexities and challenges of implementing AI and DT systems in real-world settings, so there is a need for research that investigates how these technologies perform in real-world environments, across different climate zones, building typologies, and urban infrastructures. Longitudinal studies tracking the performance of AI- and DT-driven buildings over extended periods will provide invaluable insights into their long-term sustainability impacts, including energy savings, emissions reduction, resource optimization, and operational efficiency. These case studies will also help identify practical challenges related to system integration, data management, and performance evaluation, which can inform the development of more robust implementation strategies. Future endeavors should also prioritize large-scale implementations to better understand the practical implications of integrating these technologies at a city-wide level.

Prospective studies could benefit from broadening the inclusion criteria to encompass non-peer-reviewed and grey literature, earlier influential studies, and a range of indexed databases, while also adopting mixed-methods approaches to mitigate classification ambiguities. Such steps would offer a richer, more nuanced understanding of the evolving role of AI and DTs in advancing environmental goals across the built environment and support more actionable strategies for researchers, practitioners, and policymakers working towards sustainable smart built environments.

#### 6.5.2 Standardization of AI and DT integration

As highlighted in the challenges section, a lack of standardization and interoperability remains a significant barrier to the widespread adoption of AI and DT technologies. Continued research should prioritize the development of universal standards and protocols for the integration of AI, DTs, and other smart technologies in the built environment. This includes developing guidelines for data exchange, system interoperability, and performance measurement, which will facilitate collaboration between stakeholders across different sectors. Establishing these standards will both simplify the implementation process as well as promote the scalability of AI and DT systems across diverse building typologies and urban environments.

#### 6.5.3 Development of advanced AI models for environmental sustainability

AI plays a critical role in driving environmental sustainability through resource optimization, emissions control, and energy management. However, current AI models are often limited by their ability to account for the complexity and dynamic nature of urban environments. Future research is recommended to focus on the development of more advanced AI models that are capable of incorporating a broader range of environmental factors and socio-economic conditions. These models should be designed to optimize energy use while also supporting the broader goals of urban ecological restoration, circular economy integration, and social equity. Future models can play a more active role in advancing sustainable smart city agendas by enhancing the ability of AI or AI-driven DT to predict, adapt, and optimize across multiple sustainability dimensions.

#### 6.5.4 Integration of social and behavioral data

Another area for future exploration is the integration of social and behavioral data into AI and DT systems. While much of the current focus has been on technical optimization (e.g., energy use, emissions control), understanding human behavior and its impact on building performance is crucial for achieving the status of sustainable smart cities. For instance, occupant behavior, such as energy consumption patterns, waste management practices, and comfort preferences, can significantly influence the performance of AI and DT systems. Future research should explore how AI models can incorporate behavioral data, along with environmental sensors, to more accurately predict and optimize building operations. In addition, incorporating human-centered design principles into the development of sustainable and smart buildings can ensure that these technologies are user-friendly, promote sustainable lifestyles, and enhance occupant satisfaction.

#### 6.5.5 Ethical and governance frameworks for artificial intelligence in sustainability

As AI and AIoT systems become more integrated into sustainable smart cities [3,8,205,11,13,14,206], ensuring their ethical use and alignment with sustainability goals will be increasingly important. Additional investigations must focus on developing ethical and governance frameworks that guide the responsible deployment of AI technologies in the built environment. This includes addressing issues such as data privacy, algorithmic transparency, fairness, accountability, and equity. AI systems must be designed to avoid biases that could lead to unequal access to resources or unfair outcomes in energy distribution, urban management, and planning. Moreover, governance frameworks should facilitate collaboration between public and private stakeholders by ensuring that AI and AI-driven solutions are developed and deployed in a way that supports the broader social, environmental, and economic goals of sustainability.

#### 6.5.6 Policy development and incentive structures

To accelerate the adoption of AI and DT technologies in the built environment, there is a need for supportive policy development and incentive structures. Further research is encouraged to examine the role of government policies in promoting the integration of AI and DTs in sustainable smart building practices. This could include financial incentives, tax credits, subsidies for retrofitting existing buildings, and funding for pilot projects. Moreover, policy-makers should collaborate with industry stakeholders to create regulatory frameworks that promote innovation while ensuring environmental standards, safety, and fairness. In addition, policies that encourage the development of green infrastructure, renewable energy integration, and circular economy practices will further align AI and DT technologies with the global agenda for sustainable development.

#### 6.5.7 Education, training, and capacity building

The successful implementation of AI and DT technologies in the built environment requires a highly skilled workforce capable of developing, managing, and optimizing these systems. Upcoming investigations should examine the role of education and training in preparing professionals for the integration of AI and DTs in sustainable smart buildings. This includes the development of interdisciplinary programs that combine expertise in AI, environmental science, urban development, design, and engineering. Moreover, capacity-building initiatives aimed at upskilling existing professionals, such as building managers, architects, engineers, and policy-makers, will be essential for ensuring the widespread adoption of these technologies. Collaboration between academic institutions, industry leaders, and government bodies will be crucial for creating a pipeline of talent and fostering innovation in AI-driven sustainability solutions.

## 7 Conclusion

This study conducts a comprehensive systematic review of AI and AI-driven DT applications across SGZEBs. The aim is to provide a holistic understanding of how these advanced technologies enhance the environmental performance of SGZEBs by analyzing key related sustainability indicators. The study explores the extent to which AI and AI-driven DTs enable integrated, system-level strategies for enhancing environmentally sustainable smart practices in the built environment by synthesizing, comparing, and evaluating recent research studies. By addressing five research questions, the study provides comprehensive insights into how these technologies contribute to the broader goals of sustainable urban development by enhancing the environmental performance of SGZEBs.

### 7.1 Key findings and implications

Table 6 summarizes the key findings of the study across building typologies, highlighting how AI and AI–DT integration enhance environmental performance and sustainability outcomes.

**Table 6:** Key findings and implications of environmental solutions of AI and AI-driven DT in sustainable smart buildings.

**RQs**	**Typology**	**Key findings**	**Implications**
RQ1	SBs	- AI and ML optimize energy management and resource use. - Adaptive control strategies respond to environmental conditions and occupant behavior. - AI facilitates renewable energy integration through forecasting and storage optimization. - Occupant-centered designs balance comfort with sustainability goals. - Predictive system control anticipates operational needs.	- Enhances building operational efficiency and self-regulation. - Supports cleaner energy consumption and reduced environmental impact. - Ensures future-proof, sustainable performance.
RQ2	GBs	- AI enhances design and performance via predictive modeling, multi-objective optimization, and ML techniques. - Improves energy efficiency, reduces carbon emissions, lowers costs, and improves occupant comfort. - Accurate forecasting of heating, cooling, and energy demands minimizes inefficiencies. - Optimizes material and insulation use and guides resource-efficient decisions. - Strengthens project delivery via cost estimation, risk assessment, and hybrid expert-AI approaches.	- Promotes resource-efficient, low-emission building practices. - Accelerates design and compliance with sustainability standards. - Enhances cost management, risk mitigation, and reliable project delivery.
RQ3	ZEBs	- AI optimizes building design, HVAC, and smart materials for energy efficiency. - Facilitates integration of renewable energy sources (solar, wind) into distributed networks. - Enhances forecasting, real-time control, and fault detection. - Supports occupant comfort through neural network predictive control and data-driven optimization. - Improves NZEB performance with predictive energy analytics, smart-home systems, hybrid optimization, XAI transparency, multi-objective optimization, and smart grid/microgrid coordination.	- Ensures reliable, energy-efficient, and cost-effective high-performance buildings. - Supports net-zero and positive-energy goals. - Balances occupant comfort with sustainability targets.
RQ4	SGZEBs	- AI–DT integration enables adaptive control, predictive maintenance, and real-time performance optimization. - Enhances energy efficiency, reduces emissions, and supports occupant-centered design. - Balances energy production and consumption at building and district scales. - Continuous monitoring and predictive interventions improve energy efficiency, reduce carbon footprint, and enhance indoor environmental quality.	- Promotes systemic sustainability across building types. - Facilitates district- and city-scale energy optimization. - Strengthens urban resilience and environmental performance benchmarks.

With respect to RQ5, the novel integrated framework demonstrates that AI- and DT-enabled SGZEBs contribute to environmental sustainability, sustainable development, and sustainable smart cities across diverse domains. It positions AI and DTs as systemic enablers of environmentally sustainable smart built environments, emphasizing their cross-scale convergence in promoting carbon neutrality, circular economy principles, climate resilience, and environmentally regenerative urban strategies. The findings confirm that SGZEBs, guided by AI and DT frameworks, can align building-level performance with city-wide sustainability objectives, driving systemic transformations towards resource-efficient, climate-resilient, and ecologically adaptive urban environments. These insights complement Table 6 by highlighting the broader systemic impact of AI and AI–DT integration in advancing environmentally sustainable urban development.

### 7.2 Significance of the proposed framework for environmentally sustainable smart built and urban environments as enabled by artificial intelligence-digital twin integration

The proposed AI–DT framework carries significant implications for the transition towards environmentally sustainable smart built environments and cities:

Principle-based reinforcement across building typologies: The framework establishes a cycle of reciprocal reinforcement among SGZEBs. Data-driven control in smart buildings enhances the sustainability metrics of green buildings by enabling precise monitoring, adaptive system management, and performance optimization. In turn, the circularity and resource-efficiency principles of green buildings provide the foundation for self-sufficiency and closed-loop strategies in ZEBs. Meanwhile, renewable energy integration and balancing mechanisms in ZEBs feed back into smart buildings by strengthening adaptive intelligence, occupant-centered optimization, and system resilience. This continuous principle-based feedback loop ensures that progress in one typology directly amplifies performance and sustainability outcomes in the others.

Advancement of environmental performance benchmarks: The framework enables ongoing monitoring, predictive interventions, and evidence-based decision-making by embedding AI–DT intelligence into the core of building operations. This moves buildings beyond static compliance with environmental standards towards a dynamic capacity to meet evolving benchmarks in energy efficiency, resource conservation, and occupant well-being.

Transition from isolated assets to systemic urban actors: Buildings are no longer standalone entities but interconnected nodes within a larger urban environmental network. Their operations feed into broader urban flows, including energy grids, waste systems, water systems, and material cycles, which enable regenerative urban ecosystems that are both adaptive and resource-efficient.

Continuous learning and adaptation through AI–DT feedback loops: The iterative cycle of AI learning from DT data, refining models through simulations, and reapplying insights to real-world systems ensures that the built environment becomes a self-improving system. This adaptability is critical for addressing dynamic environmental pressures, such as ecological degradation, resource scarcity, climate change, and urbanization.

Contribution to broader sustainability and climate agendas: At the macro scale, the framework provides a strategic pathway for advancing global goals, including carbon neutrality, climate resilience, circular economy adoption, and SDGs. Its ability to integrate micro-level performance optimization with systemic urban planning positions it as a transformative model for the future of sustainable smart cities.

In conclusion, this study underscores the significant impact and innovative role of AI and DT technologies in advancing environmental sustainability in the context of SGZEBs by providing an integrated framework. The findings highlight the importance of adopting a systemic approach that encompasses both micro-level building performance and macro-level city-scale sustainability outcomes. AI and DTs represent a promising pathway for creating more energy-efficient, resilient, and ecologically responsible cities, ultimately contributing to the realization of global sustainability goals. With continued research, innovation, and collaboration across sectors, these technologies have the potential to reshape the future of environmentally sustainable smart built and urban environments.

## CRediT authorship contribution statement

**Simon Elias Bibri:** Writing - Review & Editing, Writing - Original Draft, Visualization, Software, Methodology, Investigation, Formal Analysis, Data Curation, Conceptualization. **Jeffrey Huang:** Writing - Review & Editing, Conceptualization.

## Declaration of competing interest

The authors declare that they have no known competing financial interests or personal relationships that could have appeared to influence the work reported in this paper.
